# Interventions for promoting physical activity among adolescents in school settings: systematic review and meta-analysis

**DOI:** 10.7189/jogh.16.04112

**Published:** 2026-03-27

**Authors:** Marium Moin, Zainab Farhan, Syeda Kanza Naqvi, Zohra Lassi, Jai K Das

**Affiliations:** 1 Department of Paediatrics and Child Health, Aga Khan University, Karachi, Pakistan; 2 Institute for Global Health and Development, Aga Khan University, Karachi, Pakistan; 3 Robinson Research Institute, The University of Adelaide, Adelaide, Australia

## Abstract

**Background:**

Physical inactivity among adolescents is a significant challenge contributing to rising cases of obesity, noncommunicable diseases (NCD), and mental health challenges. A viable strategy is the promotion of physical activity (PA) in the school setting. We conducted a systematic review and meta-analysis to assess the efficacy of school-based physical activity interventions, compared to control conditions, in improving activity outcomes in adolescents.

**Methods:**

We followed PRISMA guidelines and searched Scopus, Embase, CINAHL, PubMed, and Cochrane from 2000 onwards for relevant studies in English. We included randomised controlled trials (RCTs) involving adolescents aged 10–19 years with interventions in school-based settings and outcomes including activity, fitness, sedentary behaviours, and body mass index (BMI). We conducted a meta-analysis of included studies, assessed them using the Risk of Bias (ROB) 2 tool from the Cochrane handbook, and evaluated evidence quality through the GRADE framework.

**Results:**

The search yielded 30 629 records, of which 90 studies including approximately 17 000 adolescents met the inclusion criteria. Interventions included multicomponent programmes, activity lessons, educational awareness sessions, and after-school activities. In the short term (0–6 months), school-based interventions significantly increased physical activity levels (standardised mean difference (SMD) = 0.41, 95% confidence interval (CI) = 0.23–0.59; low-certainty evidence). However, these improvements were not sustained beyond six months of follow-up, with pooled analyses showing no significant long-term effects on overall activity, sedentary behaviour, or BMI.

**Conclusions:**

School-based interventions can modestly increase adolescents’ physical activity in the short term, but their effects diminish over time. Sustaining behavioural change may require long-term, system-level strategies that involve schools, families, and communities. Overall, the certainty of evidence was low to moderate, and findings should be interpreted with caution due to high heterogeneity across studies.

Adolescence is a period where individuals undergo significant physical, emotional, and cognitive changes [1]. Children aged 5–17 are recommended to engage in at least 60 minutes of daily moderate-to-vigorous aerobic activity such as brisk walking, jogging and cycling as well as muscle and bone strengthening exercises spread throughout the week [2–4] which are effective in preventing and managing non-communicable diseases (NCDs) such as heart disease, stroke, diabetes, and various cancers [1]. Regular physical activity also contributes to the prevention of hypertension, supports healthy body weight, and enhances mental health, quality of life, and overall well-being [4,5]. Physical inactivity on the other hand stands as a primary contributor to the prevalence of NCDs and mortality globally [6], significantly increasing the risk of developing cancer, cardiovascular conditions, stroke, and diabetes by approximately 20–30% [7]. According to a 2016 global study, 81% of children aged 11–17 years were not sufficiently physically active [2]. Prevalence of insufficient physical activity varies across country income levels: 84.9% in low-income countries (LICs), 79.3% in lower-middle-income countries (LMICs), 83.9% in upper-middle-income countries (UMICs), and 79.4% in high-income countries (HICs) [8].

Schools are widely seen as ideal settings for promoting PA because they can systematically reach large, diverse adolescent populations [9–11]. School-based programmes can influence adolescent behaviour by targeting both individual-level factors (education, skill-building, motivation) and environmental factors (policies, facilities, social support). Many successful interventions draw on established behaviour-change theories. For example, Social Cognitive Theory and Self-Determination Theory emphasise building students’ self-efficacy, autonomy, and enjoyment of exercise, providing a theoretical basis for intervention design [9,10]

Effective school-based interventions can provide equitable opportunities for adolescents to engage in physical activity, regardless of their socio-economic background [11], while the key barriers to physical activity in schools include academic pressure, social changes, and lack of infrastructure [12,13].

It is important to note that most existing evidence on the effectiveness of school-based interventions originates from HIC and UMIC contexts, with limited data from LIC and LMIC settings with a previous review reported that only 3.1% of included studies were conducted in LMICs [14]. Therefore, this systematic review primarily consolidates evidence from high- and upper-middle-income countries, while also highlighting the scarcity of LMIC data as an important evidence gap. This systematic review evaluates the effectiveness of school-based interventions aimed at promoting adolescents’ physical activity, fitness, and body mass index (BMI), and emphasises the need for future research to address this gap especially in low resource settings.

## METHODS

This systematic review follows the preferred reporting items for systematic reviews and meta-analysis (PRISMA) guidelines [15] (File S1 in the **Online Supplementary Document**).

### Objectives

The primary objective of this systematic review is to assess the impact of school-based interventions on promoting physical activity among adolescents (10–19 years) and reducing sedentary time while the secondary objective is to assess its impact on physical fitness and BMI.

### Eligibility criteria

We included all published randomised controlled trials (RCTs) where the intervention aimed at increasing physical activity (PA) compared to a control group with no intervention, as RCTs represent the highest standard of evidence for evaluating intervention effectiveness. To ensure methodological rigor and contemporary relevance, only studies published from the year 2000 onwards conducted on adolescents aged 10–19 years old, and studies where the outcome was measured quantitatively were included. The review was limited to English-language publications due to the lack of translation resources (**Table 1**).

**Table 1:** Inclusion/exclusion criteria

Inclusion criteria
Adolescents aged 10–19 y
Any interventions implemented within school and college settings aimed at increasing physical activity among adolescents; curriculum physical activity, structured physical education programmes, extracurricular physical activities, integrated classroom-based activities, or school and college policy changes promoting physical activity
Studies with a control group/comparator; no intervention, standard school, and college curriculum without additional physical activity components so that the differences between the two groups was only physical activity
Studies where the outcome was measured quantitatively
Relevant study design; Randomised controlled trials (RCT)
**Exclusion criteria**
Studies in languages other than English
Studies conducted before the year 2000
Studies where the patient population are obese adolescents exclusively
Studies without a comparison group
Studies where the outcome is measured qualitatively
Outcome not clearly defined

### Outcomes

The primary outcomes included proportion of students meeting recommendations for light physical activity (LPA), moderate-intensity physical activity (MPA), moderate to vigorous physical activity (MVPA), vigorous physical activity (VPA), duration of physical activity, sedentary time and secondary outcomes included physical fitness, and BMI. We included studies where the data was available both as quantitative and categorical variables. Only studies that quantitatively measured outcomes were included in the review, these included physical activity and sedentary time measured using accelerometers and pedometers, physical fitness assessed through different tests such as the shuttle test, handgrip test, *etc*.

### Search strategy

We formulated the search strategy using the population, intervention, control, and outcome (PICO) methodology, basing it on medical subject headings (MeSH) terms and keywords, but without restrictions by the outcome to retain a broader search (File S2 in the **Online Supplementary Document**)

We searched Scopus, Embase, CINAHL, PubMed and Cochrane on 12 June 2024, for studies from 2000s onward to ensure that we use the most recent evidence available in the English language. We searched references of included studies and previous systematic reviews to find any missed studies.

We exported our search results into Covidence for independent title/abstract and full-text screening by two reviewers (MM and ZF) based on predefined eligibility criteria. Any conflict was resolved by a third reviewer (SKN).

### Data extraction and management

Two reviewers (MM and ZF) independently extracted relevant data from all included full text studies using a preformed data extraction sheet on Microsoft excel. For each included study, data were extracted on the study location, sample size, gender distribution (number of boys and girls), mean values and their standard deviations, confidence intervals, the type of intervention, outcomes, limitations and funding of the study. Inconsistences between the two reviewers were resolved by consensus after consulting with a third reviewer (SKN). Post intervention values adjusted for baseline differences, along with confidence intervals (95%) or standard deviations (SDs), were extracted whenever available. If only changes from baseline are reported, these differences, along with CIs or SDs, were also extracted. Physical activity outcomes were extracted according to the definitions provided by study authors. Given substantial variability in accelerometer models, wear-time algorithms, epoch lengths, and intensity cut-points, reclassification of outcomes using a uniform set of thresholds (*e.g*. Evenson, Freedson, Troiano) was not feasible without access to raw accelerometry files. Therefore, harmonisation was conducted within conceptually similar constructs only. Time-based intensity outcomes (*e.g*. minutes/d of MVPA) were considered comparable across studies and were synthesised using standardised mean differences (SMDs) when numerical units differed.

To avoid pooling non-commensurate constructs, steps per day and counts per minute were treated as distinct outcomes and analysed separately. These results are presented as separate forest plots (File S5 in the **Online Supplementary Document****)**. Two independent reviewers (MM, ZF) assessed the methodological quality of the included RCT’s (individual or cluster) using the ROB 2 tool and give an overall risk of bias judgement (low, high and some concerns) [16]. The ROB 2 tool assesses risk of bias across six domains; Sequence generation, Allocation concealment, blinding of participants and personnel, Blinding of outcome assessors, Incomplete outcome data and selective reporting. Any discrepancies in assessments were resolved through discussion. In cases where sufficient information is lacking, trial authors were contacted to obtain missing data regarding the 'Risk of bias' items [17].

### Statistical analysis

The meta-analysis was conducted using RevMan version 5.4 (The Cochrane Collaboration, London, UK). Continuous outcomes *(e.g*. physical activity levels, BMI) were summarised using SMDs with corresponding 95% CIs, calculated from extracted means and SDs reported in each study. When studies reported outcomes in different units (*e.g*. minutes of activity, steps per day, or accelerometer counts), data were harmonised to reflect comparable constructs of physical activity through the use of SMDs. When both endpoint and change-from-baseline data were available, endpoint values were prioritised for consistency. For dichotomous outcomes (*e.g*. proportion of participants meeting PA recommendations), event counts and total sample sizes were extracted to calculate pooled risk ratios (RRs) with 95% CIs. This process ensured transparency and reproducibility in data synthesis across studies using diverse outcome metrics and measurement tools. If a study included more than one intervention arm, participant numbers were evenly divided among relevant arms for both continuous and dichotomous outcomes to avoid double-counting. Statistical heterogeneity was assessed using the *I*^2^ statistic, and a random-effects model was applied to account for between-study variability.

Several included studies were cluster RCTs; where authors reported effect estimates that had already been adjusted for clustering (*e.g*. using mixed models, generalised estimating equations, or design effects), we extracted the adjusted values directly and for studies that did not clearly state whether clustering had been accounted for, we retained the reported estimates but noted these as unadjusted. As these trials had relatively small sample sizes and limited weighting in the meta-analysis, they were unlikely to materially influence the pooled effects. To assess the potential impact of unadjusted cRCTs, we performed a sensitivity analysis excluding these studies.

We conducted sensitivity analyses by excluding studies with high risk of bias, influential outliers, or unadjusted cluster-randomised trials. These analyses examined whether the pooled effect estimates were materially affected by study quality, sample size, or methodological differences. The results of the sensitivity analyses were consistent with the primary meta-analyses, supporting the stability and reliability of our overall conclusions.

### Quality assessment (GRADE)

We used the GRADE approach to assess the overall certainty of evidence for both primary and secondary outcome measures. This framework considers factors related to both internal and external validity to determine our confidence in the effect estimates presented. Any discrepancies in assessment were resolved through discussion. For each outcome, we categorised the certainty of evidence as very low, low, moderate, or high based on the GRADE domains as described in Chapter 14 of the Cochrane Handbook for Systematic Reviews of Interventions [18]. We downgraded the certainty of evidence when studies showed inconsistency in results, imprecision due to small sample sizes or wide confidence intervals, and potential publication bias. We made GRADE evidence profiles and Summary of Findings (SoF) tables (File S4 in the **Online Supplementary Document**). A concise summary of the GRADE findings has also been added in the Results section to enhance interpretability.

## RESULTS

**Figure 1:**
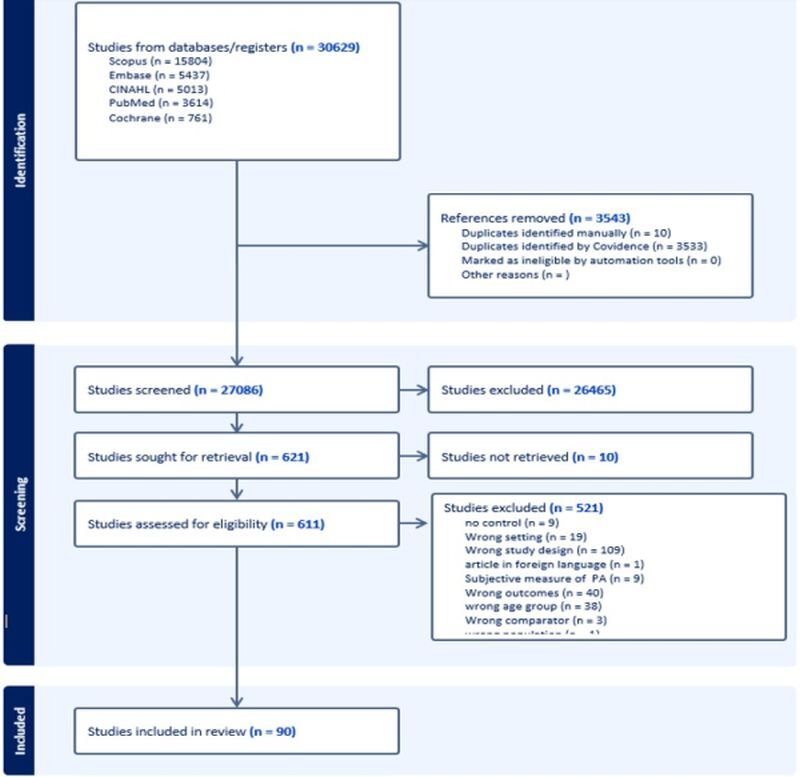
PRISMA flowchart.

The search strategy yielded a total of 30 629 studies of which 3543 were identified as duplicates. The remaining 27 086 studies were screened and 621 were sought for full texts, of which 10 could not be retrieved. A total of 611 studies were assessed for eligibility by conducting full text review, 521 of these studies were excluded and the remaining 90 [19–108] studies are included in this review (File S3 in the **Online Supplementary Document****)**. PRISMA flowchart illustrates this information (**Figure 1**).

### Study characteristics

This review included a total of 90 studies [19–108], with 84 conducted in HICs, six in UMICs Iran [40], China [66,97], Turkey [19], Ecuador [23], Brazil [85], and Mexico [43] and one in a LMIC, Ghana [79]. The study designs comprised 38 individual RCTs and 53 cluster randomised controlled trials (cRCTs), with sample sizes ranging from 31 to 17 000 participants (File S3 in the **Online Supplementary Document****)**. The interventions varied widely and included educational, health promotion, counselling, and management approaches centred around physical activity. Additionally, interventions focused on teacher training, providing educational materials to students, teachers, and parents, as well as policy development and environmental changes aimed at promoting physical activity among adolescents. A summary of the interventions is provided (**Table 2**).

**Table 2:** Summary of interventions

Intervention type	Theoretical framework	Focus	Duration	Mode of delivery	Delivery agents
Multi-component programmes (48 studies)	Social cognitive theory, behavioural change models	Physical activity, nutrition, environmental changes (*e.g*. Active classrooms)	3–12 mo	Classroom-based sessions, counselling	Teachers, school staff, counselors
Physical activity lessons (29 studies)	None explicitly stated	Physical activity during school hours	6–12 mo	Structured PE lessons	Physical education instructors
Educational/awareness lessons (11 studies)	Health belief model, information-motivation-behavioural skills model	PA education, behaviour change, reducing sedentary behaviour	3–6 mo	Classroom lessons, workshops	Teachers, peer educators
After-school activities (2 studies)	None explicitly stated	Physical activity outside school hours	3–9 mo	Sports, aerobic sessions, team games	Sports coaches, school staff

### Primary outcomes

#### Continuous outcome

**Figure 2:**
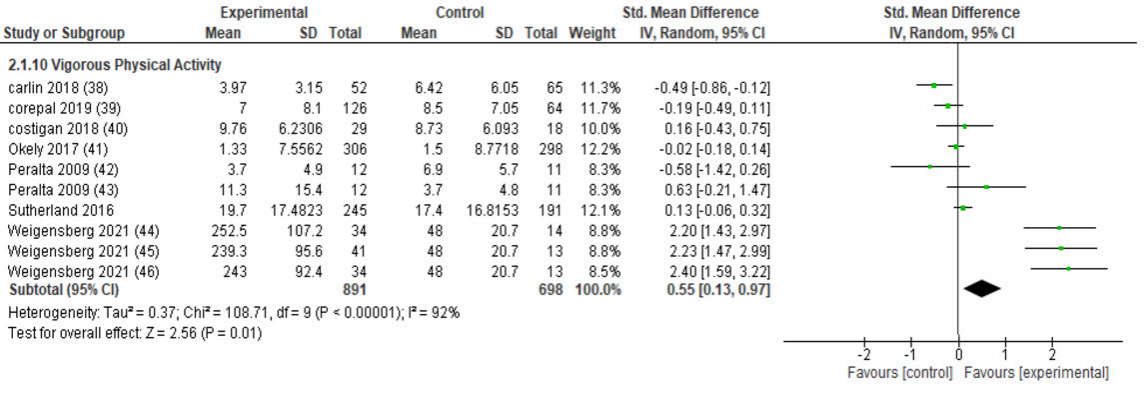
Forest plot- vigorous physical activity.

School-based interventions were associated with a statistically significant increase in vigorous physical activity. Across seven studies involving 1589 participants, the pooled effect size was SMD = 0.55 (95% CI = 0.13, 0.97; low certainty of evidence; *I*^2^ = 92%) (**Figure 2**). In contrast, no significant differences were observed for light physical activity (six studies; SMD = −0.04, 95% CI = −0.13, 0.05; high certainty), moderate physical activity (seven studies; SMD = 0.01, 95% CI = −0.16, 0.18; high certainty), or moderate-to-vigorous physical activity (20 studies; SMD = 0.02, 95% CI = −0.06, 0.11; low certainty, *I*^2^ = 74%), indicating that interventions had minimal impact on overall activity levels of lower intensity.

Eight RCTs assessed sedentary time, with no significant reductions observed (SMD = −0.06, 95% CI = −0.12, 0.01; 4757 participants, *I*^2^ = 15%) (Figure 20 in File S5 in the **Online Supplementary Document**).

When examining additional physical activity metrics, 13 studies using counts per minute showed a small but significant increase of 23 counts per minute (MD = 23.11 counts/min; 95% CI = 5.78, 40.44; Z = 2.61, *P* = 0.009; low certainty; *I*^2^ = 95%). Removing the outlier study [71], the effect remained statistically significant and of very similar magnitude to the original estimate (22.56 counts/min; 95% CI = 6.50, 38.62) Seven studies measuring steps per day showed a significant positive effect of school-based interventions compared with controls (MD = 2424.05 steps/d, 95% CI = 854.76, 3993.34, *P* = 0.002). However, heterogeneity was very high (*I*^2^ = 91%), indicating substantial variability across studies in both intervention effects and measurement approaches. Removing the outlier study [61] reduced the pooled effect size only slightly (MD = 2214.83 steps/d, 95% CI = 664.16, 3765.51) and the intervention effect remained statistically significant.

These findings suggest that interventions can meaningfully increase overall daily activity, particularly for higher-intensity movement (File S5 in the **Online Supplementary Document**).

#### Dichotomous outcome

**Figure 3:**
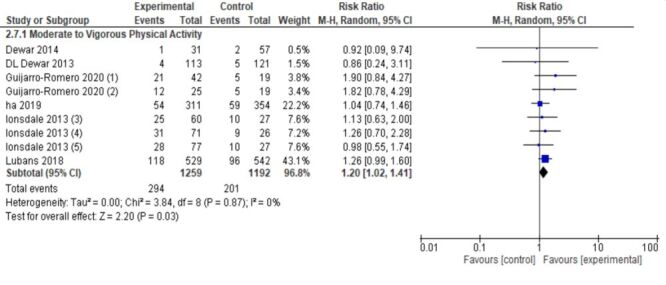
Forest plot-moderate to vigorous physical activity.

Six trials evaluated the impact of interventions on achieving recommended levels of moderate-to-vigorous physical activity (MVPA). The pooled effect indicated a 20% higher likelihood of meeting MVPA recommendations in intervention groups compared to controls (RR = 1.20; 95% CI = 1.02, 1.41; 2451 participants; high certainty) (**Figure 3**).

### Secondary outcome

Fitness outcomes showed modest improvements; There was a statistically significant increase in the cardiorespiratory fitness (SMD = 0.20, 95% CI = 0.01, 0.39; low certainty), and maximal oxygen uptake (VO_2_ max) (SMD = 0.98, 95% CI = 0.45, 1.51; low certainty), suggesting that interventions can enhance aerobic capacity. Conversely, aerobic fitness measured by distance run was slightly better in control groups (SMD = −0.24; 95% CI = −0.43, −0.05; low certainty). Although there was high heterogeneity across all these three measures which limits the confidence in these results.

Regarding BMI, pooled analyses indicated no significant difference between the intervention and control groups (SMD = 0.20, 95% CI = −0.04, 0.44), suggesting that school-based interventions had minimal impact on weight outcomes during the study periods (Figure 19 in File S5 in the **Online Supplementary Document****).**

### Subgroup analysis

We conducted subgroup analyses based on type of intervention, duration of follow-up, and World Bank analytical classification (**Table 3**). The results suggest that the effectiveness of school-based interventions varies by intervention type and duration. Multicomponent interventions, which combined physical activity with educational or environmental components, were associated with a significant increase in physical activity levels (SMD = 0.46, 95% CI = 0.27, 0.65), interventions consisting solely of physical activity or education-based lessons also had an impact on physical fitness (SMD = 0.56, 95% CI = 0.16, 0.96).

**Table 3:** Subgroup analysis

Difference in intervention subgroup analysis										
	**Multi-component intervention, No. of studies: 48**		**Physical activity/education lessons, No. of studies: 29**			**Educational awareness lessons, No. of studies: 11**			**After school activity programmes, No. of studies: 2**		
Physical activity	SMD = 0.46; 95% CI = 0.27, 0.65; *I*^2^ = 90%		SMD = 0.09; 95% CI = −0.04, 0.22; *I*^2^ = 90%			SMD = 0.37; 95% CI = 0.13, 0.62; *I*^2^ = 84%),			SMD = 0.34; 95% CI = 0.12, 0.55; *I*^2^ = 71%).		
Sedentary time	NA		SMD = −0.03; 95% CI = −0.11, 0.04; *I*^2^ = 14%			SMD = 0.04; 95% CI = −0.19, 0.27; *I*^2^ = 0%			SMD = −0.19; 95% CI = −0.33, −0.04; *I*^2^ = 0%		
Physical fitness	SMD = −0.05; 95% CI = −0.17, 0.07; *I*^2^ = 77%		SMD = 0.56; 95% CI = 0.16, 0.96; *I*^2^ = 91%			SMD = 0.74; 95% CI = 0.62, 0.87; *I*^2^ = 0%			SMD = −0.17; 95% CI = −0.75, 0.41		
Body mass index	SMD = 0.62; 95% CI = −0.03, 1.27; *I*^2^ = 99%),		SMD = 0.01; 95% CI = −0.11, 0.12; *I*^2^ = 0%			SMD = 0.07; 95% CI = −0.07, 0.22			SMD = −0.04; 95% CI = −0.58, 0.51; *I*^2^ = 46%		
**Duration of follow-up subgroup analysis**										
	**0–6 mo follow-up**	**7–12 mo follow-up**		**13–20 mo follow-up**	**21–24 mo follow-up**			**>2 y follow-up**		**4 y follow-up**
Physical activity	SMD = 0.41; 95% CI = 0.23, 0.59; *I*^2^ = 88%. No. of studies: 17	SMD = 0.02; 95% CI = −0.13, 0.17; *I*^2^ = 88%. No. of studies: 7		SMD = 0.06; 95% CI = −0.09, 0.21; *I*^2^ = 89%. No. of studies: 4.	SMD = 0.05; 95% CI = −0.09, 0.20; *I*^2^ = 77%. No. of studies: 2			NA		SMD = −0.01; 95% CI = −0.18, 0.16; *I*^2^ = 0%. No. of studies: 1
Sedentary time	SMD = −0.00; 95% CI = −0.22, 0.22; *I*^2^ = 0%. No. of studies: 2.	SMD = −0.05; 95% CI = −0.14, 0.04; *I*^2^ = 32%. No. of studies: 4.		SMD = −0.03; 95% CI = −0.19, 0.13; *I*^2^ = NA. No. of studies: 1	NA			NA		SMD = −0.17; 95% CI = −0.45, 0.11; *I*^2^ = 64%. No. of studies: 1.
Physical fitness	SMD = 0.58; 95% CI = 0.25, 0.91; *I*^2^ = 91%. No. of studies: 12.	SMD = 0.11; 95% CI = −0.30, 0.51; *I*^2^ = 97%. No. of studies: 3		NA	NA			SMD = 0.37; 95% CI = 0.18, 0.56; *I*^2^ = 93%. No. of studies: 3		NA
Body mass index	SMD = 0.00; 95% CI = −0.09, 0.08; *I*^2^ = 0%. No. of studies: 12	SMD = 0.47; 95% CI = −0.17, 1.12; *I*^2^ = 99%. No. of studies:		NA	SMD = 0.22; 95% CI = −0.03, 0.46; I^2^ = 97%. No. of studies:3			NA		NA
Physical activity	RR = 1.16; 95% CI = 0.93, 1.43; *I*^2^ = 0%. No. of studies: 4			RR = 1.26; 95% CI = 0.99, 1.60; *I*^2^ = 0%. No. of studies: 2				RR = 0.86; 95% CI = 0.24, 3.11; *I*^2^ = 0%. No. of studies: 1		
Sedentary time	RR = 0.90; 95% CI = 0.73, 1.11; *I*^2^ = 0%. No. of studies: 3			RR = 1.01; 95% CI = 0.85, 1.19; *I*^2^ = 0%. No. of studies: 1				NA		
**World Bank analytical classification subgroup analysis**										
	**High income countries**						**Upper middle-income countries**				
Body mass index	SMD = 0.25; 95% CI = −0.02, 0.51; *I*^2^ = 97%. No. of studies: 22						SMD = −0.14; 95% CI = −0.32,0.05; I^2^ = 61%. No. of studies: 3				
Physical activity	RR = 1.20; 95% CI = 1.02, 1.41; *I*^2^ = 0%. No. of studies: 6						RR = 1.05; 95% CI = 0.43, 2.55; *I*^2^ = 0%. No. of studies: 1				
Sedentary time	RR = 0.98; 95% CI = 0.85, 1.12; *I*^2^ = 0%. No. of studies:3						RR = 0.88; 95% CI = 0.57, 1.35; *I*^2^ = 0%. No. of studies: 1				

SMD – standard mean deviation, CI – confidence interval, *I*^2^ – heterogeneity, RR – risk ratio, NA – not applicable

Short-term interventions (0–6 months) demonstrated statistically significant improvements in both physical activity (SMD = 0.41, 95% CI = 0.23, 0.59) and physical fitness (SMD = 0.58, 95% CI = 0.25, 0.91). However, there were no significant effects on sedentary behaviour or BMI at any follow-up duration.

Subgroup analysis based on the World Bank analytical classification revealed generally similar effects across country income levels, except those high-income countries showed a significant increase in physical activity (RR = 1.20; 95% CI = 1.02, 1.41). These finding highlights that while school-based interventions can be effective across contexts, there is more evidence from higher-income settings.

### Risk of bias

Risk of bias was assessed using the Cochrane ROB 2 tool across six domains: sequence generation, allocation concealment, blinding of participants and personnel, blinding of outcome assessors, incomplete outcome data, and selective reporting. Overall, 53% of studies were rated as low risk of bias, 46% as having some concerns, and 1% as high risk. The most frequent sources of bias were unclearly described sequence generation methods and insufficient blinding of participants or personnel (File S5 in the **Online Supplementary Document**).

### GRADE

Based on the GRADE assessment, the certainty of evidence across outcomes ranged from high to very low. Evidence for vigorous physical activity and light physical activity outcomes was rated as low to moderate certainty, mainly downgraded for inconsistency (high heterogeneity) and risk of bias. Evidence for moderate-to-vigorous physical activity and sedentary behaviour was rated as low certainty, downgraded for imprecision and inconsistency across studies. No outcomes were upgraded (File S4 in the **Online Supplementary Document**).

### Sensitivity analysis

In our review, most cRCTs reported analyses that accounted for clustering effects. The few studies that did not explicitly provide adjusted estimates contributed minimally to the overall weighting due to their small sample sizes. Sensitivity analysis excluding these unadjusted cRCTs yielded results consistent with the primary analysis, indicating that the pooled effect estimates are robust and not materially affected by clustering-related imprecision. To address heterogeneity, we conducted sensitivity analyses excluding high-risk-of-bias studies and influential outliers, which showed that the overall conclusions remained consistent.

## DISCUSSION

The primary objective of this review was to assess the effectiveness of school-based interventions in increasing PA among adolescents aged 10–19 years, while also aiming to reduce BMI, decrease sedentary time, and promote physical fitness. Overall, the findings indicate that these interventions produced modest, selective improvements in specific physical activity measures rather than broad, sustained effects across all outcomes.

For continuous outcomes, there was a statistically significant increase in physical activity when measured as counts per minute (SMD = 0.31; 95% CI = 0.08, 0.53; low certainty) and steps per day (SMD = 0.63; 95% CI = 0.23, 1.03; low certainty), as well as for vigorous physical activity (SMD = 0.55; 95% CI = 0.13, 0.97; low certainty) in the intervention groups compared to controls. However, no significant changes were observed for light, moderate, or moderate-to-vigorous physical activity overall, suggesting inconclusive results and are consistent with Love et al. [109], particularly regarding MVPA outcomes.

Previous reviews examining specific intervention types have yielded findings consistent with this current review. For example, Masini et al. [110] conducted a systematic review of 22 studies on classroom-based active breaks and reported a small, non-significant increase in moderate-to-vigorous physical activity (MVPA) of +3.29 minutes per day (95% CI = −0.15, 8.75). However, that review focused solely on in-class activity, whereas the present review evaluates full-day physical activity outcomes. Any difference that was observed was not sustainable in the long term, as seen in the follow-up subgroup analysis.

Substantial heterogeneity exists across studies in terms of accelerometer device type, epoch length, wear-time algorithms, and intensity cut-points, all of which can influence classification of physical activity intensity. To minimise the risk of combining non-comparable metrics, we separated steps per day and counts per minute into independent analyses and pooled time-based intensity outcomes only when they reflected conceptually similar constructs defined consistently across studies.

For dichotomous outcomes, only MVPA showed a statistically significant increase in recommended activity levels (RR = 1.20; 95% CI = 1.02, 1.41), indicating that while some adolescents reached target activity levels, the average intensity and duration of total daily activity remained largely unchanged.

Regarding sedentary behaviour and BMI, pooled analyses indicated no significant reductions (SMD = −0.06, 95% CI = −0.12, 0.01) for sedentary time and (SMD = 0.20; 95% CI = −0.04, 0.44) for BMI. Interventions included strategies such as standing desks, active commuting promotion, screen time reduction, PA sessions, nutritional education, and behavioural programmes, yet these did not yield substantial changes over the follow-up period. This aligns with previous evidence suggesting that PA alone often has a minor effect on BMI without complementary dietary interventions [111].

Secondary outcomes revealed small but statistically significant improvements in cardiorespiratory fitness (SMD = 0.20; 95% CI = 0.01, 0.39) and VO_2_ max (SMD = 0.98; 95% CI = 0.45, 1.51) in intervention groups. Aerobic fitness was slightly better in control groups (SMD = −0.24; 95% CI = −0.43, −0.05), and overall heterogeneity and low certainty of evidence warrant cautious interpretation. These findings suggest that structured exercise sessions may yield short-term fitness gains, but effects are not consistent across studies.

Subgroup analyses provided additional insights. Multicomponent interventions were most effective in increasing physical activity (SMD = 0.46; 95% CI = 0.27, 0.65), while educational or PA-focused lessons primarily improved physical fitness (SMD = 0.56; 95% CI = 0.16, 0.96). Short-term interventions (0–6 months) consistently showed improvements, whereas longer follow-ups did not maintain significant effects. No intervention type significantly impacted sedentary behaviour or BMI. These findings support the evidence that combining school-based, family, and community components may enhance activity outcomes [112].

Most included studies were conducted in high and upper-middle income countries, limiting generalisability to low-resource settings. The observed heterogeneity suggests that intervention effects vary across cultural and socioeconomic contexts, highlighting the need for trials in low and middle-income countries to strengthen external validity.

In summary, school-based interventions can produce meaningful short-term gains in vigorous activity and fitness, but effects on overall daily activity, sedentary time, and BMI are limited. Evidence from multicomponent and short-term programmes appears most robust, providing guidance for future intervention design and implementation.

### Strengths and limitations

A major strength of this review is the fact that only studies where the outcomes were measured objectively were included. This included using body mass index for weight and accelerometers and pedometers for physical activity. This allows the review to avoid bias that arises with self-reported data and makes it more credible and accurate. Another strength was using only randomised controlled trials which represent the highest standard of evidence in clinical research. Another strength is the inclusion of studies from a diverse range of countries allowing for a global perspective on the effectiveness of school-based intervention.

We also encountered multiple limitations. Most of the studies were from high-income and upper middle-income countries with only one included study [79] being from a lower income country reducing the generalisability of the outcomes. In addition, the review was limited to English language publications due to the lack of translation resources, which may have introduced language bias. However, multiple international databases were searched to minimise the potential impact of this limitation. Another limitation was the fact that many studies had outcomes measured subjectively and were hence excluded. Furthermore, restricting inclusion to RCTs published after 2000 ensured methodological rigor and relevance to current practice but may have excluded quasi-experimental or natural experiments that could offer useful insights, especially in educational settings. Limitations like high heterogeneity, lack of blinding, high attrition rates and missing data make interpretation of the results difficult. Although SMDs allowed synthesis of MVPA outcomes measured using different numerical scales, this approach does not fully address underlying device- and protocol-related variation. We acknowledge this limitation and recommend greater standardisation in accelerometer protocols including cut-points, epoch lengths, and wear-time requirements to improve comparability in future research.

The shorter follow-up of the included studies limits the ability to evaluate long term sustainability of the intervention's effects. Studies also lacked standardisation in reporting outcomes which makes direct comparison challenging.

## CONCLUSIONS

This review assessed the impact of school-based interventions on physical activity, fitness, body mass index, and sedentary time. Our review found modest improvements in vigorous physical activity and certain fitness outcomes, but no significant differences were observed for overall physical activity, sedentary time, or BMI. The study concluded that moderate activity alone is insufficient to significantly boost physical activity levels. Most included studies were conducted in high and upper-middle income countries, limiting generalisability to low-resource settings. Schools should prioritise structured, vigorous physical activities to improve students’ cardiovascular health and engagement. Also, regular monitoring and long-term evaluation of interventions can ensure effectiveness. Significant heterogeneity across studies and generally low to moderate certainty of evidence further limits the strength of conclusions. It also suggests using booster activities, such as seasonal sports or community fitness challenges, to sustain students’ interest in physical activity. Future research should prioritise long-term, context-specific interventions, particularly in low and middle-income countries, to assess sustainability and effectiveness.

These findings suggest that school-based interventions may contribute modestly to enhancing adolescents’ physical activity levels, but the overall magnitude and durability of these effects remain uncertain. Standardised outcome measures, longer follow-up periods, and inclusion of diverse populations are recommended to enhance comparability and global relevance. Given the substantial heterogeneity across studies and predominantly high-income settings, the generalisability of results to low and middle-income contexts is limited. Future interventions may benefit from incorporating structured and engaging physical activity components; however, more evidence is needed to determine the optimal intensity and delivery strategies. While multicomponent or structured interventions may offer the most consistent benefits, current evidence does not support prescriptive recommendations regarding intervention intensity or specific programme designs. Future research should adopt standardised outcome measures for physical activity and fitness, incorporate longer-term follow-up to assess sustainability, and prioritise inclusion of LMIC populations to enhance global relevance.

## Additional material


Online Supplementary Document
