# Supplementary material for: Interventions for promoting physical activity among adolescents in school settings: systematic review and meta-analysis
Source: J Glob Health. 2026 Mar 27;16:04112. doi: 10.7189/jogh.16.04112 (PMC13022797; doi:10.7189/jogh.16.04112)
Supplement: Online Supplementary Document [file jogh-16-04112-s001.pdf]

Supplement to: Moin M, Farhan Z, Naqvi S, Lassi Z, Das J. Interventions for promoting physical activity among adolescents in school settings: a systematic review and meta-analysis. J Glob Health. 2026;16:04112.

PRISMA 2020 Checklist

Section and Topic	Item #	Checklist item	Location where item is reported
**TITLE**			
Title	1	Identify the report as a systematic review.	Page 1
**ABSTRACT**			
Abstract	2	See the PRISMA 2020 for Abstracts checklist.	Page 2
**INTRODUCTION**			
Rationale	3	Describe the rationale for the review in the context of existing knowledge.	Page 3-4
Objectives	4	Provide an explicit statement of the objective(s) or question(s) the review addresses.	Page 4
**METHODS**			
Eligibility criteria	5	Specify the inclusion and exclusion criteria for the review and how studies were grouped for the syntheses.	Page 5
Information sources	6	Specify all databases, registers, websites, organisations, reference lists and other sources searched or consulted to identify studies. Specify the date when each source was last searched or consulted.	Page 7
Search strategy	7	Present the full search strategies for all databases, registers and websites, including any filters and limits used.	Page 7(S1 File)
Selection process	8	Specify the methods used to decide whether a study met the inclusion criteria of the review, including how many reviewers screened each record and each report retrieved, whether they worked independently, and if applicable, details of automation tools used in the process.	Page 7
Data collection process	9	Specify the methods used to collect data from reports, including how many reviewers collected data from each report, whether they worked independently, any processes for obtaining or confirming data from study investigators, and if applicable, details of automation tools used in the process.	Page 7
Data items	10a	List and define all outcomes for which data were sought. Specify whether all results that were compatible with each outcome domain in each study were sought (e.g. for all measures, time points, analyses), and if not, the methods used to decide which results to collect.	Page 6
	10b	List and define all other variables for which data were sought (e.g. participant and intervention characteristics, funding sources). Describe any assumptions made about any missing or unclear information.	Page 6
Study risk of bias assessment	11	Specify the methods used to assess risk of bias in the included studies, including details of the tool(s) used, how many reviewers assessed each study and whether they worked independently, and if applicable, details of automation tools used in the process.	Page 8(S4)
Effect measures	12	Specify for each outcome the effect measure(s) (e.g. risk ratio, mean difference) used in the synthesis or presentation of results.	Page 8
Synthesis methods	13a	Describe the processes used to decide which studies were eligible for each synthesis (e.g. tabulating the study intervention characteristics and comparing against the planned groups for each synthesis (item #5)).	Page 8
	13b	Describe any methods required to prepare the data for presentation or synthesis, such as handling of missing summary statistics, or data conversions.	Page 8
	13c	Describe any methods used to tabulate or visually display results of individual studies and syntheses.	Page 8
	13d	Describe any methods used to synthesize results and provide a rationale for the choice(s). If meta-analysis was performed, describe the model(s), method(s) to identify the presence and extent of statistical heterogeneity, and software package(s) used.	Page 8
	13e	Describe any methods used to explore possible causes of heterogeneity among study results (e.g. subgroup analysis, meta-regression).	Page 8
	13f	Describe any sensitivity analyses conducted to assess robustness of the synthesized results.	NA
Reporting bias assessment	14	Describe any methods used to assess risk of bias due to missing results in a synthesis (arising from reporting biases).	NA
Certainty	15	Describe any methods used to assess certainty (or confidence) in the body of evidence for an outcome.	Page 8

Section and Topic	Item #	Checklist item	Location where item is reported
assessment			
**RESULTS**			
Study selection	16a	Describe the results of the search and selection process, from the number of records identified in the search to the number of studies included in the review, ideally using a flow diagram.	Fig 1
	16b	Cite studies that might appear to meet the inclusion criteria, but which were excluded, and explain why they were excluded.	Fig 1
Study characteristics	17	Cite each included study and present its characteristics.	Page 10 (S2)
Risk of bias in studies	18	Present assessments of risk of bias for each included study.	(S4)
Results of individual studies	19	For all outcomes, present, for each study: (a) summary statistics for each group (where appropriate) and (b) an effect estimate and its precision (e.g. confidence/credible interval), ideally using structured tables or plots.	Page 11
Results of syntheses	20a	For each synthesis, briefly summarise the characteristics and risk of bias among contributing studies.	Page 11
	20b	Present results of all statistical syntheses conducted. If meta-analysis was done, present for each the summary estimate and its precision (e.g. confidence/credible interval) and measures of statistical heterogeneity. If comparing groups, describe the direction of the effect.	Page 11-13
	20c	Present results of all investigations of possible causes of heterogeneity among study results.	Page 11-13
	20d	Present results of all sensitivity analyses conducted to assess the robustness of the synthesized results.	NA
Reporting biases	21	Present assessments of risk of bias due to missing results (arising from reporting biases) for each synthesis assessed.	NA
Certainty of evidence	22	Present assessments of certainty (or confidence) in the body of evidence for each outcome assessed.	Page 11
**DISCUSSION**			
Discussion	23a	Provide a general interpretation of the results in the context of other evidence.	Page 18-20
	23b	Discuss any limitations of the evidence included in the review.	Page 21
	23c	Discuss any limitations of the review processes used.	Page 21
	23d	Discuss implications of the results for practice, policy, and future research.	Page21-22
**OTHER INFORMATION**			
Registration and protocol	24a	Provide registration information for the review, including register name and registration number, or state that the review was not registered.	NA
	24b	Indicate where the review protocol can be accessed, or state that a protocol was not prepared.	S6
	24c	Describe and explain any amendments to information provided at registration or in the protocol.	NA
Support	25	Describe sources of financial or non-financial support for the review, and the role of the funders or sponsors in the review.	NA
Competing interests	26	Declare any competing interests of review authors.	NA
Availability of data, code and other materials	27	Report which of the following are publicly available and where they can be found: template data collection forms; data extracted from included studies; data used for all analyses; analytic code; any other materials used in the review.	S1, S2, S3, S4

From: Page MJ, McKenzie JE, Bossuyt PM, Boutron I, Hoffmann TC, Mulrow CD, et al. The PRISMA 2020 statement: an updated guideline for reporting systematic reviews. BMJ 2021;372:n71. doi:

PRISMA 2020 Checklist

10.1136/bmj.n71. This work is licensed under CC BY 4.0. To view a copy of this license, visit https://creativecommons.org/licenses/by/4.0/

S1 File. Search Strategy

PubMed

("Adolescent" [MeSH] OR "adolescent health"[MeSH Major Topic] OR “teenager” OR “young adult”)

AND

(“Physical Activity” [MeSH] OR “physical fitness” [MeSH Terms] OR "physical education training"[MeSH Terms] OR "youth sports” [MeSH Terms] OR “youth education"[MeSH Terms] OR "physical phenomena/education"[MeSH Major Topic] OR "Exercise" OR "physical training" OR "Fitness" OR "sedentary lifestyle" OR “active lifestyle” OR "PE" OR “accelerometer” OR "Sport" OR "Walk" OR “aerobic” OR “gymnastics” OR “Gym” OR “Yoga” OR “Pilates” OR “High intensity training” OR “Running” OR “Cycling” OR “Pilates” OR “Dancing” OR “Soccer” OR “Basketball” OR “Football” OR “Cricket” OR “Tennis” OR “Golf” OR “Martial arts” OR “Swimming” OR “Jogging” OR “Rowing” OR “Hiking” OR “Elliptical training” OR “Jump rope” OR “Weight lifting” OR “Powerlifting” OR “Kettlebell” OR “Push-ups” OR “Pull-ups” OR “Resistance band exercise” OR “Planks” OR “CrossFit” OR “Stretching” OR “Tai chi” OR “Boot camps” OR “Sprints” OR “Tabata” OR “Water aerobics” OR “Rock climbing” OR “Volleyball” OR “Kickboxing”)

AND

("schools"[MeSH Terms] OR “Primary School” OR “college” OR "Secondary School" OR "High School" OR "school-based" OR “education institute” OR “academic institution” OR “curriculum” OR “classroom”)

2000 onward, Humans, English, Clinical Trials, RCTs, Systematic review

Results:3614

Embase

('adolescent'/exp OR 'adolescent health'/mj OR 'teenager' OR 'young adult')

AND

('physical activity'/exp OR 'physical fitness'/exp OR 'physical education training'/exp OR 'youth sports'/exp OR 'youth education'/exp OR 'exercise'/exp OR 'physical training' OR 'fitness' OR 'sedentary lifestyle' OR 'active lifestyle' OR 'PE' OR 'accelerometer' OR 'sport'/exp OR 'walk' OR 'aerobic' OR 'gymnastics' OR 'gym' OR 'yoga' OR 'pilates' OR 'high intensity training' OR 'running' OR 'cycling' OR 'pilates' OR 'dancing' OR 'soccer' OR 'basketball' OR 'football' OR 'cricket' OR 'tennis' OR

'golf' OR 'martial arts' OR 'swimming' OR 'jogging' OR 'rowing' OR 'hiking' OR 'elliptical training' OR 'jump rope' OR 'weight lifting' OR 'powerlifting' OR 'kettlebell' OR 'push-ups' OR 'pull-ups' OR 'resistance band exercise' OR 'planks' OR 'crossfit' OR 'stretching' OR 'tai chi' OR 'boot camps' OR 'sprints' OR 'tabata' OR 'water aerobics' OR 'rock climbing' OR 'volleyball' OR 'kickboxing')

AND

('schools'/exp OR 'primary school' OR 'college' OR 'secondary school' OR 'high school' OR 'school-based' OR 'education institute' OR 'academic institution' OR 'curriculum' OR 'classroom')

1 (('adolescent' or 'adolescent health' or 'teenager' or 'young adult') and ('physical activity' or 'physical fitness' or 'physical education training' or 'youth sports' or 'youth education' or 'exercise' or 'physical training' or 'fitness' or 'sedentary lifestyle' or 'active lifestyle' or 'PE' or 'accelerometer' or 'sport' or 'walk' or 'aerobic' or 'gymnastics' or 'gym' or 'yoga' or 'pilates' or 'high intensity training' or 'running' or 'cycling' or 'pilates' or 'dancing' or 'soccer' or 'basketball' or 'football' or 'cricket' or 'tennis' or 'golf' or 'martial arts' or 'swimming' or 'jogging' or 'rowing' or 'hiking' or 'elliptical training' or 'jump rope' or 'weight lifting' or 'powerlifting' or 'kettlebell' or 'push-ups' or 'pull-ups' or 'resistance band exercise' or 'planks' or 'crossfit' or 'stretching' or 'tai chi' or 'boot camps' or 'sprints' or 'tabata' or 'water aerobics' or 'rock climbing' or 'volleyball' or 'kickboxing') and ('schools' or 'primary school' or 'college' or 'secondary school' or 'high school' or 'school-based' or 'education institute' or 'academic institution' or 'curriculum' or 'classroom')).mp. [mp=title, abstract, heading word, drug trade name, original title, device manufacturer, drug manufacturer, device trade name, keyword heading word, floating subheading word, candidate term word] 18234

2 limit 1 to ("remove medline records" and yr="2000 -Current") 5437

3 limit 2 to ("remove medline records" and yr="2000 -Current") 5437

CINAHL

Database Applied: CINAHL Complete

Limiters : Publication Date: 20000101-20241231; English Language; Human

("Adolescent" OR "adolescent health" OR teenager OR "young adult")

AND

("Physical Activity" OR "physical fitness" OR "physical education training" OR "youth sports" OR "youth education" OR "physical phenomena/education" OR Exercise OR "physical training" OR Fitness OR "sedentary lifestyle" OR "active lifestyle" OR PE OR accelerometer OR Sport OR Walk OR aerobic OR gymnastics OR Gym OR Yoga OR Pilates OR "High intensity training" OR Running OR Cycling OR Dancing OR Soccer OR Basketball OR Football OR Cricket OR Tennis OR Golf OR "Martial arts" OR Swimming OR Jogging OR Rowing OR Hiking OR "Elliptical training" OR "Jump rope" OR "Weight lifting" OR Powerlifting OR Kettlebell OR "Push-ups" OR "Pull-ups" OR "Resistance band exercise" OR Planks OR CrossFit OR Stretching OR "Tai chi" OR "Boot camps" OR Sprints OR Tabata OR "Water aerobics" OR "Rock climbing" OR Volleyball OR Kickboxing)

AND

("schools" OR "Primary School" OR college OR "Secondary School" OR "High School" OR "school-based" OR "education institute" OR "academic institution" OR curriculum OR classroom)

Results:5026

SCOPUS

(TITLE-ABS-KEY (exercise OR "physical education" OR "physical activity" OR "physical fitness" OR sedentary OR sport* OR walk* OR danc*) AND TITLE-ABS-KEY (adolescen*) AND TITLE-ABS-KEY (school*)) AND PUBYEAR > 1999 AND PUBYEAR < 2025 AND (LIMIT-TO (EXACTKEYWORD , "Human")) AND (LIMIT-TO (LANGUAGE , "English")) AND (EXCLUDE (SUBJAREA , "BIOC") OR EXCLUDE (SUBJAREA , "PSYC") OR EXCLUDE (SUBJAREA , "NEUR") OR EXCLUDE (SUBJAREA , "AGRI") OR EXCLUDE (SUBJAREA , "ARTS") OR EXCLUDE (SUBJAREA , "IMMU") OR EXCLUDE (SUBJAREA , "PHAR") OR EXCLUDE (SUBJAREA , "ENGI") OR EXCLUDE (SUBJAREA , "DENT") OR EXCLUDE (SUBJAREA , "COMP") OR EXCLUDE (SUBJAREA , "CHEM") OR EXCLUDE (SUBJAREA , "CENG") OR EXCLUDE (SUBJAREA , "BUSI") OR EXCLUDE (SUBJAREA , "MATH") OR EXCLUDE (SUBJAREA , "VETE") OR EXCLUDE (SUBJAREA , "PHYS") OR EXCLUDE (SUBJAREA , "ECON") OR EXCLUDE (SUBJAREA , "EART") OR EXCLUDE (SUBJAREA , "MATE"))

Results: 19608

Cochrane

Search Name: Search strategy 1

Last Saved: 24/06/2024 06:32:07

Result: 770

ID Search

#1 MeSH descriptor: [Adolescent] explode all trees

#2 ("adolescent"):ti,ab,kw OR ("adolescent health"):ti,ab,kw OR ("teen-ager"):ti,ab,kw OR ("young adult"):ti,ab,kw (Word variations have been searched)

#3 #1 OR #2

#4 MeSH descriptor: [Exercise] explode all trees

#5 (“Physical Activity” OR “physical fitness” OR "physical education training" OR "youth sports” OR “youth education" OR "physical education" OR "Exercise" OR "physical training" OR "Fitness" OR "sedentary lifestyle" OR “active lifestyle” OR "PE" OR “accelerometer” OR "Sport" OR "Walk" OR “aerobic” OR “gymnastics” OR “Gym” OR “Yoga” OR “Pilates” OR “High intensity training” OR “Running” OR “Cycling” OR “Pilates” OR “Dancing” OR “Soccer” OR “Basketball” OR “Football” OR “Cricket” OR “Tennis” OR “Golf” OR “Martial arts” OR “Swimming” OR “Jogging” OR “Rowing” OR “Hiking” OR “Elliptical training” OR “Jump rope” OR “Weight lifting” OR “Powerlifting” OR “Kettlebell” OR “Push-ups” OR “Pull-ups” OR “Resistance band exercise” OR “Planks” OR “CrossFit” OR “Stretching” OR “Tai chi” OR “Boot camps” OR “Sprints” OR “Tabata” OR “Water aerobics” OR “Rock climbing” OR “Volleyball” OR “Kickboxing”):ti,ab,kw (Word variations have been searched)

#6 #4 OR #5

#7 MeSH descriptor: [Schools] explode all trees

#8 #3 AND #6 AND #7

S2 File. Study Characteristics

Author	Publication year	Study details				Participants			Intervention	Comparison	Others	
		Study design	Country	Setting	Duration of study	Number	Sex	Follow-up Duration			Study limitations	Trial funding source
Jago (1)	2011	Cluster randomized controlled trial	United States	42 middle schools across the US	2.5 years	4063	52.4% female	2.5 years	HEALTHY intervention, a multicomponent intervention focusing on: 1. Improved school food environment 2. Physical activity and eating education	Control group with standard school practices (recruitment and data collection only, no intervention)	No differences in Met-S, fitness, or physical activity were observed. Measurement tools such as the self-reported MVPA may have	National Institute of Diabetes and Digestive and Kidney Diseases/National Institutes of Health

									al sessions 3. Social marketing 4. Revised physical education curriculum to promote MVPA		affected results.	
Lubans (2)	2018	Cluster randomized controlled trial	Australia	14 secondary schools in low socioeconomic areas of Western	14-15 months	1173	(51.9% male, 48.1% female)	14–15 months after baseline	Activity and Motivation in Physical EDucation (AMPED) intervention	Control group schools with standard PE practices.	First, we did not objectively measure students_ engagement in mathematics using classroom observat	This project was funded by the Australian Research Council (ARC) Discover Project Grant (DP130104659

				Sydney							ions. Previous studies have demonstrated that students spend more time engaged in the classroom after they have been physically active. Second, failure to assess maturity status may be	

											considered a study limitation. Third, we were not able to obtain measures of the mediators at the same time as the pretest assessments of mathematics were obtained (the study started	

											in grade 8, but mathematics performance was assessed in grade 7). Our failure to assess aerobic fitness and motor competence are also study limitations.<br><br>Finally, this study did not include	

											measures of cognitive function (working memory, inhibition, or task flexibility)	
Webber (3)	2008	Group randomized controlled trial	United States	36 middle schools	2 years	3504	All female	2 years	The TAAG intervention25 incorporated operant learning theory,26 social cognitive theory,27 organizational change	Delayed intervention for control group		This study was funded by the following cooperative agreements from the National Heart, Lung and Blood Institute: U01 HL066855 (Tulane University);

									theory,28 and the diffusion of innovation model29 in a social-ecologic framework.30 –34 Intervention activities based on extensive formative research were targeted to create (1) environmental and organizational changes supportiv			U01HL066845 (University of Minnesota); U01HL066852 (University of South Carolina); U01HL066853 (University of North Carolina at Chapel Hill); U01HL066856 (San Diego State University); U01HL066857 (University of Maryland); U01HL066858 (University of Arizona).

									e of physical activity and (2) cues, messages, and incentives to be more physically active. Specifically, the intervention was designed to establish more opportunities, improve social support and norms, and increase			

									self-efficacy, outcome expectations, and behavioral skills to foster greater MVPA.35 – 40 Girls were the focus of the intervention; however, health and physical education classes were part of the usual school curriculum and most included			

									boys as well.			
Corder(4)	2016	Feasibility study and pilot cluster- randomised controlled trial (C	United Kingdom	Three schools (two intervention, one control)	8 weeks	460	46.6% female	8 weeks	8-week intervention (2013) involved: classes choosing weekly activities encouraged by mentors (older adolescents) and in-class peer leaders. Students gain points for trying	Standard physical education without GoActive intervention	Limited number of clusters; no cost-effectiveness data collected	Funding for this study and the work of all authors was supported, wholly or in part, by the Centre for Diet and Activity Research (CEDAR), a UKCRC Public Health Research Centre of Excellence (RES-590-28-0002).

		RCT)							activities which are entered into an intramural competition.			Funding from the British Heart Foundation, Department of Health, Economic and Social Research Council, Medical Research Council, and the Wellcome Trust, under the auspices of the UK Clinical Research Collaboration, is gratefully acknowledged. The work of KC, HEB and EMFvS was supported by the Medical

												Research Council (MC_UU_12 015/7).
Hankon en (5)	201 7	Clu ster - ran do mis ed con trol led trial	Fin lan d	voca tiona l scho ol	6 mon ths	43	85 % fe ma le	baselin e (T1), mid-interve ntion (T2), post-interve ntion (T3), 6 month s (T4).	The interventi on consisted of (1) a 6-h group-based interventi on for students, (2) two 2-h training workshop s to reduce their students' sitting in	Contr ol group receiv ed usual health educat ion curric ulum and a leaflet on PA recom menda tions	Limitati ons include a focus on one school only, but resource s did not allow for a larger study, and in fact,	The study was supported by Ministry of Social Affairs and Health, funding number 201310238, and the Ministry of Education and Culture, funding number 34/626/2012. The first author was

									class for teachers, and (3) provision of light PA equipment in classrooms.		key intervention components act at the individual and interpersonal rather than the school level. Second, due to the novelty, the self-report measure for BCTs was not robustly validated.	supported by by the Academy of Finland (Academy Research Fellowship). The funding bodies played no role in the writing of this manuscript or the decision to submit it for publication.

											However, criterion validity is indicated by the relatively high correlations with objectively measured PA. Furthermore, it was more precise than previously reported enactment scales using	

											dichotomous responses only (e.g. [13]), measuring also the use of BCTs in control participants. Third, the process may have benefited from a priori set criteria forindicators of acceptability and	

											feasibility	
Lonsdale (6)	2013	Cluster randomized controlled trial	Sydney, Australia	Independent and Catholic secondary schools		288	50.4% male	Post-intervention (around 10 days after baseline)	Three motivational strategies: (1) Relevance (n=60), (2) Providing Choice (n=77), (3) Free Choice (n=71). PE teachers trained for 20 minutes	Usual practice PE group	Short-term study; generalizability limited by modest sample size and school selection bias.	Not reported

									before delivering the interventions.			
Jago et al.(7)	2015	School-based cluster randomized controlled trial	UK (United Kingdom)	Secondary schools in Bath, Bristol, and North Somerset	12 months	571	Female	Data collected at baseline (T0), during the intervention (T1), and at one-year follow-up (T2)	Up to 40 sessions of 75 minutes after-school dance classes delivered twice weekly for 20 weeks. Delivered by dance instructors in school facilities.	Control group (did not receive dance intervention)	Low adherence (only one-third of participants attended two-thirds of sessions); intensity of dance sessions might have been insufficient to significantly	National Institute for Health Research Public Health Research Programme, with additional support from Bristol Primary Care Trust and Local Councils

											impact MVPA	
Solberg (8)	2021	Cluster randomized controlled trial (RCT)	Norway	Lower secondary schools in Norway	9 months (September 2017 to June 2018)	2084	50% female, 50% male	Baseline, during intervention, and follow-up after intervention	The PAL intervention focused on increasing the students' PA levels and consisted of three components of at least moderate intensity: (1) additional lesson of PE per week (60	Control group (no additional PA beyond mandatory curriculum)	Measurement error in aerobic fitness assessment, lack of non-active control group, potential for confounding due to unmeasured variables	Norwegian Directorate for Education and Training; additional funding from the Medical Research Council

									min); (2) 30 min/week lesson of physically active learning where physical activities were integrated in regular subjects; and (3) 30 min/week lesson of PA that included a variety of enjoyable activities. In contrast, the DWBH interventi on's focus was to			

									promote friendship through PA and consisted of two components: (1) 60 min of PE called the ‘Don’t worry’ lesson (DW) and (2) a 60 min ‘Be happy’ lesson (BH). Based on PA interest, students formed groups of 3–8 students and chose one			

									activity that was performed throughout the intervention period.			
Lubans (9)	2012	Group randomized controlled trial (RCT)	Australia	Low socio-economic status (SES) secondary schools in the Hunter Region,	6 months (June to December 2009)	100	Male	Baseline, 3 months, 6 months	PALs program (Physical Activity Leaders): 10 enhanced school sports sessions, interactive seminars, lunchtime activities, self-monitoring with pedometers	Wait-list control group	No significant increase in physical activity; only boys were studied; underpowered to detect changes in activity	Supported by the Hunter Medical Research Institute and the Rotary Club of Newcastle Enterprise

				New South Wales								
Bergh (10)	2012	Group randomized controlled trial	Norway	Schools located in the Eastern part of Norway	20 months	700	60 % girls in the intervention group	20 months	Active commuting campaigns, sports equipment for recess, posters, classroom lessons, activity breaks, PE teacher training, fact sheets for parents		accelerometers assessed PA are not able to capture water activities, record cycling, upper body movements, carrying a load correctly or detect context specific	orwegian Research Council, Throne Holst Nutrition Research Foundation, University of Oslo, Norwegian School of Sport Sciences, European Commission

											changes in PA, and using logs in addition to accelerometers could have compensated for these drawbacks [45,46]. The larger than expected drop-out [19], could have caused a loss of	

											power and may have influenced the results, especially among the overweight. There exists several sets of BMI reference data that are intended to define childhood overweight [47]. Therefo	

											re, we cannot rule out the possibility that the results for the moderated mediation of weight status might have been slightly different applying for example the cut off values for the World	

											Health Organizations’ growth curves or the Center for Disease Control. Still, within the HEIA study we choose to use the IOTF’s criteria for defining overweight/obesity to allow for compari	

											ng prevalence data across nations [47,48]. In addition, those lost to the PI assessment were more likely to be boys, but in the analyses this was compensated for by adjusting for gender. Among those lost to	

											PI, higher values for mcpm were found in the control group compared to the intervention group, but this wasthe case in the study sample also (Table 2) and adjusted for the analysis	

Guijarro-Romero (11)	2020	Cluster-randomized controlled trial (RCT)	Spain	Two state high schools in Ciudad-Real, Castilla-La Mancha Region, Spain	9 weeks (16 sessions in total)	126	53 boys 73 girls	Post-intervention (end of 9 weeks)	**Intervention Groups: Traditional group (TG) received traditional fitness-based PE; Intermittent group (IG) received intermittent PE focused on fitness + sports**	**Control Group (CG): Participated in regular PE with no special focus on physical activity intensity**	Gender imbalance, no measurement of technical-tactical learning, short intervention duration	Spanish Ministry of Science, Innovation and Universities
Bonsergent(12)	2013	Cluster randomized con	France (Region of Lor	24 public high schools in	2 years	3538	57.6% girl	2 years	**Education strategy: Nutritional and physical activity**	No strategy (control schools	Selection bias due to non-completers, lack of waist	Funded by several public and private organizations, including ARH Lorraine,

		trol led trial	rai ne)	the regio n of Lorr aine					**education , group work, and PRALIM AP parties. Environ ment strategy: Improve d availabili ty of healthy foods and opportun ities for physical activity. Screenin g and care strategy: Identifica tion of overweig ht/obesity by school**	receiv ed no interv ention)	circumf erence data, small effect size despite signific ance	Fondation Wyeth, and Ministry of Higher Education and Research

									**nurses and tailored care sessions if necessary.**			
Telford (13)	2016	Cluster randomized controlled trial (RCT)	Australia (Australian Capital Territory)	29 public primary schools	4 years (2006-2009)	853	both	4 years (measured from baseline in Grade 2 to Grade 6)	**Intervention Group: 2 × 45-min PE lessons per week (total of 90 min/week) taught by specialist PE teachers;**	**Control Group: Received regular PE lessons taught by generalist classroom teachers**	The amount of PA in the Intervention classes may have been underestimated. The intervention involved activities that may not have	Commonwealth Education Trust

											been accurately detected by pedometers or accelerometers; activities borrowed from yoga, involving balance and isometric muscular contraction. These types of activitie	

											s were often perform ed while sitting or stationa ry and may have been classifie d as sedentar y type activity during PE lessons using SOFIT.s tudy may have been improve d,	

											and stronger inferences drawn on intervention effects on the intensity of PA had we been able to use accelerometers from the beginning of the study Another limitation was the	

											difficulty of assessing the frequency of PE classes in the Control schools.	
Toftager (14)	2014	Cluster randomized controlled trial (RCT)	Denmark	14 public schools in the Region of Southern Denmark	2 years (Baseline in 2010, Follow-up in 2012)	1348	48.4% (GIRLS)	2 years	The physical environment changes required included the following components: 1) upgradeexisting outdoor areas at the school for PA,	Control group schools did not receive any of the intervention components	Low effect size on overall PA, different degrees of implementation across schools, limited financial resource	Funded by TrygFonden as part of the Centre for Intervention Research in Health Promotion and Disease Prevention

									including unfixedequipment, 2) develop and build playgrounds specially designed for adolescents: play spots, 3) improve safety for active transport to and from school, 4) establish an after school fitness program. Theorganizational environm		s affected full intervention	

									ent changes included: 5) formulate and implement school PA policy, 6) educate teachers as ‘‘kick-starters’’, who facilitate and motivate PA during recess, 7) establish school play patrol: older students were trained to initiate			

									play and games for minors during school recess, 8) mandatory outdoor recess and/or free access to gym/sports hall, 9) school traffic patrol: older students helped minors cross the streets near the school, 10)educate and train students			

									in safe cycling, and finally 11) school project/theme week once a year focusing on learning about and doing PA during school lessons			
Tarp (15)	2016	Cluster randomized controlled trial	Denmark	16 Danish schools (7 intervention and 7	20 weeks	632	both	Baseline, post-intervention (20 weeks)	**Intervention Group: Target of 60 min/day of physical activity during school**	Control group received normal school activities witho	The main limitation of the study is the poor intervention fidelity schools were	Funded by the Danish Ministry of Education

		(R CT)		contr ol scho ols)					**time using active learning, structure d recess, and physical activity homewor k**	ut a specifi c physic al activit y interv ention	recruite d through a primary school physical activity and health project, which could be consider ed a limitatio n, as the include d schools may have a physical activity awarene ss, which differs from	

											other public schools in Denmark.<br><br>Possibly, the degree of changes to implement at intervention schools may have been reduced. Also, the differences observed at baseline	

											indicate that the randomization process did not result in completely comparable groups. As the number of clusters was limited (14 schools) some differences after randomization are not unexpected.	

Petrušic (16)	2022	Cluster randomized controlled trial (RCT)	Slovenia	1 public school	12 weeks	59	girls	Baseline and post-intervention (12 weeks)	**Intervention Group: Game-based training in addition to regular physical education classes, 2 sessions per week (40 min each) for 12 weeks**	Control group received only regular physical education without any additional intervention	used only adolescent girls in the current study, did not monitor students' overall physical activity during the intervention period or food intake, which could affect the overall results	This research was funded by Slovenian Research Agency (postdoctoral research project Z3-3212) and ARRS Research and Infrastructure Programme number P3-0323 (Renal Diseases and Renal Replacement Therapy).

Zhang(17)	2020	Randomized Controlled Trial (RCT)	China	Junior middle school in Beijing	11 weeks	51	47.1% MALE	Baseline, post-intervention (8 weeks)	**Intervention Group: 8-week program based on the extended Theory of Planned Behavior, including indoor lectures and outdoor physical activities**	Control group received no intervention, only pre- and post-tests	small sample size, characteristics of the school selected. We only chose adolescents from one school in Beijing, and it was not representative enough of the whole Chinese adolescent populati	This work was supported by the National Social Science Fund of China, funded by the Chinese government (16BTY065), and Leading Talents of Independent Research Program, funded by Tsinghua University (No. 2016THZWLJ12).

											on. third limitation is related to the intervention period being too small.	
Sebire (18)	2018	Cluster randomized controlled trial (RCT)	UK (England)	6 Secondary schools (4 intervention, 2 control)	10 weeks (intervention period), with follow-up at the start of Yea	427	girls	Baseline (Time 0), Post-intervention (Time 1, after 10 weeks), Follow-up (Time 2, start of	**The intervention involved training Year 8 girls (out of school for two consecutive days, plus one top-up day 5 weeks later), who were identified**	Control group received usual practice without intervention	Small sample size, lack of significant ethnic diversity, high levels of missing accelerometer data at follow-up (Time 2)	National Institute for Health Research (NIHR), Public Health Research Programme

					r 9 (Time 2)			Year 9)	**by their peers as influential, to provide informal support to their friends to increase their physical activity**			
Eather (19)	2015	Cluster randomized controlled trial (RCT)	Australia	Secondary school in the Hunter Region, NSW	8 weeks	96	51.5% female	Baseline (T0) and post-intervention (T1) at 8 weeks	The CrossFit Teens™ fitness programme used in this study was designed specifically for adolescents by the two experienced	Control group received normal PE lessons and 60-minute weekly sport	Short intervention duration, small sample size, no long-term follow-up	Funded by the University of Newcastle, Australia

									CrossFit ™ instructors who delivered the programme and was based on a “group training” atmosphere and a game setting, to engage adolescent participants (Glassman, 2010). It incorporated combinations of core strength	lessons		

									exercises that adolescents could perform safely (e.g. squat jumps, push-ups, deadlifts), utilised the outdoor facilities and basic equipment available at the participating school (e.g. school bags, medicine balls, broom sticks, basketball court) and			

									targeted improvement in health-related fitness.			
Lubans (20)	2011	Randomized Controlled Trial (RCT)	Australia	Four disadvantaged secondary schools in New South Wales, Australia	6 months (June to December 2009)	100	boys	Baseline, 3 months, 6 months	The PALs program was a multi-component school-based intervention and included school sport sessions, interactive seminars, lunch-time activities, physical activity and	Control group received no intervention, usual physical education	Small sample size, short follow-up period, no assessment of long-term effects	Supported by Hunter Medical Research Institute and the Rotary Club of Newcastle Enterprise

									nutrition handbooks, leadership sessions and pedometers for self-monitoring.			
Seger (21)	2022	Cluster randomized controlled trial (RCT)	Sweden	Three schools outside Stockholm	2 years	148	boys	Baseline (Autumn Year 7), Year 1 (Spring Year 7), Year 2 (Spring Year 8)	In the two-year project, 122 pupils aged 13–14 years from three schools constituted an aerobic group with 30 min extra exercise sessions (≥70% maximal heart rate)	Control group had only regular PE lessons (no extra aerobic sessions)	Limited improvement in fitness for girls, no long-term follow-up after Year 8, focus only on aerobic fitness and muscular strength	Supported by Swedish government education and research initiative

									twice weekly			
Kennedy (22)	2018	Cluster Randomized Controlled Trial (RCT)	Australia	16 secondary schools across the Hunter, Central Coast, and Sydney regions of NSW	10 weeks	607	(50.1% girls	Baseline, 6 months, 12 months	Teachers were trained to deliver the intervention, which included the following: (i) an interactive student seminar; (ii) a structured physical activity program, focused on RT; (iii) lunchtime fitness	Control group received regular PE lessons without intervention	No significant effect on lower body fitness, potential bias due to self-reported data, short intervention period	Supported by the Australian Research Council and NSW Department of Education

									sessions; and (iv) Web-based smartphone apps.			
Miller (23)	2015	Cluster randomized controlled trial (RCT)	Australia	7 primary schools, Hunter Region, NSW, Australia	(8 week intervention followed by an 8 week period for the control group	168		Baseline (T0), Post-intervention (T1, 8 weeks)	**Intervention Group: PLUNGE program using Game-Centered Curriculum, in-class mentoring, and PE lessons with teacher professional development**	Control group followed the regular PE curriculum	Small sample size, short intervention duration, lack of long-term follow-up	School of Education, University of Newcastle

Parrish(24)	2018	Randomized Controlled Trial (RCT)	Australia	Four private high schools in New South Wales, Australia	5 months	88	(50% male, 50% female	Baseline, post-intervention (5 months)	**Intervention Group: Reduced sitting time through use of standing desks, structured physical activity modules, and standing strategies**	Control group continued with usual school curriculum	Small sample size, low intervention fidelity, lack of long-term follow-up	Funded by the National Heart Foundation of Australia
Young (25)	2006	Randomized Controlled Trial (RCT)	United States (Baltimore)	Baltimore magnet high school	8 months	221	girls	Baseline (T0), post-intervention (T1, 8 months)	**Intervention Group: Life skills–oriented PE curriculum designed to**	Standard PE class (team sports, individual sports, skill develo	Self-reported data, single high school setting, limited generalizability	Funded by National Institutes of Health (NIH)

									**promote physical activity, including goal setting, problem-solving, and support strategies**	pment)		
Costigan (26)	2015	Pilot Randomized Controlled Trial (RCT)	Australia	One secondary school in Newcastle, Australia	8 weeks	65	**(45 males, 20 females**	Baseline, post-intervention (8 weeks)	Participants in the intervention groups participated in three HIIT sessions/ week for eight weeks and all sessions were conducted inside the school	Regular PE and usual lunchtime activities	Small sample size, single school, short intervention period	Hunter Medical Research Institute

									hall. Two HIIT sessions/ week were delivered in scheduled PE lessons, with a third session delivered at lunchtime. The focus of each of the three programs included:<br>i. AEP: Participants completed HIIT sessions primarily			

									involving gross motor cardiorespiratory exercises requiring minimal equipment (e.g., shuttle runs, jumping jacks, skipping);<br>ii. RAP: Participants completed HIIT sessions that included a combination of cardiorespiratory and body			

									weight resistance training exercises that required minimal equipment (e.g., body weight squats, push-ups, hovers)			
Weigensberg (27)	2021	Randomized Controlled Trial (RCT)	United States	Four urban high schools in Los Angeles, California	12 weeks	232	66% female, 94% Latinx	Baseline (T0), post-intervention (T1, 12 weeks)	The four intervention arms were: (1) nonintervention "Control" (C); (2) "Lifestyle education" (LS), consisting of 12 weeks of	Control group (C) received no intervention	The primary limitation of the study was the poor participant adherence, thereby limiting the	Funded by National Institutes of Health (NIH)

									twice weekly 75-min sessions of didactic and experiential education relating to healthy eating (one session per week) and physical activity (one session per week) practices consistent with consensus pediatric recommendations,1		degree to which conclusions can be made regarding the effectiveness of GI in this study. Generalizability of results is uncertain beyond the predominantly Latinx, lower socioeconomic, adolesc	

									2–15 in particular emphasizing modification in quality of carbohydrate intake8,16,17 and key concepts of “Intuitive Eating,”18–20 a nondieting approach to healthy eating; (3) “SRGI”, which received the same lifestyle education plus an		ent study population. Finally, although physical activity was objectively measured using wrist-worn accelerometers, interpreting the results by the absolute amount may be misleading, given the	

									additional once weekly 75-min group SRGI session for 10 sessions; (4) “LBGI”, which received the same lifestyle education, plus four weekly 75-min group SRGI sessions, followed by six weekly sessions of GI designed to		challenges related to the accuracy of classifying data from wrist-worn accelerometer collected in free-living environment.44 Therefore, the current study interpreted the findings in terms of the magnitu	

									motivate improved eating and activity behaviors.		de of change before and after the intervention.	
Zarrett(28)	2021	Randomized Controlled Trial (RCT)	United States	Afterschool programs in 6 middle schools	10 weeks	167	56% female,	Baseline (T0), post-intervention (T1, 12 weeks)	The primary components of the intervention included small group "Get-to-Know-You" sessions and the implementation of novel socially-oriented	Control group received usual afterschool program	The study design included only participants who wore accelerometers at baseline, a subset of the total sample particip	Funded by the National Institutes of Health (NIH)

									PA sessions		ating in the intervention Relatedly, Connect through PLAY was implemented in ASPs serving underserved youth in the southeastern United StatesThis study's randomized-controlled research	

											design helps to control for these “accelerometer reactivity” efects, however, future research can also include a longer “run-in” baseline period that allows for additional non-recorded wear	

											days so that participants become accustomed to the device before data is collected	
Kurnaz (29)	2024	Randomized Controlled Trial (RCT)	Turkey, Portugal	One educational institution in Turkey	10 weeks	50	males and females	Baseline and post-intervention (10 weeks)	Intervention Group: Play-based after-school physical activity program (twice a week for 40 min); Control Group: Standard physical	Control group received standard PE lessons	the distribution between groups was not balanced regarding sex, and sex diferences may have infuenced our	Not disclosed

									education classes		results, especially regarding CMJ as previously indicated in the literature53,54. Te disparity in participant representation between boys and girls may introduce bias and afect the validity	

											of sex-related conclusions drawn from the research. Tis imbalance may not accurately refect the motor performance of all sex groups, potentially limiting the generalizability of the fndings. Future research	

											should aim for a more balanced representation of sexes to ensure more comprehensive and accurate conclusions regarding sex-related phenomena. Finally, the additional 40 min of physical exercise provide	

											d to the experimental group can infuence the results and need to be more investigated, compared to other forms of intervention.	
Andrade (30)	2014	Cluster-Randomized Controlled	Ecuador	Schools in urban Cuenca, Ecuador	28 months	1440	both, female % IG 66.4 fe	Baseline and post-intervention (28 months)	A school-based health promotion intervention on “ACTIVITAL” that aimed at improving	Standard Ecuadorian PE curriculum (80 minut	High attrition rate in accelerometer data, no long-term follow-up,	Universidad de Cuenca, VLIR-UOS, and Nutrition Third World

		Trial (RCT)					male % CG 59.3		diet and physical activity. ACTIVITAL was developed using participatory approaches.Educational sessions, environmental modifications, workshops for parents, interactive sessions with local athletes	es/week)	variation in results across school pairs	
White (31)	2022	Group Randomiz	Australia	One secondary scho	20 weeks	89	female	6 months (post-interve	The 20-week multi-component HWBG	Wait-list control group	Small sample size, single school	No specific funding reported

		ed Controlled Trial (RCT)		ol in New South Wales				ntion follow-up)	program, guided by Self-Determination Theory and Acceptance and Commitment Therapy, was designed and delivered by a member of the research team	(received no intervention during the study but completed an elective course)	setting, teacher who was a researcher also delivering the program, use of pedometers instead of accelerometers for physical activity measurement	
Shimon (32)	2009	Randomized Controlled Tri	United States (Northw	Junior high school, 7th and 8th-	5 weeks	159	101 girls. 93 boys	Baseline, Week 1, Week 2, Week 3,	Self-Regulation Group: Non-sealed pedometers, recording	Sealed pedometers (control group), no feedba	High attrition rate, particularly among girls, self-	Idaho Orthopaedic Institute at Saint Alphonsus and Boise State University

		al (RCT)	est region)	grade PE classes				Week 4	and plotting daily step counts, and goal-setting strategies; Open Group: Non-sealed pedometers, no plotting	ck on step counts	reporting biases due to pedometer viewability	
Simon(33)	2014	Cluster-Randomized Controlled Trial	France	8 middle schools in the Bas-Rhin region	4-year intervention with a 30-month post-trial foll	732	males at intervention 41.50% at control 49.	Baseline (T0), end of intervention (4 years), post-intervention (30 months after trial)	Intervention students followed a program that began with the first school year and lasted until the end of the fourth school	Standard PE curriculum without additional intervention	Self-reported LPA and TV time data, no objective measure of physical activity, limited generalizability	Funded by INSERM, ANR, French public authorities, and regional health departments

					ow-up		70%		year and was added to the standard curriculum (three 50-min physical education sessions per week)		beyond the specific region	
Haerens (34)	2006	Clustered Randomized Controlled Trial (RCT)	Belgium	15 middle schools in West-Flanders, Belgium	2 years	2,840	girls 36.6%		the program included environmental modifications (3) and interventions on personal and social levels related to food choices and physical	Regular school curriculum (control group)	Limited to schools with technical/ vocational education, higher implementation levels correlated with stronger	Funded by the Flemish Government Policy Research Centre for Sport, Physical Activity, and Health

									activity behavior. The aim of the intervention was to help children to create a physically active lifestyle, together with a healthy diet.		effects, generalizability to boys was weaker	
Weeks (35)	2008	Randomized Controlled Trial (RCT)	Australia	High school PE classes	8 months	99	(46 boys, 53 girls)		10-minute jumping activity in place of regular PE warm-ups, 2 sessions per week	Usual PE warm-up with light jogging and stretching	Small sample size, no long-term follow-up to assess maintenance of bone improvements	Not reported

DL Dewar et al (36)	2013	rct	australia		24 month	357	girls only	12 month & 24 month	The 12-month multicomponent intervention was guided by social cognitive theory and involved strategies to promote physical activity, reduce sedentary behaviors, and improve dietary outcomes. Intervention components included	none	the use of self-report measures to assess changes in screen time and dietary behaviors, and poor accelerometer compliance. In addition, body fat was determined using bioelectrical impedance,	This research project is funded by an Australian Research Council Discovery Project Grant (DP1092646).

									enhanced school sport sessions, lunchtime physical activity sessions, nutrition workshops, interactive educa-tional seminars, pedometers for self-monitoring, student hand-books, parent newsletters, and text messages to reinforce and encourage		which can be influenced by body position, hydration status, and recent physical activity. Although direct measures, such as dual energy x-ray absorptiometry, provide a more accurate assessment of body fat, they	

									targeted health behaviors		may not be feasible in school-based research studies. Finally, because of particip ant attrition, the analyses were underpo wered to detect small changes in BMI. This factor, combin ed with lack of measure	

											ment precision, may have prevented detection of relatively large intervention effects in behavioral outcomes.	
Robbins (37)	2006	Pretest-Posttest Control Group	United States	Two middle schools located in low socioeconomi	12 weeks	77	girls	No long-term follow-up reported (12 weeks intervention)	To encourage PA, each girl in the intervention group received computerized, individually tailored	Control group received age-specific PA recommendations	First, the 12-week intervention period may have been too brief Second,	Funding to conduct the study was received from The Robert Wood Johnson Foundation. We thank the University of Michigan

		Design		c geographic areas in the Midwest					feedback messages based on her responses to the questionnaires, individual counseling from the school's pediatric nurse practitioner (PNP), and telephone calls and mailings from a trained research assistant.	but no tailored feedback or nurse counseling	the exclusive use of self-reported PA is a limitation of this investigation.Third, the small sample size may have precluded the ability to detect significant differences between the groups.	Health Media Research Laboratory for developing the computer program.

Meinhardt (38)	2013	Randomized Controlled Trial (RCT)	Switzerland	Public primary and secondary schools near Zurich, Switzerland	19 weeks training, 3-month washout period	102	boys & girls		School-based strength training program (twice weekly during PE classes) focusing on seven basic exercises: barbell squats, back extensions, crunches, bench press, bent-over rows, and overhead press	Regular physical education classes (control group)	The intervention showed limited impact on girls' PAEE; differences in pubertal development between boys and girls may have influenced results	Supported by the "Stiftung Wachstum Pubertät Adoleszenz" (Foundation for Pubertal Growth and Adolescence)

Bakhoya (39)	2016	Randomized Controlled Trial (RCT)	United States (Midwest urban schools)	Three urban schools with similar demographic characteristics in the Midwest	17-week physical activity intervention followed by a 9-month follow-up period	181	girls	Baseline, Post-intervention, and 9-month follow-up	The Booster Intervention included 13 motivational, individually tailored postcards, enclosed in bright pink envelopes mailed to each girl's home during the postintervention period. GOTM intervention was the no postcard booster	Control group received no intervention	Small sample size, self-reported postcard evaluations, attrition rate	Funded by the National Heart, Lung, and Blood Institute

									intervention			
Greve (40)	2015	Randomized Controlled Trial (RCT)	Denmark	Primary and lower secondary schools in Odense Municipality	3 years (2009/10 – 2011/12)	17000		Baseline and 3 years	HSN programme is to communicate information about the health status of the children involved (based on the health measurements) via the 42 J school health committee and the HSN web-	No health-promoting interventions during the study (control group)	Treatment effects would presumably have been larger if the randomization into treatment and control schools had been restricted to a group	Funded by the Rockwool Foundation

									based platform. The interventi on is also intended to affect the behaviour of teachers and schools. Thus, measuring health indicators for the children and providing informati on on the results are intended to increase the		of schools with a (strong) desire to particip ate in the interven tion.Sec ond, even though the HSN program me, through its focus on the importa nce of informa tion about health, may	

									awareness of teachers and school heads of the state of health of the children in their schools. Schools are encouraged to integrate the measurements into their teaching, and the website presents a list of suggestio		induce some treatment schools to engage in other health promoting initiatives as well, the programme may work in the opposite direction at other schools, crowding out such	

									ns as<br>to how<br>this could<br>be done		initiativ<br>es.<br>Third,<br>the<br>implem<br>entation<br>s of the<br>HSN<br>program<br>me at<br>individu<br>al<br>schools<br>differed<br>greatly<br>Given<br>that the<br>interven<br>tion<br>schools<br>did not<br>use the<br>feedbac<br>k<br>mechani<br>sms<br>fully<br>and the<br>students	

											in the control and treatment schools were already measured to some extent (and were informed about their individual measurements), the true effect might be rather small.	

Zhao (41)	2022	Randomized Controlled Trial (RCT)	China	Secondary schools in China	10 weeks of intervention	123	male	Baseline and Post-intervention (10 weeks)	Comprehensive Strength Training Program (CST): 6-8 strength training exercises targeting upper and lower body, 3 sessions/ week for 10 weeks	Regular PE curriculum focusing on volleyball and football	No follow-up after the intervention, no mid-point testing during the study to measure progressive changes	Funded by the National Key Research and Development Program of China
Torbeyns (42)	2017	Randomized Controlled Trial (RCT)	Belgium	Secondary School (3rd and 4th grade adolescents)	5 months	56			participants assigned to the intervention group were instructed to cycle on a height adjustable	Regular classroom setup without any bike desks	Cycling intensity not standardized, some dropout of participants, no long-term	No funding reported

									cycling desk (LifeSpan C3-DT5 Bike Desk) for 4 class hours (4 × 50 minutes) per week.		follow-up	
Hollis (43)	2016	Cluster Randomized Controlled Trial (RCT)	Australia	Secondary schools in socio-economically disadvantaged communities	24 months	1150	51% femal	Baseline, 12 months, 24 months	The school-based intervention included seven physical activity strategies targeting the following: curriculum (strategies to	Control group received regular school physical education	Small effect size on BMI, missing data for subgroup analysis, generalizability limited to socio-economically disadva	Funded by the NSW Ministry of Health

				in New South Wales (NSW)					maximise physical activity in physical education, student physical activity plans, an enhanced school sport programme); school environment (physical activity during school breaks, modification of school policy); and parents and the		ntaged schools	

									community (parent engagement, links with community physical activity providers)			
Børrestad(44)	2012	Randomized Controlled Trial (RCT)	Norway	A public school in Kristiansand, Norway	12 weeks	53	53% boys	Baseline and post-intervention (12 weeks)	The intervention group was encouraged to cycle to and from school every school day for 12 weeks; control group received no intervention	Control group received no intervention	No significant effect due to cycling culture in Norway, re-analysis indicated results for those who cycled regardless of	Funded by the University of Agder

											randomization	
Robbins (45)	2019	Group Randomized Controlled Trial (RCT)	United States	24 schools (12 intervention, 12 control) in the Midwestern United States	17 weeks	1,519	girls	Baseline, post-intervention, 9-month follow-up	The intervention included three components: (a) two face-to-face motivational interviewing sessions (one at the beginning and the other at the end of intervention) with a health	Control group received usual school activities	Limited generalizability due to specific population (urban, low-income girls), self-reported data, social desirability bias	National Heart, Lung, and Blood Institute (NHLBI)

									professional to address each girl's unique perceived PA benefits and barriers to motivate PA change (HPM, benefits and barriers; SDT, motivation); (b) an after-school PA club offered 3 days a week by club coaches to			

									provide fun PA opportunities and coach and peer support to increase girls' PA skills (HPM, enjoyment, self-efficacy, social support); and (c) an interactive Internet-based session delivered at the intervention midpointvia an iPad to provide			

									each girl individually tailored motivational and feedback messages based on her survey responses to encourage PA (SDT, motivation).			
Arlinghaus (46)	2021	Randomized Controlled Trial (RCT)	United States (Houston, Texas)	Middle schools in a low-income district in Houston, Texas	6 months	491	53% girls	Baseline and 6-month follow-up	The intervention consisted of the physical activity component of an obesity intervention with established efficacy	Traditional PE class as usual (control group)	Self-reported data for some measures, lack of long-term follow-up, variability in MVPA data	Funded by the US Department of Agriculture

									at reducing standardized BMI among this population.46-48 Only the physical activity component of the obesity intervention was included. No nutrition education was provided as part of the current intervention. Intervention			

									activities were rooted in Social Cognitive Theory.49 Trainedresearch staff partnered with PE teachers to facilitate lessons and undergraduate college students weretrained to complete activities with participants.			

Peralta (47)	2009	Randomized Controlled Trial (RCT)	Australia (Sydney, NSW)	Single boys' secondary school in Sydney, Australia	6 months (16 weeks)	33	boys	Baseline and 6-month follow-up	The FILA Program: 60-minute curriculum session and two 20-minute lunchtime sessions per week, focusing on PA, self-regulation, screen time reduction, and diet	Regular PE lessons (Friday afternoons)	Small sample size, lack of long-term follow-up, no true control group, limited generalizability as it was conducted in a single school	Partly funded by the intervention school

Kim (48)	2011	Randomized Controlled Trial (RCT)	South Korea	Kyung Hee University, Yongin City, South Korea	12 weeks	31	Female	Baseline and Post-intervention (12 weeks)	The taekwondo group participated in their required school physical education classes (50 min a day, 2 days a week) and two 50-min periods of afterschool taekwondo training per week for 12 weeks. The training programme was designed	Control group followed regular PE curriculum	Small sample size, short duration of training, no significant cardiorespiratory improvements, and lack of gender-based analysis	Not mentioned

									for beginners and utilized basic taekwondo movements. Each training session consisted of a 10-min warm-up (joint rotations, muscle stretching, and body twisting), 10 min of Poomsae for beginners (basic blocking, punching,			

									and hand technique drills), 10 min of basic stepping and kicking drills, 10 min of basic stepping and target kicking drills, and a further 10 min of joint rotations, muscle stretching, and body twisting			
Grydel and (49)	2013	Cluster-Rando	Norway	37 schools in sout	20 months	2165	IGGIRLS 54	Baseline and 20 months post-	The HEIA study is based on a socio-ecological	Regular school curriculum	Seasonal variations in PA	Funded by Norwegian Research Council and Throne Holst

		mized Controlled Trial (RCT)		h-eastern Norway			% CGIRLS 60%	intervention	framework that aims to combine personal, social and physical enviroAnmental factors hypothesized to influence overweight and obesity in children, mediated by dietary and physical activity behaviors		assessments, attrition of accelerometer data, and no follow-up beyond 20 months	Nutrition Research Foundation
Lubans (50)	2010	Cluster-randomiz	Australia	6 secondary schools	6 months	124	Boys: 53, Girls: 71	6 months	The Program X intervention was delivered	Control group received the same	No significant mediators identifie	University of Newcastle Internal Grant

		ed con trol led trial		in New castl e, New Sout h Wal es					as an extra-curricular school sport program and involved five major componen ts: (i) enhanced school sport program focusing on lifetime physical activities, (ii) informati on sessions and summary interactiv e lecture, (iii)	school sport comp onent witho ut additi onal behavi oral suppo rt	d, small sample size, lack of clusteri ng control	

									pedometers for physical activity monitoring, (iv) physical activity and nutrition handbooks with monthly information newsletters for parents, and (v) social support for healthy behaviors through e-mails			

Aittasalo (51)	2019	Cluster-randomized controlled trial	Finland	14 public secondary schools in Tampere, Finland	4 weeks for follow-up 1, 7 months for follow-up 2	1476	48.2% girls in intervention group, 47.4% in control group	4 weeks for Follow-up 1, 7 months for Follow-up 2	The evaluation was based on RE-AIM: Effectiveness was assessed from baseline to 4 weeks (Follow-up 1) and Maintenance from 4 weeks to 7 months (Follow-up 2) with change in students' PA and SB and related psychosoc	Control group received standard health education (HE) lessons	Short follow-up duration, modest effects noted, self-reported data limitations	Ministry of Education and Culture

									ial and parental factors In INT a new content on PA guided by the Health Action Process Approach -model [25] was integrated into three routinely scheduled HE lessons (Lessons #1–3, Table 1). All teachers in INT received one-hour			

									training from the researchers and a Teacher's Manual to deliver the lessons. The manual included detailed description of the contents and material for each lesson; class-specific student lists for each lesson, where the teachers			

									were asked to keep record of students' attendance; and space for additional notes or comments.			
Carlin (52)	2018	Cluster randomized controlled trial (RCT)	Northern Ireland (UK)	Six post-primary schools in Northern Ireland	12 weeks	199	girls	6 months post-intervention	**Intervention Group: Peer-led brisk walking sessions (10-15 minutes) conducted throughout the school day**	Control group continued usual physical activity	Small sample size, short intervention period, lack of sustained effects at follow-up, accelerometer	Supported by Vice Chancellor's Research Scholarship from the University of Ulster

											limitations	
Singh (53)	2009	Randomized Controlled Trial (RCT)	Netherlands	10 intervention schools and 8 control schools (prevocational secondary schools)	20-month follow-up post-intervention	1108	boys and girls	Baseline, 8 months, 12 months, 20 months	11 lessons integrated into biology and physical education curriculum; environmental changes in schools (e.g., offering more PE classes)	Control group received regular curriculum	Self-reported dietary behavior data, limited effect on BMI, possible reporting bias	Funded by the Netherlands Heart Foundation and Dutch Ministry of Health
Ardoy (54)	2011	Group Rando	Spain	Public secondar	16 weeks	67	35.8% girls	Baseline (T0), post-	EG1 (n = 26) doubled the	the CG (n = 18) receiv	Small sample size, single	Funded by European Commission projects

		mized Controlled Trial (RCT)		y schools in Murcia, Spain				intervention (T1, 16 weeks)	academic load stipulated for this subject (four 55-minute sessions a week. EG2 (n = 24) also received 4 sessions/ week in which there was special emphasis on increasing the intensity of the sessions (4 x 55 min/session plus intensity).	ed the 2 sessions of PE a week (55 min/session) established by the regulations currently in force in Spain	school setting, lack of long-term follow-up	(HELENA and ALPHA) and Murcia Department of Education

Belton (55)	2019	Cluster randomized controlled trial	Ireland	20 mixed-gender post-primary schools	24 months	490		12 month and 24 months	Y-PATH whole-school intervention: PE component, whole-school teacher component, parent component.	Control group continued with usual PE curriculum.	Study limitations: Small sample size, especially at 24 months.	Funding: Dublin Local Sports Partnerships, Dublin City University.
Okely (56)	2017	Group Randomized Controlled Trial (RCT)	Australia	24 secondary schools in urban, regional, and rural areas of	18 months	1518	Girls	Baseline (T0), Post-intervention (T1, 18 months)	Using a Health Promoting Schools and Action Learning Frameworks, each school formed a committee and developed an action	Control group continued usual school activities	Low accelerometer compliance at follow-up, implementation challenges in schools	Funded by the New South Wales Department of Education and Communities

				New South Wales, Australia					plan for promoting physical activity among Grade 8 girls. The action plan incorporated strategies in three main areas – i) the formal curriculum, ii) school environment, and iii) home/school/community links – based on the results of			

									formative data from target girls and staff and on individual needs of the school. A member of the research team supported each school throughout the intervention			
Dewar (57)	2014	Group Randomized Contro	Australia	12 secondary schools in low-	12 months	357	Adolescent girls	Baseline (T0), Post-intervention (T1, 12	The intervention included enhanced school sport, lunchtime	Waitlist control group (no interv	Poor accelerometer compliance, small sample size,	Australian Research Council Discovery Project Grant

		lled Trial (RCT)		income communities in Hunter and Central Coast regions of New South Wales, Australia				months)	physical activity sessions, interactive seminars, student handbooks, nutrition workshops, pedometers, parent newsletters and text messages to encourage physical activity and healthy eating, and a decrease in sedentary behavior	ention)	lack of long-term follow-up	

ka wing Ho (58)	2017	Randomized Controlled Trial (RCT)	Hong Kong, China	12 secondary schools across 4 districts in Hong Kong	18 weeks of after-school sports sessions	664	[58.1% girls	Baseline (T0), Post-intervention (T1, 1 month after completion)	Intervention Group: PYD-based sports mentorship sessions (90 minutes weekly for 18 weeks); Control Group: Access to a health education website	Control group received online health education via quiz game	Self-reported physical activity, no long-term follow-up on mental health	Funded by Freddy Zimmern Sports Foundation
Gallotta (59)	2009	Randomized Controlled Trial (RCT)	Italy	Middle schools in Rome, Italy	5 months	152	boys and girls	Baseline (T0) and Post-intervention (T1, after 5 months)	The experimental intervention was structured in four different didactic modules focused	Traditional PE focusing on basic motor skills, endurance, and	Small sample size, limited to one city, and no long-term follow-up	Not reported

									on co-ordination abilities: pre-tumbling, rhythmic gymnastics, ball mini-games, and dexterity circuits.	fitness exercises		
Ha (60)	2019	Cluster randomized controlled trial	Hong Kong	26 secondary schools	8 PE lessons (follow-up data collected after the inte	667	69% female, 31% male		Students in the experimental group received the SELF-FIT intervention, a school-based intervention designed to infuse fitness	A wait-list control group that continued using regular PE teaching methods.	Some teachers did not use music due to school policy, and some schools could not use digital devices.	Supported by the General Research Fund, University Grants Committee, Hong Kong.

					rvention)				and game-like elements into PE using self-determination theory principles			
Annan (61)	2021	Longitudinal School-Based Intervention Study	Ghana	Public primary schools in Kumasi Metropolis, Ghana	6 months	433		Baseline, 3 months (mid), 6 months (post-intervention)	Nutrition Education Group: Nutritional lessons, balanced diet practices, nutrient deficiency awareness; Physical Activity Group: Physical fitness education; Combined Intervention Group:	Control group received no intervention	Lack of control over participants' out-of-school activities, potential cross-communication between control and intervention groups	Funded by the University of Tokyo, Japan

									Both interventions			
Juric (62)	2023	Cluster randomized controlled trial (RCT)	Croatia	Physical education classes in Zagreb schools	12 weeks	207		Post-intervention (12 weeks)	**The intervention provided two 10-minutes HIIT sessions per week at the beginning of regular PE classes. The intervention lasted 12 weeks, offering 24 sessions across the whole semester**	Control group had regular PE classes as per the embedded curriculum	Short duration, no heart rate monitoring for precise intensity measurement, lack of eating habits data, small sample size	Croatian Science Foundation

									**to students involved in the HIIT program. One PE teacher delivered sessions to all participants.**			
Lubans (63)	2012	Cluster randomized controlled trial	Australia	12 secondary schools in low-income communities in the Hunt	12 months	357 participants	All female	12 months	A multicomponent school-based intervention program tailored for adolescent girls. The intervention was based on	Regular school curriculum	Poor compliance with accelerometer monitoring, reliance on self-reported screen time	Australian Research Council

				er and Central Coast regions, New South Wales					social cognitive theory and included teacher professional development, enhanced school sport sessions, interactive seminars, nutrition workshops, lunch-time physical activity sessions, handbooks and pedometers for self-monitorin			

									g, parent newsletters, and text messaging for social support.			
Smith (64)	2014	Cluster randomized controlled trial	Australia	14 secondary schools in low-income communities in New South Wales	20 weeks	361 boys	Male	8 months (immediate post-intervention follow-up)	The 20-week intervention was guided by self-determination theory and social cognitive theory and involved: teacher professional development, provision of fitness equipmen	Usual school sports and physical education lessons (control group, n = 180)	Lack of significant effects on primary outcomes (BMI, waist circumference), moderate compliance with accelerometer protocols, absence	Australian Research Council Discovery Project grant (DP120100611).

									t to schools, face-to-face physical activity sessions, lunchtime student mentoring sessions, researcher-led seminars, a smartphone application and Web site, and parental strategies for reducing screen-time		of usage data for smartphone app.	

Polo-Recuero (65)	2023	Randomized Controlled Trial (RCT)	Spain	Public high school in Madrid	10 weeks	55	46.4% girls in the intervention group	Baseline, 10 weeks (post-intervention)	The aim of this study was to assess the effects of a classroom-based physical activity program, using bike desks, on academic and physical performance in adolescents. The Program to Enhance and Develop Active Lessons (PEDAL) was	Control group continued regular language arts lessons without bike desks	Small sample size, short duration, no long-term follow-up	No external funding

									designed for this purpose, expecting an increase in students' physical activity without any decrease in academic performance. This intervention based on pedal or bike desks—stationary bikes that integrate with a desk workspace			

Weeks (66)	2012	Randomized Controlled Trial (RCT)	Australia	Secondary school (Gold Coast, Queensland, Australia)	8 months	99	boys and girls	Baseline (T0), 8 months (T1)	Intervention Group: Twice-weekly 10-minute jumping exercises (jumps, hops, lunges) replacing regular warm-ups	Control Group: Usual warm-ups (walking, light jogging, stretching)	Limited to one school, relatively small sample size, no long-term follow-up	No external funding sources mentioned
Reed (67)	2013	Randomized Controlled Trial (RCT)	United States (Southeast)	Title I public charter elementary and middle schools	1 school year (September 2009 to May 2010)	470	boys and girls	Baseline (T0) and Post-intervention (T1, after 1 school year)	In the fall of 2009, the experimental school implemented a comprehensive, multifaceted approach to	Standard PE curriculum (less frequent PE sessions for control group)	Differences in sample size between experimental and control schools, limited generalizability due to	Not mentioned

									education based on the premise that a ‘sound body nurtures a sound mind.’ Two certified physical education teachers were hired to provide 45 minutes of daily physical education, 5 days a week to all children in all grades.		Title I school populati on	

									The physical education requirements in this southeastern school district for elementary schools (ie, control) is 45 minutes 1 day a week for grades 1st–5th and 30 minutes 1 day a week for kindergarteners. Middle schools (ie, control) provide			

									50 minutes of daily physical education for 1 semester in grades 6th–8th. Physical education at the experimental school and both control schools were taught by certified physical education instructors. Elementary school physical education			

									content at both the experimental school and control school used a developmental curriculum with an emphasis on fundamental skills. Middle school physical education at both the experimental school and control school			

									used a multiactivity sport theme curriculum. Control middle school students in grades 6th–8th received 50 minutes of daily physical education daily for the fall semester only. Elementary control school students in grades 2nd–5th received			

									only 45 minutes of PE 1 day per week for the entire school year.			
Guijarro-Romero (68)	2022	Cluster-randomized controlled trial	Spain	One secondary school in Ciudad Real	9 weeks (initial) + 6 weeks (reinforced)	107	48% male, 52% female	Post-intervention and post-maintenance	First, the NRG and RG received a physical fitness-based development program twice a week for nine weeks. Due to	Control group with usual PE lessons	Small sample size, weather impacted outdoor sessions	Not mentioned

									meteorological problems, in the end only 16 sessions were completed by both RG and NRG. Then, after a four-week detraining period, the RG participants completed a reinforced program twice a week for six weeks.			

Murphy (69)	2024	Cluster Randomized Controlled Trial (RCT)	Ireland and Northern Ireland	Secondary schools in the border region of Ireland and Northern Ireland	19.9 ± 0.97 weeks	589	female	12.2 months	Peer-led walking intervention with walks before school, at break, and lunch recess	Usual practice (no intervention)	High pupil absenteeism, lower-than-expected participation in walks, and potential impact of COVID-19 pandemic restrictions	Not specified
Sutherland et all (70)	2016	CRCT	Australia	Secondary Schools located in disa	24 months	1150	49% male	12 months (mid-intervention); and 24 months	A multicomponent school-based intervention called Physical	Schools allocated to the control group	1)A limitation of the study is the loss of participants at	The Physical Activity 4 Everyone intervention trial was funded by the New South Wales

				dvantaged communites				postrandomization follow-up.	Activity 4 Everyone (PA4E1). Here are the key components: Curriculum Strategies School Environment. Parent and Community Engagement Support Strategies	participated in the measurement components of the trial only and delivered physical activity teaching and promotion practices according to the PE curric	follow-up. 2)The use of accelerometers as an objective measure of physical activity had compliance issues.3)The use of accelerometers as an objective measure of physical activity had	Ministry of Health through the New South Wales Health Promotion Demonstration Research Grants Scheme and conducted by Hunter New England Population Health (a unit of the Hunter New England Local Health District), in collaboration with the University of Newcastle and University of Wollongong. Infrastructure support was provided by

										ulum and school -based initiati ves	complia nce issues.	Hunter Medical Research Institute.
Rostam i-Moez (71)	201 7	Ran do miz ed Co ntro lled Tri al (R CT)	Ira n	Mid dle scho ols in Ham adan , Iran	8 mon ths (2 mon ths of edu cati on, 6 mon ths foll ow- up)	314	girl s		A theory-based education al interventi on designed using PRECED E and Trans-Theoretic al Models. The program included health education on physical activity	Stand ard curric ulum with no additi onal physic al activit y educat ion	Limited to 7th-grade girls, no objectiv e measure s of physical activity (e.g., accelero meters), reliance on self-reported question naires	Funded by the Vice-Chancellor of Research and Technology, Hamadan University of Medical Sciences

									and self-monitoring tools like logbooks and sports-related activities.			
Isensee et all (72)	2018	Cluster-Randomized Controlled Trial	Germany	School-based	1 year	1489	Both	1 year	The intervention was a 12-week school-based program where students used pedometers to monitor their daily steps, participated in class competitions to encourage	No intervention	PA was only assessed through self-administered questionnaires which could lead to biased information. However, as pedometers were an	The ‘‘lauft.’’ trial was funded by German Cancer Aid in the Priority ProgramPrimary Prevention of Cancer (Nutrition and Physical Activity, reference numbe ̈ r: 110012). The ‘‘lauft.’’ trial ̈ is a cooperation project

									physical activity, documented their progress online, and engaged in four educational lessons on integrating physical activity into daily life . - Pedometer use (Omron Walking Style One 2.1)		essential intervention component, they could not be applied to the control group as well. In addition, using self-reports is easy to administer and cost-effective in large sample sizes. Informa	between the Institute for Therapy and Health Research, University of Kiel, University of Hamburg,

											tion might be more likely biased in favor of the interven tion group because students received targeted message s about the benefits of PA. In addition , a clear distincti on between differen t intensiti es of	

											PA could not be made, which would have added value on the interpretation of the results. Another drawback is that students in the control group participated in more out-of-school sports activities and	

											weekly MVPA than students in the intervention group.	
Dudley (73)	2010	Pilot Randomised Controlled Trial	Australia	Secondary schools in south-west Sydney	3 months	38	girls	3 month	The intervention was implemented during school sport over the course of an 11-week school term. There were six fortnightly sessions over the term with each lasting 90 min.	Usual school sport program	Single Site Implementation: The study was conducted in only one school, which may limit the generalizability of the findings to other educatio	Not reported

									School sport was programmed to take place immediately after the scheduled Thursday lunch break. Participants discussed what physical activities they enjoyed, or were interested participating in during focus groups prior to randomisa		nal settings.<br>Limited Statistical Power: The small sample size restricts the study's ability to detect meaningful changes or effects.<br><br>Insensitivity of Measures: Existing measures may not be	

									tion. The researcher and teaching staff from the school designed a sport program to reflect those areas of enjoyment and interest identified in the focus groups.		sensitive enough to capture changes in enjoyment specifically related to the school sport sessions, as opposed to overall physical activity.<br><br>Duration of Accelerometer Use: Acceler	

											ometers should be used over a longer period to detect significant changes in total physical activity accurately.<br><br>Activity Nature and Context: The type of physical activity needs to be recorded and interpret	

											ed relative to the control group's activities, taking into account their MET (Metabolic Equivalent of Task) values for appropriate comparison.<br>Self-Selected School: The school’s self-selectio	

											n may affect the applicability of the results to other schools.<br><br>Researcher Influence: The researcher facilitated focus groups, which may have led to socially desirable responses from particip	

											ants, potentially biasing the results.	
Ridgers ET AL (74)	2021	CRCT	Australia	Schools in socio-economically disadvantaged areas	12wl	275	50.2% female	12 weeks and then 6months	The Raising Awareness of Physical Activity (RAW-PA) Study was a 12-week, multicomponent interventi	none	Limited Feedback from Fitbit Flex: The Fitbit Flex provided minimal real-time feedbac	This study was supported by a VicHealth Innovation Research Grant. NDR is supported by a National Heart Foundation of Australia (NHFA) Future Leader

									on that combined a Fitbit Flex (and accompanying app), and online digital behaviour change resources and weekly challenges delivered via Facebook		k, requiring participants to engage with the app or website for detailed activity data. This may have hindered their ability to self-monitor activity levels effectively and track progress toward their	Fellowship [Award ID 101895]. AT was supported by a NHFA Future Leader Fellowship [Award ID 100046] at the time of the study. KB was supported by a National Health and Medical Research Council (NHMRC) Principal Research Fellowship [APP1042442] at the time of the study. JS is supported by a NHMRC

											goals.<br><br>Low Compliance with Accelerometer Use: Although the retention rate was relatively high, compliance with the accelerometer measure was low, with only about half of	Leadership Level 2 Investigator Grant [APP1176885]. The content of this manuscript is the responsibility of the authors and does not necessarily reflect the views of the funding bodies

											the sample providing complete valid data. This aligns with findings from previous trials.<br><br>Self-Reported Recruitment: Recruitment was based on self-reporting against	

											inclusion criteria, which means the number of eligible adolescents is unknown. Participants who agreed to take part may not accurately represent the broader population, potentially	

											affecting the study's generalizability.	
Jago, R., Sebire, S.J., Cooper, A.R. et a (75)	2012	Feasibility trial	United Kingdom	schools	20 weeks	203	female	Time 1 (8 weeks) Time 2 (20 weeks)	Intervention participants received a 9-week dance program with 2, 90-minute dance classes per week. Participants at 2 control schools received	Control (incentives only)<br><br>Control (incentives + delayed dance workshop)	Limited Participation: Only 30 girls per school participated in the intervention, meaning not all girls in the year group benefitted from	This project was funded by the National Prevention Research Initiative (http://npri.org.uk), consisting of the following funding partners: Alzheimer's research Trust; Alzheimer's Society;

									incentives for data collection. Participants at 2 additional control schools received incentives and a delayed dance workshop		the program.<br><br>Potential Bias from Taster Sessions: While the taster sessions encouraged some less active participants to join, they may have discouraged others	Biotechnology and Biological Sciences Research Council, British Heart Foundation; Cancer Research UK; Chief Scientist Office; Scottish Government Health Directorate; Department of Health; Diabetes UK; Economic and Social Research Council; Engineering and Physical Sciences Research Council;

											from enrolling. This could result in a sample skewed toward girls who enjoyed the taster session.<br><br>Representation of Activity Levels: Although the sample included girls who	Health & Social Care Research & Development Office for Northern Ireland; Medical Research Council; The Stroke Association; Welsh Assembly Government and World Cancer Research Fund. This report is also research arising from a Career Development Fellowship (to Dr Jago) supported by the National Institute for

											were identified as "low active," their overall physical activity levels were lower than average for their age group. This may limit the generalizability of the findings to more active populations.	Health Research. The views expressed in this publication are those of the authors and not necessarily those of the NHS, the National Institute for Health Research or the Department of Health. This work was also supported by DECIPHer, a UKCRC Public Health Research: Centre of Excellence. Funding from

												the specific funders under the auspices of the UK Clinical Research Collaboration, is gratefully acknowledged.
Kolle, E., Solberg, R.B., Säfvenbom, R. et al. (76)	2020	Cluster Randomized Controlled Trial (RCT)	Norway	Lower secondary schools	September 2016 to July 2018.	2733	Girls and Boys	14 months	The physically active learning (PAL) intervention included 30 min physically active learning, 30 min PA and a 60 min	Control Group: Usual practice	Loss to Follow-up: Greater for accelerometer data than for physical fitness data.<br><br>Compliance	The study was funded by the Norwegian Directorate for Education and Training. The funding body had no involvement in design of the study, data collection, analysis,

									physical education (PE) lesson per week. The Don’t worry-Be happy (DWBH) intervention included a 60 min PA lesson and a 60 min PE lesson per week, both tailored to promote friendships and wellbeing. Both intervention arms were designed		Issues: Adherence to wearing accelerometers.<br><br>Activity Detection Limitations: Accelerometers may not capture all activities.<br>Self-led vs. Teacher-led Activities: Potential differences in	interpretation of data or in the drafting of the manuscript.

									to engage the adolescents in 120 min of PA per week in addition to recess and mandatory PE lessons.		intensity and effectiveness.	
Lonsdale (77)	2019	Cluster Randomized Controlled Trial (RCT)	Australia	Secondary schools in low socio-economic areas of Western	7–8 months (post-intervention), with a maintenance pha	1421		14–15 months (maintenance phase)	The Activity and Motivation in Physical Education (AMPED) intervention for secondary school PE teachers included workshops, online	Standard PE lessons (usual care)	Limited generalizability to higher socioeconomic areas; reliance on accelerometers for measuring MVPA	Australian Research Council Discovery Grant

				Sydney	se of 14–15 months				learning, implementation tasks and mentoring sessions			
Morgan (78)	2012	Randomized Controlled Trial (RCT)	Australia	Secondary schools in disadvantaged areas in Hunter Region, New South Wales, Australia	6 months	100	boys	3months and then -6months	PALs was a multi-component school-based intervention that included enhanced school sport sessions with a focus on resistance training, physical activity and nutrition handbooks with home-	Wait-list control group	Small sample size, limited statistical power for psychosocial outcomes, and restricted to boys in disadvantaged schools	Supported by the Hunter Medical Research Institute and the Rotary Club of Newcastle Enterprise

									based challenges, interactive seminars addressing key lifestyle physical activity and nutrition behaviours, leadership principles and self-directed lunch-time exercise sessions. The boys were encouraged to become physical			

									activity leaders in their schools and at home and accreditation was provided to students who complied with the programme.			
Singh (79)	2007	Randomized Controlled Trial (RCT)	Netherlands	18 prevocational secondary schools in the Neth	8 months	1053	both	8 month	The Dutch Obesity Intervention in Teenagers (DOiT) program, a multicomponent health promotion	Regular school curriculum (control)	The study population consisted solely of students from the lower educational	Funded by the Netherlands Heart Foundation, Dutch Ministry of Health, Welfare, and Sports, and the Royal Association of Teachers

				erlands					program including 11 lessons in biology and physical education, additional PE classes, and changes to school canteens		levels of the Dutch school system, limiting generalizability; limited effect sizes, particularly for BMI and fitness outcomes	of Physical Education
Lubans (80)	2009	Randomized Controlled Trial (RCT)	Australia	Six secondary schools	6 months	124	58 participants (30 boys and 28 girl		A 10-week school sport program supplemented with pedometers for self-monitoring,	Standard school sport program	Small sample size, use of pedometers only for self-reported steps,	upported by Friends of the University of Newcastle

							s) were in the intervention group, and 66 participants (23 boys and 43 girls)		parental materials, and weekly e-mail support. Intervention also included physical activity and dietary monitoring using diaries		limited parental involvement, no long-term follow-up	
jago et al (81)	2021	cluster-randomiz	south west En	secondary schools	6 months	1558	adolescent	5 to 6 months	Schools were randomly assigned to the	Usual practice<br><br>Comp		

		ed controlled trial (RCT)	gland,				girls.		PLAN-A intervention or a non-intervention control group after baseline data collection. Girls nominated students to be peer leaders. The top 18 % of girls nominated by their peers in intervention schools received three days of training designed	onents:		

									to prepare them to support physical activity			
Shamah Levy (82)	2012	Cluster-randomized field trial	Mexico	Public elementary schools in the State of Mexico	6 months	1020	both		"Nutrition on the Go" strategy aimed at reducing energy content in school breakfasts, increasing consumption of fruits and vegetables, and promoting physical activity and water consumption	Usual school routine with no changes in diet or physical activity promotion	Limited to school hours (4.5 hours /day), intervention duration was only 6 months, lack of parent participation	State System for the Comprehensive Development of the Family (DIFEM), State of Mexico

Costigan (83)	2018	Randomized Controlled Trial (RCT)	Australia	Secondary school in Newcastle, Australia	>8 weeks	65	45 males, 20 females		Participants completed 24 HIIT sessions over 8 weeks (three sessions per week). The sessions included cardiorespiratory exercises (e.g., shuttle runs, jumping jacks) or a combination of resistance and aerobic training	Control group engaged in usual PE lessons and lunchtime activities	Small sample size, short intervention period, limited generalizability due to single school setting	Supported by a Hunter Medical Research Institute Project Grant

Ana Carolina Barco Leme et al (84)	2016	RCT	Brazil	School	6 months	253	girls only		The H3G-Brazil program was a 6-month multicomponent school-based intervention guided by the social cognitive theory (SCT)). The intervention was based on ten nutrition and physical activity (PA) messages to support	none	self-report measures of PA, screen time, and dietary intake. In addition, an inclusion of 12-month assessments would provide evidence for the distal impact of the 6-month intervention. Indeed, while it	

									healthy eating and regular PA. Additional program components were designed to reinforce healthy dietary and PA behaviors and included enhanced physical education (PE) sessions, school-break PA sessions, nutrition and PA handbook		is possible that the H3G-Brazil intervention on body composition and health behaviors may strengthen over time, the opposite may also be true. Although groups were randomized after baseline	

									s, interactive seminars, nutrition workshops, weekly nutrition and PA key messages, parental newsletters, weekly health messages using WhatsApp®, and diet and PA diaries for self-monitoring.		assessments, there were statistically significant differences between groups at baseline that may have influenced the study findings. Finally, although the schools were randomized into	

											intervention and control groups, the study participants were not randomized. Teachers and coordinators invited the girls from the courses that have a great number of female adolescents to voluntar	

											ily particip ate on the study.	
corder et al (85)	202 0	CR CT	UK		12 wks	286 2	**C G ma le 53. 4 % , fe ma le 46. 6 % IG ma le 51. 1**		. GoActive aimed to increase physical activity through increased peer support, self-efficacy, selfesteem, and friendship quality, and was implemented in	none	The main method ological limitation of this study was the relatively affluent and ethnically homogeneous sample.it is	This study is funded by the National Institute for Health Research (NIHR) Public Health Research Programme

							**% female 489 %**		tutor groups using a student-led tiered-leadership system. Mentorship and peer-leadership addressed time pressures, which were stated by teachers in our development work as being a barrier to participation in activity promotion		likely that an insufficient dose of the intervention was delivered to achieve the desired effect, and it therefore remains unclear whether the GoActive intervention, if delivered as intended, is	

									programmes, and between-class competition was incorporated as a strategy to encourage teacher enthusiasm		effective in changing adolescents' overall MVPA.	
Trajković et al (86)	2020	rct	serbia		8month	152	IG n= 54 (14 girls), CG: n= 51 (16 girls)		Both groups, SG and CG, attended regular physical education (PE) classes twice a week for eight months. In	NONE	Lack of Baseline Physical Activity Data: The overall physical activity levels of students were not assessed	This research received no specific grant from any funding agency in the public, commercial, or non-profit sector.

									addition to PE classes, SG undertook two recreational soccer sessions after school. In total, the SG completed 64 soccer sessions after school: ~45-min SSG sessions per week, separated by at least 1 day. Finally, SG and CG participan		before or during the eight-month intervention, which limits the understanding of its impact.<br><br>No Monitoring of Dietary Intake: The study did not control for dietary habits,	

									ts’ levels of physical fitness and aggression were retested after 32 weeks		which could have influenced the outcomes and acted as a mediator for training gains.<br><br>Contextualization of Aggression: There was no attempt to differentiate between types of	

											aggression (e.g., passive vs. reactive), which could provide deeper insights into the findings.	
Ulrike Maria Mueller et al (87)	2012	rct	germany			491		4year follow up	In brief, we performed a class-wise cluster randomised intervention study in 5th and 6th grade classes that investigated the	none	High Drop-Out Rate: Approximately one-third of participants dropped out, which is consistent with findings	The author(s) disclosed receipt of the following financial support for the research, authorship, and/or publication of this article: an unrestricted grant from Novartis and Roland Ernst Stiftung.

									influence of daily 60 minutes of PE at school (altogether 5 hours per week) in the intervention group in comparison to the regular (in Germany) two PE units (45 minutes each) weekly in the non-intervention control group on cardiovascular parameter		in other pediatric studies. This highlights the challenges of conducting research with children and adolescents.<br><br>No Significant Between-Group Difference in Drop-Out: Althoug	

									s. In addition, lessons on healthy lifestyle were included in the regular schedule once monthly for both groups. A third study group was a pure observational group without intervention and consisted of sport students (high level) at a college of		h the drop-out rate was high, there were no significant differences between groups, making it difficult to assess the impact on outcomes.<br><br>Reasons for Drop-	

									physical education with high level PA in competitive sports that frequently participated in competitive sporting events, thus representing a maximum of physical fitness attainable under reasonable conditions in school-age		Out: Most drop-outs were attributed to changes in classes or schools, or severe protocol deviations from previous school years, which may affect the consistency of the data.	

									children. Most of them were swimmers.		Control Group Limitations: The control group, which was not assigned to receive the intervention, cannot be considered a true "no-treatment control." Awareness of the	

											intervention may have influenced their behavior, potentially increasing their leisure time physical activity or decreasing it as a response to the intervention's presence.	

Corepal et all (88)	2019	rct	ireland	school	6 months	224	Both	post-intervention (at 22 weeks post-baseline and 52 weeks post-baseline)	The StepSmart Challenge used gamification strategies, combined with the core tenets of self-determination theory, to encourage and support physical activity behaviour change by moving participants along the motivation	(no intervention, no Fitbit pedometers, no incentives)	"There were several challenges faced in the study, most notably a high level of missing data from accelerometers at follow-up time points." "Another limitation is that we chose not to underta	This study was funded by the National Institute for Health Research (NIHR) Public Health Research (PHR) programme (grant reference number 12/211/82). RUTH HUNTER is funded by an NIHR Career Development Fellowship (CDF-2014-07-020). The views expressed are those of the author(s) and not

									continuum (from more external forms of motivation, e.g. external regulation) towards intrinsic motivation		ke any formal hypothesis testing, as this was a feasibility trial, the focus being on testing recruitment and retention strategies, and assessing acceptability and outcome measures rather	necessarily those of the NHS, the NIHR, or the Department of Health and Social Care

											than effectiveness." "The generalisability of the findings may also be limited as participating schools had previously been involved in physical activity research conducted by QUB, and may have been	

											more motivated to take part than other schools." "Finally, while the provision of opt-out consent increased participation, it also limited our direct contact with participants'	

											parents which may have contributed to lower accelerometer return rates."	
Lubans (89)	2021	Cluster randomised controlled trial	Australia	20 secondary schools in New South Wales	12 months	670	44.6% female	6 months and 12 months	HIIT activity breaks during curriculum time	Usual practice (no intervention)	35.3% loss to follow-up at 12 months, CRF not sustained at 12 months	Australian New Zealand Clinical Trials Registry (ACTRN12618000293268)
Suchert (90)	2015	Cluster randomized	Germany	29 schools in Ger	12 weeks	1162		Baseline (T0), post-intervention	**Intervention Group: Use of pedometers, class**	Control group received no interv	Self-reported data may introduce bias,	Funded by the German Cancer Aid

		controlled trial (RCT)		many				(T1, 12 weeks)	**competitions to increase physical activity (steps/week and creative ideas to increase activity), and educational lessons**	ention, just usual education	no significant difference in sedentary behavior, possible effects of baseline differences between groups	

S3 File. GRADE

1. **Author(s):**
2. **Question:** Physical Activity compared to Control for promotion of Physical activity in adolescents
3. **Setting:**
4. **Bibliography:** Strategies & Intervention for promotion of Physical activity in adolescents. Cochrane Database of Systematic Reviews [Year], Issue [Issue].

Certainty assessment							№ of patients		Effect		Certainty	Importance
№ of studies	Study design	Risk of bias	Inconsistency	Indirectness	Imprecision	Other considerations	Physical Activity	Control	Relative (95% CI)	Absolute (95% CI)		
**Physical Activity (Intensity) - Light Physical Activity**												
6	randomised trials	not serious	not serious	not serious	not serious	none	930	1070	-	SMD **0.04 lower** (0.13 lower to 0.05	⊕⊕⊕⊕ High	CRITICAL

										higher)		

Physical Activity (Intensity) - Moderate Physical Activity

7	randomised trials	not serious	not serious	not serious	not serious	none	879	687	-	SMD **0.01 higher** (0.16 lower to 0.18 higher)	⊕⊕⊕⊕ High	CRITICAL

Physical Activity (Intensity) - Moderate to Vigorous Physical Activity

20	randomised trials	not serious	very serious[a]	not serious	not serious	none	6024	5525	-	SMD **0.02 higher** (0.06 lower to 0.11	⊕⊕◯◯ Low[a]	CRITICAL

										higher)		

Physical Activity (Intensity) - Vigorous Physical Activity

7	randomised trials	not serious	very serious[b]	not serious	not serious	publication bias strongly suspected	891	698	-	SMD **0.55 higher** (0.13 higher to 0.97 higher)	⊕◯◯◯ Very low[b]	CRITICAL

Physical Activity (Intensity) - Physical Activity: Counts per minute

13	randomised trials	not serious	very serious[c]	not serious	not serious	none	2910	3262	-	SMD **0.31 higher** (0.08 higher to 0.53	⊕⊕◯◯ Low[c]	CRITICAL

										higher)		

Physical Activity (Intensity) - Physical Activity Steps Per Day

7	randomised trials	serious[d]	very serious[e]	not serious	serious	none	531	592	-	SMD **0.63 higher** (0.23 higher to 1.03 higher)	⊕◯◯◯ Very low[d,e]	CRITICAL

Sedenetary Behavior

8	randomised trials	not serious	not serious	not serious	not serious	none	2491	2266	-	SMD **0.06 lower** (0.12 lower to 0.01 higher)	⊕⊕⊕⊕ High	IMPORTANT

Physical Activity (Intensity) - Moderate to Vigorous Physical Activity

6	randomised trials	not serious	not serious	not serious	not serious	none	294/1259 (23.4%)	201/1192 (16.9%)	**RR 1.20** (1.02 to 1.41)	**34 more per 1,000** (from 3 more to 69 more)	⊕⊕⊕⊕ High	CRITICAL

Physical Activity (Intensity) - Vigorous Physical Activity

1	randomised trials	not serious	not serious	not serious	very serious	none	1/24 (4.2%)	2/27 (7.4%)	**RR 0.56** (0.05 to 5.82)	**33 fewer per 1,000** (from 70 fewer to 357 more)	⊕⊕◯◯ Low	CRITICAL

Physical Activity (Intensity) - Moderate Physical Activity

1	randomised trials	not serious	not serious	not serious	very serious	none	1/24 (4.2%)	2/27 (7.4%)	**RR 0.56** (0.05 to 5.82)	**33 fewer per 1,000** (from 70 fewer to 357 more)	⊕⊕◯◯ Low	CRITICAL

Physical Activity (Intensity) - Light Physical Activity

1	randomised trials	not serious	not serious	not serious	very serious	none	6/24 (25.0%)	5/27 (18.5%)	**RR 1.35** (0.47 to 3.86)	**65 more per 1,000** (from 98 fewer to 530 more)	⊕⊕◯◯ Low	CRITICAL

Sedentary Behavior

4	randomised trials	not serious	not serious	not serious	serious	none	201/410 (49.0 %)	176/302 (58.3 %)	**RR 0.97** (0.85 to 1.10)	**17 fewer per 1,000** (from 87 fewer to 58 more)	⊕⊕⊕◯ Moderate	IMPORTANT

BMI

24	randomised trials	serious[f]	very serious[g]	not serious	not serious	none	5500	4185	-	SMD **0.2 SD higher** (0.04 lower to 0.44 higher)	⊕◯◯◯ Very low[f,g]	IMPORTANT

Physical Fitness - Cardiorespiratory fitness

14	randomised trials	serious[h]	very serious[i]	not serious	not serious	none	3898	3392	-	SMD **0.2 higher** (0.01 higher to 0.39 higher)	⊕◯◯◯ Very low[h,i]	CRITICAL

Physical Fitness - Aerobic Fitness

1	randomised trials	serious[j]	serious[k]	not serious	serious	none	664	483	-	SMD **0.24 lower** (0.43 lower to 0.05 lower)	⊕◯◯◯ Very low[j,k]	CRITICAL

Physical Fitness - V02 max

6	randomised trials	serious[l]	very serious[m]	not serious	serious	none	1060	614	-	SMD **0.98 higher** (0.45 higher to 1.51 higher)	⊕◯◯◯ Very low[l,m]	CRITICAL

5. **CI:** confidence interval; **RR:** risk ratio; **SMD:** standardized mean difference

6. *Explanations*

6. a. Heterogeneity: $Tau^2 = 0.03$; $Chi^2 = 97.62$, df = 25 (P < 0.00001); $I^2 = 74\%$

7. b. Heterogeneity: $Tau^2 = 0.37$; $Chi^2 = 108.71$, df = 9 (P < 0.00001); $I^2 = 92\%$

8. c. Heterogeneity: $Tau^2 = 0.19$; $Chi^2 = 256.43$, df = 15 (P < 0.00001); $I^2 = 94\%$

9. d. The are few some concerns and one high risk

10. e. Heterogeneity: $Tau^2 = 0.36$; $Chi^2 = 83.13$, df = 9 (P < 0.00001); $I^2 = 89\%$

11. f. Most information is from studies at low or unclear risk of bias.

12. g. Heterogeneity: $Tau^2 = 0.41$; $Chi^2 = 874.78$, df = 32 (P < 0.00001); $I^2 = 96\%$

13. h. Most information is from studies at low or unclear risk of bias.

14. i. Heterogeneity: $Tau^2 = 0.11$; $Chi^2 = 145.36$, df = 17 (P < 0.00001); $I^2 = 88\%$

15. j. Most information is from studies at unclear risk of bias.

16. k. Heterogeneity: $Tau^2 = 0.01$; $Chi^2 = 2.59$, df = 1 (P = 0.11); $I^2 = 61\%$

17. l. Most information is from studies at low or unclear risk of bias.

18. m. Heterogeneity: $Tau^2 = 0.54$; $Chi^2 = 93.07$, df = 8 ($P < 0.00001$); $I^2 = 91\%$

Forest Plots & ROB

Study or Subgroup	Experimental Mean	Experimental SD	Experimental Total	Control Mean	Control SD	Control Total	Weight	Std. Mean Difference IV, Random, 95% CI
**2.1.1 Light Physical Activity**								
Aittasalo 2019	165.7	30.7	122	168.8	38.4	118	13.0%	-0.09 [-0.34, 0.16]
carlin 2018	113.11	23.1	52	116.42	21.83	65	6.3%	-0.15 [-0.51, 0.22]
corepal 2019	208.2	66.3	126	192.3	72.9	64	9.2%	0.23 [-0.07, 0.53]
grydeland 2013 (1)	195	45.4549	215	202	44.0454	485	32.3%	-0.16 [-0.32, 0.00]
Okely 2017	239.61	296.1151	306	239.14	314.3798	298	32.8%	0.00 [-0.16, 0.16]
Weigensberg 2021 (2)	21.8	12.6	41	21.4	12.3	13	2.1%	0.03 [-0.59, 0.66]
Weigensberg 2021 (3)	23.5	13.1	34	21.4	12.3	13	2.0%	0.16 [-0.48, 0.80]
Weigensberg 2021 (4)	24.1	14.5	34	21.4	12.3	14	2.1%	0.19 [-0.43, 0.81]
**Subtotal (95% CI)**			**930**			**1070**	**100.0%**	**-0.04 [-0.13, 0.05]**

Heterogeneity: $Tau^2 = 0.00$; $Chi^2 = 6.82$, $df = 7$ ($P = 0.45$); $I^2 = 0\%$
Test for overall effect: $Z = 0.90$ ($P = 0.37$)

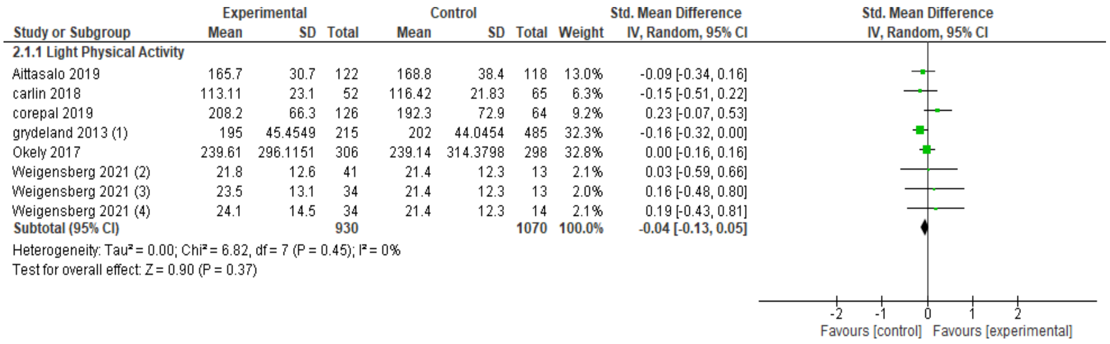

Figure 1: Forest Plot - Light Physical Activity

Unique ID	Study ID	Experimental	Comparator	Outcome	Weight	D1	D2	D3	D4	D5	Overall
1	Aittasalo 2019	RE-AIM intervention	Standard PA	Physical Activity	1	!	+	+	+	+	!
16	carlin 2018	Peer-led brisk walking ses	no intervention	Physical Activity	1	+	+	!	+	+	+
20	Corepal 2019	StepSmart intervention	no intervention	Physical Activity	1	+	+	+	+	+	+
33	Grydeland 2013	HEIA intervention	Standard PA	Physical Activity	1	+	+	+	+	+	+
82	Okeley 2017	PA based intervention	Standard PA	Physical Acitvity	1	+	+	+	+	+	+
128	Weigensberg 2021	Imagery lifestyle interven	none	Physical Acitvity	1	!	+	+	+	!	!

Figure 2: ROB - Light Physical Activity

Study or Subgroup	Experimental Mean	Experimental SD	Experimental Total	Control Mean	Control SD	Control Total	Weight	Std. Mean Difference IV, Random, 95% CI
**2.1.4 Moderate Physical Activity**								
carlin 2018 (5)	17.61	5.62	52	19.84	7.91	65	12.4%	-0.32 [-0.68, 0.05]
corepal 2019 (6)	25.3	13.9	126	29.8	12.8	64	15.2%	-0.33 [-0.63, -0.03]
costigan 2018 (7)	26.37	12.0669	29	26.29	11.8241	18	6.6%	0.01 [-0.58, 0.59]
Okely 2017 (8)	25.65	77.678	306	25	67.0162	298	23.0%	0.01 [-0.15, 0.17]
Peralta 2009 (9)	112	49.5	12	92.9	45.5	11	3.8%	0.39 [-0.44, 1.21]
Sutherland 2016	38.1	19.8662	245	33.4	18.2166	191	21.3%	0.24 [0.05, 0.43]
Weigensberg 2021 (10)	53.1	23.2	34	48	20.7	13	5.8%	0.22 [-0.42, 0.86]
Weigensberg 2021 (11)	53.5	25.2	34	48	20.7	14	6.0%	0.23 [-0.40, 0.85]
Weigensberg 2021 (12)	50	22.9	41	48	20.7	13	6.0%	0.09 [-0.54, 0.71]
**Subtotal (95% CI)**			**879**			**687**	**100.0%**	**0.01 [-0.16, 0.18]**

Heterogeneity: $Tau^2 = 0.03$; $Chi^2 = 15.36$, $df = 8$ ($P = 0.05$); $I^2 = 48\%$
Test for overall effect: $Z = 0.12$ ($P = 0.90$)

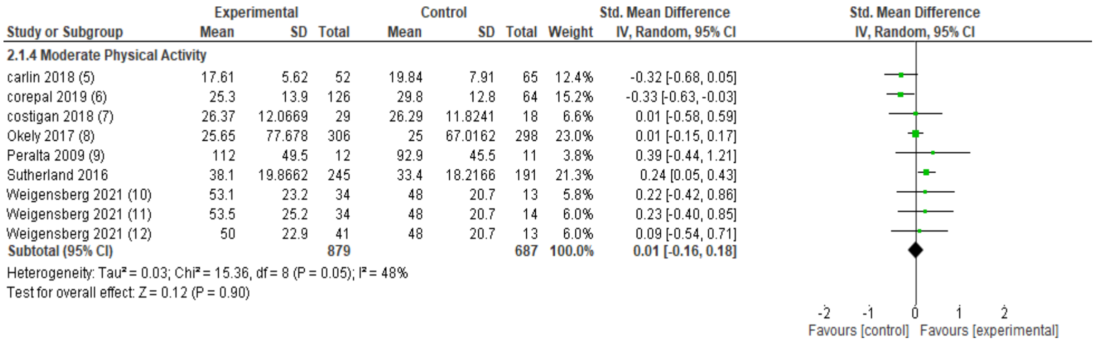

Figure 3: Forest Plot - Moderate Physical Activity

Unique ID	Study ID	Experimental	Comparator	Outcome	Weight	D1	D2	D3	D4	D5	Overall
16	carlin 2018	Peer-led brisk walking ses	no intervention	Physical Activity	1	+	+	!	+	+	+
20	Corepal 2019	StepSmart intervention	no intervention	Physical Activity	1	+	+	+	+	+	+
23	Costigan 2018	HIIT sessions	Standard PA	Physical Activity	1	+	+	+	+	+	+
82	Okeley 2017	PA based intervention	Standard PA	Physical Acitvity	1	+	+	+	+	+	+
87	Peralta 2009	FILA program	Standard PA	Physical Activity	1	+	+	+	+	+	+
109	Sutherland 2016	PAE41 intervention	Standard PA	Physical Acitvity	1	+	+	+	+	+	+
128	Weigensberg 2021	Imagery lifestyle interven	none	Physical Acitvity	1	!	+	+	+	!	!

Figure 3: ROB - Moderate Physical Activity

Unique ID	Study ID	Experimental	Comparator	Outcome	Weight	D1	D2	D3	D4	D5	Overall
1	Aittasalo 2019	RE-AIM intervention	Standard PA	Physical Activity	1	!	+	+	+	+	!
18	Corder 2016	GoActive intervention	standard	Physical Activity	1	+	+	+	+	!	+
19	Corder 2020	GoActive Intervention	standard curriculum	Physical Activity	1	+	+	+	+	+	+
20	Corepal 2019	StepSmart intervention	no intervention	Physical Activity	1	+	+	+	+	+	+
33	Grydeland 2013	HEIA intervention	Standard PA	Physical Activity	1	+	+	+	+	+	+
38	Ha 2019	SELF FIT intervention	wait list	Physical Activity	1	+	+	+	+	+	+
40	Hankonen 2017	multi-level intervention	usual	Physical Activity	1	+	!	+	+	!	!
48	jago 2015	after-school dance progra	no intervention	Physical Activity	1	+	+	+	+	+	+
50	Jago 2021	PLAN A intervention	standard curriculum	Physical Activity	1	+	+	+	+	+	+
58	Kolle 2020	PAL intervention	Standard PA	Physical Activity	1	+	+	+	+	+	+
59	Kolle 2020	PAL intervention	Standard PA	Physical Activity	1	+	+	+	+	+	+
72	Lubans 2012	multicomponent school-t	regular school curriculum	Physical Activity	1	+	+	!	+	+	+
82	Okeley 2017	PA based intervention	Standard PA	Physical Activity	1	+	+	+	+	+	+
87	Peralta 2009	FILA program	Standard PA	Physical Activity	1	+	+	+	+	+	+
93	Robbins 2019	Girls on the move interve	none	Physical Activity	1	!	+	+	+	+	!
95	Sebire 2018	PLAN A intervention	none	Physical Activity	1	+	+	+	+	+	+
109	Sutherland 2016	PAE41 intervention	Standard PA	Physical Activity	1	+	+	+	+	+	+
113	telford 2016	Specialist-Taught Physical	usual PE	Physical Activity	1	+	+	+	!	+	+
115	Toftager 2014	SPACE study	none	Physical Activity	1	+	+	+	+	+	+
128	Weigensberg 2021	Imagery lifestyle interven	none	Physical Activity	1	!	+	+	+	!	!
132	Zarrett 2021	Connect through PLAY int	wait list	Physical Activity	1	!	!	+	+	!	!

Figure 3: ROB - Moderate to Vigorous Physical Activity

Study or Subgroup	Experimental Mean	Experimental SD	Experimental Total	Control Mean	Control SD	Control Total	Weight	Mean Difference IV, Random, 95% CI
**2.15.14 Physical Activity: Counts per minute**								
Aittasalo 2019 (1)	239.3	49.7	122	239.2	60.6	118	7.7%	0.10 [-13.95, 14.15]
Bergh 2012 (2)	569.8	251.6	215	563.8	255.3	485	5.6%	6.00 [-34.59, 46.59]
carlin 2018 (3)	134.69	24.28	52	142.67	28.64	65	7.9%	-7.98 [-17.57, 1.61]
Dudley 2010 (4)	82.1	17.6	17	75	37	21	7.5%	7.10 [-10.80, 25.00]
grydeland 2013	570	252	215	564	255	485	5.6%	6.00 [-34.62, 46.62]
ha 2019	80,033	31,886	311	64,101	28,538	354	0.0%	15932.00 [11306.40, 20557.60]
jago 2015 (5)	450.98	263.89	124	405.04	228.96	145	4.1%	45.94 [-13.61, 105.49]
jago 2015 (6)	478.75	144.94	246	446.83	137.81	262	7.0%	31.92 [7.29, 56.55]
Kolle 2020 (7)	497	298.6269	317	489	206.4054	144	5.1%	8.00 [-39.09, 55.09]
Kolle 2020 (8)	476	284.158	229	234	36.4245	144	5.9%	242.00 [204.72, 279.28]
lubans 2012 (9)	328.4	91.5	141	359.2	140.4	153	6.8%	-30.80 [-57.69, -3.91]
miller 2015 (10)	19.04	12.2554	97	0.78	12.3365	71	8.1%	18.26 [14.49, 22.03]
smith 2014 (11)	515	33.51	120	473	28.58	120	8.0%	42.00 [34.12, 49.88]
smith 2014 (12)	410	54.85	83	387	47.13	83	7.6%	23.00 [7.44, 38.56]
Sutherland 2016	460.2	174.8228	245	448.5	165.3505	191	6.3%	11.70 [-20.38, 43.78]
toftager 2014 (13)	408	167	376	433	206	421	6.9%	-25.00 [-50.93, 0.93]
**Subtotal (95% CI)**			**2910**			**3262**	**100.0%**	**23.11 [5.78, 40.44]**
Heterogeneity: Tau² = 964.99; Chi² = 282.22, df = 15 (P < 0.00001); I² = 95%								
Test for overall effect: Z = 2.61 (P = 0.009)								
**Total (95% CI)**			**2910**			**3262**	**100.0%**	**23.11 [5.78, 40.44]**
Heterogeneity: Tau² = 964.99; Chi² = 282.22, df = 15 (P < 0.00001); I² = 95%								
Test for overall effect: Z = 2.61 (P = 0.009)								
Test for subgroup differences: Not applicable								

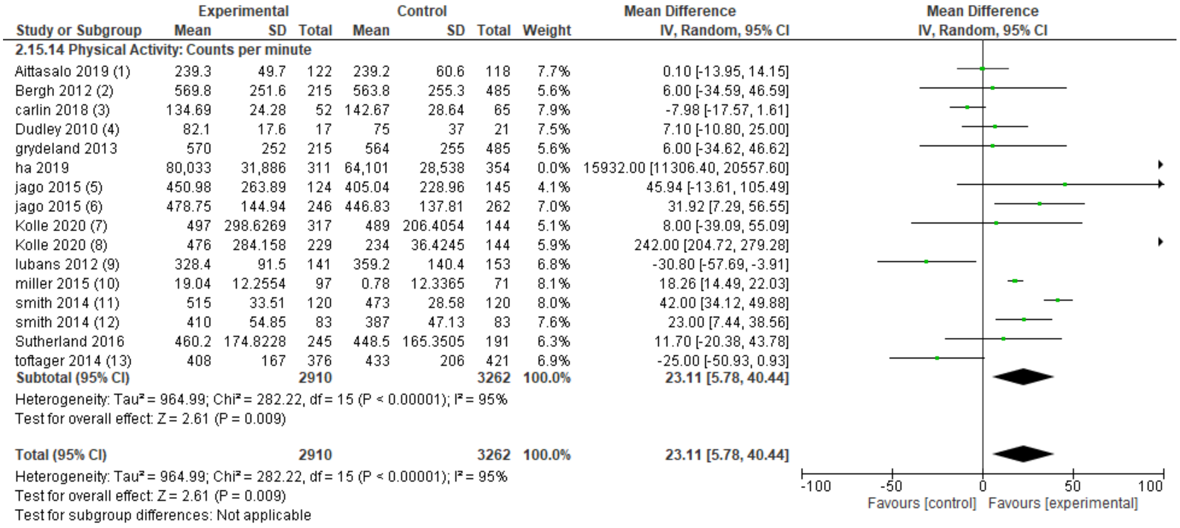

Figure 4: Forest Plot - Counts per minute

Unique ID	Study ID	Experimental	Comparator	Outcome	Weight	D1	D2	D3	D4	D5	Overall
1	Aittasalo 2019	RE-AIM intervention	Standard PA	Physical Activity-CPM	1	!	+	+	+	+	!
12	Bergh 2012	multi component interve	no intervention	Physical Activity-CPM	1	!	!	+	+	!	!
16	carlin 2018	Peer-led brisk walking ses	no intervention	Physical Activity-CPM	1	+	+	!	+	+	+
28	Dudley 2010	school based exercise int	Standard PA	Physical Activity-CPM	1	+	+	+	+	+	+
33	Grydeland 2013	HEIA intervention	Standard PA	Physical Activity-CPM	1	+	+	+	+	+	+
38	Ha 2019	SELF FIT intervention	wait list	Physical Activity-CPM	1	+	+	+	+	+	+
48	jago 2015	after-school dance progra	no intervention	Physical Activity-CPM	1	+	+	+	+	+	+
58	Kolle 2020	PAL intervention	Standard PA	Physical Activity-CPM	1	+	+	+	+	+	+
59	Kolle 2020	PAL intervention	Standard PA	Physical Activity-CPM	1	+	+	+	+	+	+
72	Lubans 2012	multicomponent school-b	regular school curriculum	Physical Activity-CPM	1	+	+	!	+	+	+
78	Miller 2015	PLUNGE program interve	none	Physical Activity-CPM	1	+	+	+	+	+	+
105	Smith 2014	ATLAS intervention	regualr school curriculum	Physical Activity-CPM	1	+	+	+	+	+	+
109	Sutherland 2016	PAE41 intervention	Standard PA	Physical Activity-CPM	1	+	+	+	+	+	+
115	Toftager 2014	SPACE study	none	Physical Activity-CPM	1	+	+	+	+	+	+

Figure 5: ROB - Counts per minute

Study or Subgroup	Experimental Mean	Experimental SD	Experimental Total	Control Mean	Control SD	Control Total	Weight	Mean Difference IV, Random, 95% CI
**2.14.11 Physical Activity Steps Per Day**								
Aittasalo 2019	9,292	2,704	122	9,141	3,200	118	12.1%	151.00 [-599.72, 901.72]
corepal 2019 (1)	7,374.7	2,864.5	126	8,725.6	4,943.7	229	12.0%	-1350.90 [-2163.39, -538.41]
lubans 2009 (2)	14,158	3,375	26	8,728	3,603	17	10.1%	5430.00 [3281.42, 7578.58]
lubans 2009 (3)	11,880	2,314	24	8,309	2,400	35	11.5%	3571.00 [2350.65, 4791.35]
lubans 2010 (4)	14,134	4,230	30	8,984	3,652	23	10.1%	5150.00 [3024.27, 7275.73]
lubans 2010 (5)	11,402	2,549	28	8,916	2,621	43	11.5%	2486.00 [1259.17, 3712.83]
lubans 2012	11,948	4,402	50	11,886	3,993	50	10.9%	62.00 [-1585.34, 1709.34]
Shimon 2009 (6)	14,827	4,444	43	11,571	3,260	18	10.3%	3256.00 [1247.92, 5264.08]
Shimon 2009 (7)	13,798	5,003	34	11,571	3,260	18	9.9%	2227.00 [-30.45, 4484.45]
White 2022	72,922.73	32,364.01	48	58,526.15	21,402.43	41	1.7%	14396.58 [3138.51, 25654.65]
**Subtotal (95% CI)**			**531**			**592**	**100.0%**	**2424.05 [854.76, 3993.34]**
Heterogeneity: Tau² = 5172772.32; Chi² = 103.40, df = 9 (P < 0.00001); I² = 91%								
Test for overall effect: Z = 3.03 (P = 0.002)								
**Total (95% CI)**			**531**			**592**	**100.0%**	**2424.05 [854.76, 3993.34]**
Heterogeneity: Tau² = 5172772.32; Chi² = 103.40, df = 9 (P < 0.00001); I² = 91%								
Test for overall effect: Z = 3.03 (P = 0.002)								

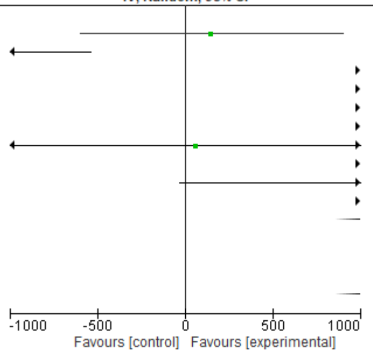

Figure 6: Forest Plot - Steps per say

Unique ID	Study ID	Experimental	Comparator	Outcome	Weight	D1	D2	D3	D4	D5	Overall
1	Aittasalo 2019	RE-AIM intervention	Standard PA	Physical Activity -Steps	1	!	+	+	+	+	!
20	Corepal 2019	StepSmart intervention	no intervention	Physical Activity -Steps	1	+	+	+	+	+	+
67	Luban 2009	Extracurricular school spc	Standard PA	Physical Activity -Steps	1	+	+	+	+	+	+
68	luban 2010	Program X intervention	no behavioural support w	Physical Activity -Steps	1	+	+	+	+	+	+
70	lubans 2010	PALs intervention	none	Physical Activity -Steps	1	!	+	+	+	+	!
72	Lubans 2012	multicomponent school-b	regular school curriculum	Physical Activity -Steps	1	+	+	!	+	+	+
99	shimon 2009	plotting step count	no feedback on stepcount	Physical Activity -Steps	1	!	+	+	+	!	!
129	White 2022	HWBG intervention	wait list control	Physical Activity -Steps	1	+	-	+	+	!	-

Figure 7: ROB: Steps per day

Study or Subgroup	Experimental Mean	Experimental SD	Experimental Total	Control Mean	Control SD	Control Total	Weight	Std. Mean Difference IV, Random, 95% CI
**2.2.1 0-6 months**								
carlin 2018 (1)	113.11	23.1	52	116.42	21.83	65	2.8%	-0.15 [-0.51, 0.22]
carlin 2018 (2)	17.61	5.62	52	19.84	7.91	65	2.8%	-0.32 [-0.68, 0.05]
carlin 2018 (3)	3.97	3.15	52	6.42	6.05	65	2.8%	-0.49 [-0.86, -0.12]
carlin 2018 (4)	134.69	24.28	52	142.67	28.64	65	2.8%	-0.30 [-0.66, 0.07]
Corder 2016 (5)	49.4	18.2	285	42.1	15	115	3.1%	0.42 [0.20, 0.64]
costigan 2018 (6)	26.37	12.0669	29	26.29	11.8241	18	2.4%	0.01 [-0.58, 0.59]
costigan 2018 (7)	9.76	6.2306	29	8.73	6.093	18	2.4%	0.16 [-0.43, 0.75]
Dudley 2010 (8)	82.1	17.6	17	75	37	21	2.3%	0.23 [-0.41, 0.87]
ha 2019 (9)	80,033	31,886	311	64,101	28,538	354	3.2%	0.53 [0.37, 0.68]
ha 2019 (10)	10.8	4.68	311	8.87	4.01	354	3.2%	0.44 [0.29, 0.60]
hankonen 2017 (11)	0.07	0.03	12	0.08	0.06	3	1.2%	-0.26 [-1.53, 1.01]
jago 2021 (12)	45.19	18.43	603	48.89	20.85	616	3.2%	-0.19 [-0.30, -0.08]
lubans 2009 (13)	11,880	2,314	24	8,309	2,400	35	2.4%	1.49 [0.90, 2.08]
lubans 2009 (14)	14,158	3,375	26	8,728	3,603	17	2.1%	1.54 [0.84, 2.24]
lubans 2010 (15)	14,134	4,230	30	8,984	3,652	23	2.4%	1.27 [0.67, 1.87]
lubans 2010 (16)	11,402	2,549	28	8,916	2,621	43	2.6%	0.95 [0.45, 1.45]
lubans 2012 (17)	22.1	9.73	141	27.7	18.8	153	3.1%	-0.37 [-0.60, -0.14]
miller 2015 (18)	19.04	12.2554	97	0.78	12.3365	71	2.9%	1.48 [1.13, 1.82]
Peralta 2009 (19)	112	49.5	12	92.9	45.5	11	1.9%	0.39 [-0.44, 1.21]
Peralta 2009 (20)	3.7	4.9	12	6.9	5.7	11	1.9%	-0.58 [-1.42, 0.26]
Peralta 2009 (21)	11.3	15.4	12	3.7	4.8	11	1.9%	0.63 [-0.21, 1.47]
Peralta 2009 (22)	53.8	42.1	12	70.9	53.6	11	1.9%	-0.34 [-1.17, 0.48]
Peralta 2009 (23)	123.3	62	12	96.6	49.8	11	1.9%	0.46 [-0.38, 1.29]
sebire 2018 (24)	46.57	37.52	74	37.95	32.14	35	2.8%	0.24 [-0.16, 0.64]
sebire 2018 (25)	58.65	22.21	162	54.31	19.56	81	3.0%	0.20 [-0.06, 0.47]
Shimon 2009 (26)	14,827	4,444	43	11,571	3,260	18	2.4%	0.78 [0.21, 1.35]
Shimon 2009 (27)	13,798	5,003	34	11,571	3,260	18	2.4%	0.49 [-0.09, 1.07]
Weigensberg 2021 (28)	21.8	12.6	41	21.4	12.3	13	2.3%	0.03 [-0.59, 0.66]
Weigensberg 2021 (29)	23.5	13.1	34	21.4	12.3	13	2.3%	0.16 [-0.48, 0.80]
Weigensberg 2021 (30)	24.1	14.5	34	21.4	12.3	14	2.3%	0.19 [-0.43, 0.81]
Weigensberg 2021 (31)	289.4	117.2	41	273.6	108.4	13	2.3%	0.14 [-0.49, 0.76]
Weigensberg 2021 (32)	296	112.8	34	273.6	108.4	13	2.3%	0.20 [-0.44, 0.84]
Weigensberg 2021 (33)	306	130.6	34	273.6	108.4	14	2.3%	0.26 [-0.37, 0.88]
Weigensberg 2021 (34)	239.3	95.6	41	48	20.7	13	2.0%	2.23 [1.47, 2.99]
Weigensberg 2021 (35)	50	22.9	41	48	20.7	13	2.3%	0.09 [-0.54, 0.71]
Weigensberg 2021 (36)	243	92.4	34	48	20.7	13	1.9%	2.40 [1.59, 3.22]
Weigensberg 2021 (37)	53.1	23.2	34	48	20.7	13	2.3%	0.22 [-0.42, 0.86]
Weigensberg 2021 (38)	252.5	107.2	34	48	20.7	14	2.0%	2.20 [1.43, 2.97]
Weigensberg 2021 (39)	53.5	25.2	34	48	20.7	14	2.3%	0.23 [-0.40, 0.85]
White 2022 (40)	72,922.73	32,364.01	48	58,526.15	21,402.43	41	2.7%	0.51 [0.09, 0.94]
zarrett 2021 (41)	44.32	23.57	92	37.53	22.33	75	3.0%	0.29 [-0.01, 0.60]
**Subtotal (95% CI)**			**3100**			**2584**	**100.0%**	**0.41 [0.23, 0.59]**

Heterogeneity: Tau² = 0.25; Chi² = 332.59, df = 40 (P < 0.00001); I² = 88%
Test for overall effect: Z = 4.54 (P < 0.00001)

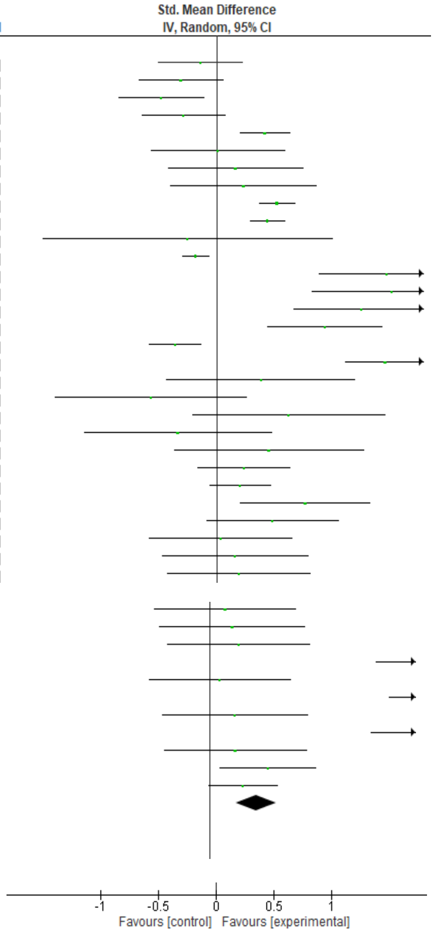

Figure 8:Forest Plot - Physical Activity Subgroup Analysis 0 -6 Month follow up

Study or Subgroup	Experimental Mean	Experimental SD	Experimental Total	Control Mean	Control SD	Control Total	Weight	Std. Mean Difference IV, Random, 95% CI
**2.2.3 7-12 months**								
Aittasalo 2019 (42)	165.7	30.7	122	168.8	38.4	118	6.2%	-0.09 [-0.34, 0.16]
Aittasalo 2019 (43)	239.3	49.7	122	239.2	60.6	118	6.2%	0.00 [-0.25, 0.25]
Aittasalo 2019 (44)	73.6	28	122	70.4	31.4	118	6.2%	0.11 [-0.15, 0.36]
Aittasalo 2019 (45)	9,292	2,704	122	9,141	3,200	118	6.2%	0.05 [-0.20, 0.30]
corder 2020 (46)	25.6	21.5	1003	27.6	20.6	871	7.4%	-0.09 [-0.19, -0.00]
corepal 2019 (47)	7,374.7	2,864.5	126	8,725.6	4,943.7	229	6.5%	-0.31 [-0.53, -0.09]
corepal 2019 (48)	33.9	22.5	126	38.9	20.1	64	5.7%	-0.23 [-0.53, 0.07]
corepal 2019 (49)	208.2	66.3	126	192.3	72.9	64	5.7%	0.23 [-0.07, 0.53]
corepal 2019 (50)	25.3	13.9	126	29.8	12.8	64	5.7%	-0.33 [-0.63, -0.03]
corepal 2019 (51)	7	8.1	126	8.5	7.05	64	5.7%	-0.19 [-0.49, 0.11]
jago 2015 (52)	478.75	144.94	246	446.83	137.81	262	6.9%	0.23 [0.05, 0.40]
jago 2015 (53)	56.55	21.92	246	57.69	19.61	262	6.9%	-0.05 [-0.23, 0.12]
lubans 2012 (54)	11,948	4,402	50	11,886	3,993	50	4.9%	0.01 [-0.38, 0.41]
lubans 2012 (55)	328.4	91.5	141	359.2	140.4	153	6.4%	-0.26 [-0.49, -0.03]
Robbins 2019 (56)	2.59	1.19	753	2.64	1.15	766	7.3%	-0.04 [-0.14, 0.06]
smith 2014 (57)	515	33.51	120	473	28.58	120	5.9%	1.34 [1.06, 1.62]
**Subtotal (95% CI)**			**3677**			**3441**	**100.0%**	**0.02 [-0.13, 0.17]**

Heterogeneity: Tau² = 0.07; Chi² = 123.75, df = 15 (P < 0.00001); I² = 88%
Test for overall effect: Z = 0.27 (P = 0.79)

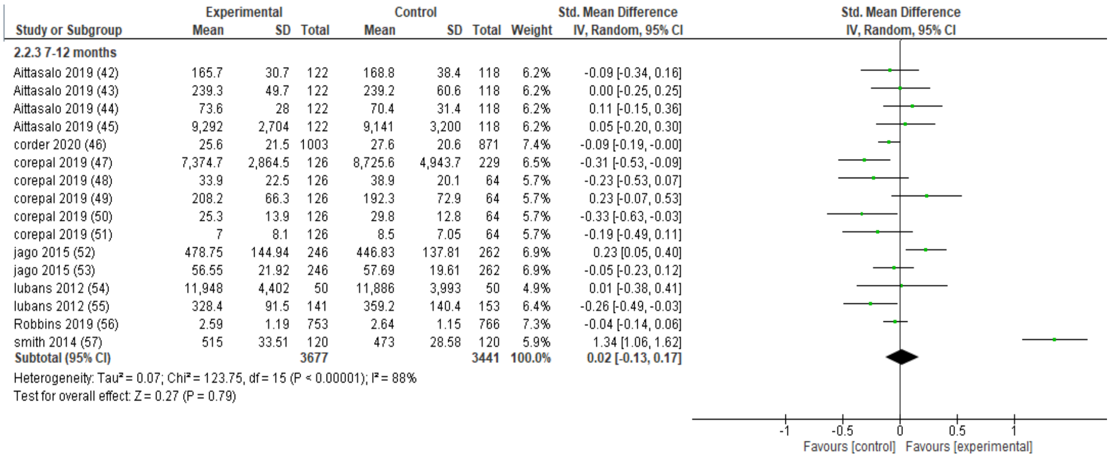

Figure 9: Forest Plot - Physical Activity Subgroup Analysis 7 -12 Months follow up

Study or Subgroup	Experimental Mean	Experimental SD	Experimental Total	Control Mean	Control SD	Control Total	Weight	Std. Mean Difference IV, Random, 95% CI
**2.2.5 13-20months**								
Bergh 2012 (58)	569.8	251.6	215	563.8	255.3	485	8.5%	0.02 [-0.14, 0.18]
grydeland 2013 (59)	570	252	215	564	255	485	8.5%	0.02 [-0.14, 0.18]
grydeland 2013 (60)	67	29.3258	215	71	28.6295	485	8.5%	-0.14 [-0.30, 0.02]
grydeland 2013 (61)	195	45.4549	215	202	44.0454	485	8.5%	-0.16 [-0.32, 0.00]
Kolle 2020 (62)	497	298.6269	317	489	206.4054	144	8.1%	0.03 [-0.17, 0.23]
Kolle 2020 (63)	476	284.158	229	234	36.4245	144	7.8%	1.08 [0.86, 1.30]
Kolle 2020 (64)	65	45.2465	317	65	30.3537	144	8.1%	0.00 [-0.20, 0.20]
Kolle 2020 (65)	63	46.0797	229	65	30.3537	144	8.0%	-0.05 [-0.26, 0.16]
Okely 2017 (66)	27.13	83.7407	306	27	76.7529	298	8.5%	0.00 [-0.16, 0.16]
Okely 2017 (67)	25.65	77.678	306	25	67.0162	298	8.5%	0.01 [-0.15, 0.17]
Okely 2017 (68)	1.33	7.5562	306	1.5	8.7718	298	8.5%	-0.02 [-0.18, 0.14]
Okely 2017 (69)	239.61	296.1151	306	239.14	314.3798	298	8.5%	0.00 [-0.16, 0.16]
**Subtotal (95% CI)**			**3176**			**3708**	**100.0%**	**0.06 [-0.09, 0.21]**

Heterogeneity: Tau² = 0.06; Chi² = 96.10, df = 11 (P < 0.00001); I² = 89%
Test for overall effect: Z = 0.81 (P = 0.42)

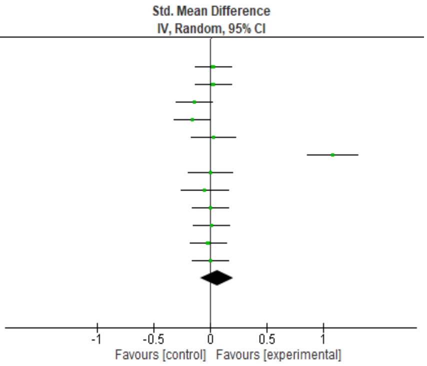

Figure 10: Forest Plot - Physical Activity Subgroup Analysis 13 - 20 Months follow up

Study or Subgroup	Experimental Mean	Experimental SD	Experimental Total	Control Mean	Control SD	Control Total	Weight	Std. Mean Difference IV, Random, 95% CI
**2.2.8 21-24 months**								
Sutherland 2016 (70)	57.9	34.1699	245	50.8	31.5287	191	15.9%	0.21 [0.02, 0.40]
Sutherland 2016 (71)	460.2	174.8228	245	448.5	165.3505	191	15.9%	0.07 [-0.12, 0.26]
Sutherland 2016 (72)	38.1	19.8662	245	33.4	18.2166	191	15.9%	0.24 [0.05, 0.43]
Sutherland 2016 (73)	19.7	17.4823	245	17.4	16.8153	191	15.9%	0.13 [-0.06, 0.32]
toftager 2014 (74)	44	23	376	48	27	421	18.2%	-0.16 [-0.30, -0.02]
toftager 2014 (75)	408	167	376	433	206	421	18.2%	-0.13 [-0.27, 0.01]
**Subtotal (95% CI)**			**1732**			**1606**	**100.0%**	**0.05 [-0.09, 0.20]**

Heterogeneity: Tau² = 0.03; Chi² = 21.96, df = 5 (P = 0.0005); I² = 77%
Test for overall effect: Z = 0.71 (P = 0.48)

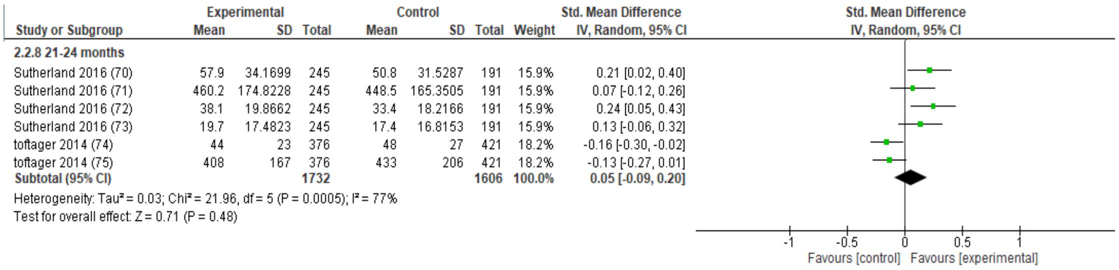

Figure 11: Forest Plot - Physical Activity Subgroup Analysis 21 - 24 Months follow up

Study or Subgroup	Experimental Mean	Experimental SD	Experimental Total	Control Mean	Control SD	Control Total	Weight	Std. Mean Difference IV, Random, 95% CI
**2.2.9 4 years**								
telford 2016 (76)	49.9	24.4466	139	48.7	26.1951	127	49.2%	0.05 [-0.19, 0.29]
telford 2016 (77)	34	22.8244	134	35.6	22.7406	140	50.8%	-0.07 [-0.31, 0.17]
**Subtotal (95% CI)**			**273**			**267**	**100.0%**	**-0.01 [-0.18, 0.16]**

Heterogeneity: Tau² = 0.00; Chi² = 0.46, df = 1 (P = 0.50); I² = 0%
Test for overall effect: Z = 0.14 (P = 0.89)

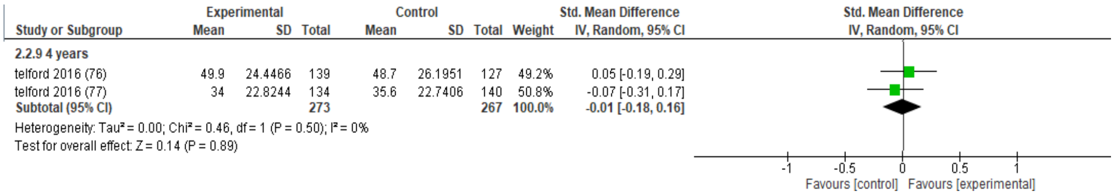

Figure 12: Forest Plot - Physical Activity Subgroup Analysis 4 years follow up

Study or Subgroup	Experimental Mean	Experimental SD	Experimental Total	Control Mean	Control SD	Control Total	Weight	Std. Mean Difference IV, Random, 95% CI
**2.2.1 Multi component Intervention**								
Dudley 2010 (1)	82.1	17.6	17	75	37	21	1.0%	0.23 [-0.41, 0.87]
grydeland 2013 (2)	195	45.4549	215	202	44.0454	485	1.8%	-0.16 [-0.32, 0.00]
grydeland 2013 (3)	67	29.3258	215	71	28.6295	485	1.8%	-0.14 [-0.30, 0.02]
Kolle 2020 (4)	63	46.0797	229	65	30.3537	144	1.7%	-0.05 [-0.26, 0.16]
lubans 2010 (5)	14,134	4,230	30	8,984	3,652	23	1.1%	1.27 [0.67, 1.87]
lubans 2010 (6)	11,402	2,549	28	8,916	2,621	43	1.2%	0.95 [0.45, 1.45]
lubans 2010 (7)	14,134	4,230	30	8,984	3,652	23	1.1%	1.27 [0.67, 1.87]
lubans 2010 (8)	11,402	2,549	28	8,916	2,621	43	1.2%	0.95 [0.45, 1.45]
lubans 2012 (9)	328.4	91.5	141	359.2	140.4	153	1.7%	-0.26 [-0.49, -0.03]
Peralta 2009 (10)	11.3	15.4	12	3.7	4.8	11	0.8%	0.63 [-0.21, 1.47]
Peralta 2009 (11)	112	49.5	12	92.9	45.5	11	0.8%	0.39 [-0.44, 1.21]
Peralta 2009 (12)	123.3	62	12	96.6	49.8	11	0.8%	0.46 [-0.38, 1.29]
smith 2014 (13)	515	33.51	120	473	28.58	120	1.6%	1.34 [1.06, 1.62]
Sutherland 2016 (14)	57.9	34.1699	245	50.8	31.5287	191	1.7%	0.21 [0.02, 0.40]
Sutherland 2016	460.2	174.8228	245	448.5	165.3505	191	1.7%	0.07 [-0.12, 0.26]
Sutherland 2016 (15)	19.7	17.4823	245	17.4	16.8153	191	1.7%	0.13 [-0.06, 0.32]
toftager 2014 (16)	408	167	376	433	206	421	1.8%	-0.13 [-0.27, 0.01]
toftager 2014 (17)	44	23	376	48	27	421	1.8%	-0.16 [-0.30, -0.02]
Weigensberg 2021 (18)	296	112.8	34	273.6	108.4	13	1.0%	0.20 [-0.44, 0.84]
Weigensberg 2021 (19)	306	130.6	34	273.6	108.4	14	1.0%	0.26 [-0.37, 0.88]
Weigensberg 2021 (20)	289.4	117.2	41	273.6	108.4	13	1.0%	0.14 [-0.49, 0.76]
Weigensberg 2021 (21)	252.5	107.2	34	48	20.7	14	0.8%	2.20 [1.43, 2.97]
Weigensberg 2021 (22)	239.3	95.6	41	48	20.7	13	0.8%	2.23 [1.47, 2.99]
Weigensberg 2021 (23)	243	92.4	34	48	20.7	13	0.8%	2.40 [1.59, 3.22]
Weigensberg 2021 (24)	21.8	12.6	41	21.4	12.3	13	1.0%	0.03 [-0.59, 0.66]
Weigensberg 2021 (25)	23.5	13.1	34	21.4	12.3	13	1.0%	0.16 [-0.48, 0.80]
Weigensberg 2021 (26)	24.1	14.5	34	21.4	12.3	14	1.0%	0.19 [-0.43, 0.81]
White 2022	72,922.73	32,364.01	48	58,526.15	21,402.43	41	1.4%	0.51 [0.09, 0.94]
**Subtotal (95% CI)**			**2951**			**3149**	**35.2%**	**0.46 [0.27, 0.65]**
Heterogeneity: $Tau^2 = 0.20$; $Chi^2 = 277.48$, df = 27 (P < 0.00001); $I^2 = 90\%$								
Test for overall effect: Z = 4.74 (P < 0.00001)								
**2.2.2 Physical Activity/Education Lessons**								
carlin 2018 (27)	3.97	3.15	52	6.42	6.05	65	1.5%	-0.49 [-0.86, -0.12]
carlin 2018 (28)	113.11	23.1	52	116.42	21.83	65	1.5%	-0.15 [-0.51, 0.22]
carlin 2018 (29)	17.61	5.62	52	19.84	7.91	65	1.5%	-0.32 [-0.68, 0.05]
carlin 2018 (30)	134.69	24.28	52	142.67	28.64	65	1.5%	-0.30 [-0.66, 0.07]
Corder 2016 (31)	49.4	18.2	285	42.1	15	115	1.7%	0.42 [0.20, 0.64]
corder 2020 (32)	25.6	21.5	1003	27.6	20.6	871	1.8%	-0.09 [-0.19, -0.00]
corepal 2019 (33)	7,374.7	2,864.5	126	8,725.6	4,943.7	229	1.7%	-0.31 [-0.53, -0.09]
corepal 2019 (34)	208.2	66.3	126	192.3	72.9	64	1.6%	0.23 [-0.07, 0.53]
corepal 2019 (35)	25.3	13.9	126	29.8	12.8	64	1.6%	-0.33 [-0.63, -0.03]
corepal 2019 (36)	33.9	22.5	126	38.9	20.1	64	1.6%	-0.23 [-0.53, 0.07]
costigan 2018 (37)	9.76	6.2306	29	8.73	6.093	18	1.1%	0.16 [-0.43, 0.75]
costigan 2018 (38)	26.37	12.0669	29	26.29	11.8241	18	1.1%	0.01 [-0.58, 0.59]
Dudley 2010 (39)	82.1	17.6	17	75	37	21	1.0%	0.23 [-0.41, 0.87]
ha 2019 (40)	10.8	4.68	311	8.87	4.01	354	1.8%	0.44 [0.29, 0.60]
ha 2019	80,033	31,886	311	64,101	28,538	354	1.8%	0.53 [0.37, 0.68]
jago 2021 (41)	45.19	18.43	603	48.89	20.85	616	1.8%	-0.19 [-0.30, -0.08]
Kolle 2020 (42)	65	45.2465	317	65	30.3537	144	1.7%	0.00 [-0.20, 0.20]
Kolle 2020 (43)	63	46.0797	229	65	30.3537	144	1.7%	-0.05 [-0.26, 0.16]
Kolle 2020 (44)	476	284.158	229	234	36.4245	144	1.7%	1.08 [0.86, 1.30]
Kolle 2020 (45)	497	298.6269	317	489	206.4054	144	1.7%	0.03 [-0.17, 0.23]
miller 2015 (46)	19.04	12.2554	97	0.78	12.3365	71	1.5%	1.48 [1.13, 1.82]
Okely 2017 (47)	1.33	7.5562	306	1.5	8.7718	298	1.8%	-0.02 [-0.18, 0.14]
Okely 2017 (48)	239.61	296.1151	306	239.14	314.3798	298	1.8%	0.00 [-0.16, 0.16]
Okely 2017 (49)	25.65	77.678	306	25	67.0162	298	1.8%	0.01 [-0.15, 0.17]
Okely 2017 (50)	27.13	83.7407	306	27	76.7529	298	1.8%	0.00 [-0.16, 0.16]
Peralta 2009 (51)	123.3	62	12	96.6	49.8	11	0.8%	0.46 [-0.38, 1.29]
telford 2016 (52)	49.9	24.4466	139	48.7	26.1951	127	1.7%	0.05 [-0.19, 0.29]
telford 2016 (53)	34	22.8244	134	35.6	22.7406	140	1.7%	-0.07 [-0.31, 0.17]
Weigensberg 2021 (54)	296	112.8	34	273.6	108.4	13	1.0%	0.20 [-0.44, 0.84]
Weigensberg 2021 (55)	306	130.6	34	273.6	108.4	14	1.0%	0.26 [-0.37, 0.88]
Weigensberg 2021 (56)	289.4	117.2	41	273.6	108.4	13	1.0%	0.14 [-0.49, 0.76]
**Subtotal (95% CI)**			**6107**			**5205**	**47.0%**	**0.09 [-0.04, 0.22]**
Heterogeneity: $Tau^2 = 0.10$; $Chi^2 = 291.07$, df = 30 (P < 0.00001); $I^2 = 90\%$								
Test for overall effect: Z = 1.42 (P = 0.16)								
**2.2.3 Educational Awareness Lessons**								
Aittasalo 2019 (57)	239.3	49.7	122	239.2	60.6	118	1.6%	0.00 [-0.25, 0.25]
Aittasalo 2019	9,292	2,704	122	9,141	3,200	118	1.6%	0.05 [-0.20, 0.30]
Aittasalo 2019 (58)	239.3	49.7	122	239.2	60.6	118	1.6%	0.00 [-0.25, 0.25]
Aittasalo 2019 (59)	165.7	30.7	122	168.8	38.4	118	1.6%	-0.09 [-0.34, 0.16]
Aittasalo 2019 (60)	73.6	28	122	70.4	31.4	118	1.6%	0.11 [-0.15, 0.36]
ha 2019 (61)	10.8	4.68	311	8.87	4.01	354	1.8%	0.44 [0.29, 0.60]
lubans 2009 (62)	14,158	3,375	26	8,728	3,603	17	0.9%	1.54 [0.84, 2.24]
lubans 2009 (63)	11,880	2,314	24	8,309	2,400	35	1.1%	1.49 [0.90, 2.08]
Shimon 2009 (64)	14,827	4,444	43	11,571	3,260	18	1.1%	0.78 [0.21, 1.35]
Shimon 2009 (65)	13,798	5,003	34	11,571	3,260	18	1.1%	0.49 [-0.09, 1.07]
**Subtotal (95% CI)**			**1048**			**1032**	**14.3%**	**0.37 [0.13, 0.62]**
Heterogeneity: $Tau^2 = 0.12$; $Chi^2 = 57.87$, df = 9 (P < 0.00001); $I^2 = 84\%$								
Test for overall effect: Z = 2.97 (P = 0.003)								
**2.2.4 After School Activity**								
ha 2019 (66)	10.8	4.68	311	8.87	4.01	354	1.8%	0.44 [0.29, 0.60]
jago 2015 (67)	478.75	144.94	246	446.83	137.81	262	1.8%	0.23 [0.05, 0.40]
**Subtotal (95% CI)**			**557**			**616**	**3.5%**	**0.34 [0.12, 0.55]**
Heterogeneity: $Tau^2 = 0.02$; $Chi^2 = 3.40$, df = 1 (P = 0.07); $I^2 = 71\%$								
Test for overall effect: Z = 3.10 (P = 0.002)								
**Total (95% CI)**			**10663**			**10002**	**100.0%**	**0.26 [0.16, 0.35]**
Heterogeneity: $Tau^2 = 0.13$; $Chi^2 = 662.50$, df = 70 (P < 0.00001); $I^2 = 89\%$								
Test for overall effect: Z = 5.36 (P < 0.00001)								
Test for subgroup differences: $Chi^2 = 12.06$, df = 3 (P = 0.007), $I^2 = 75.1\%$								

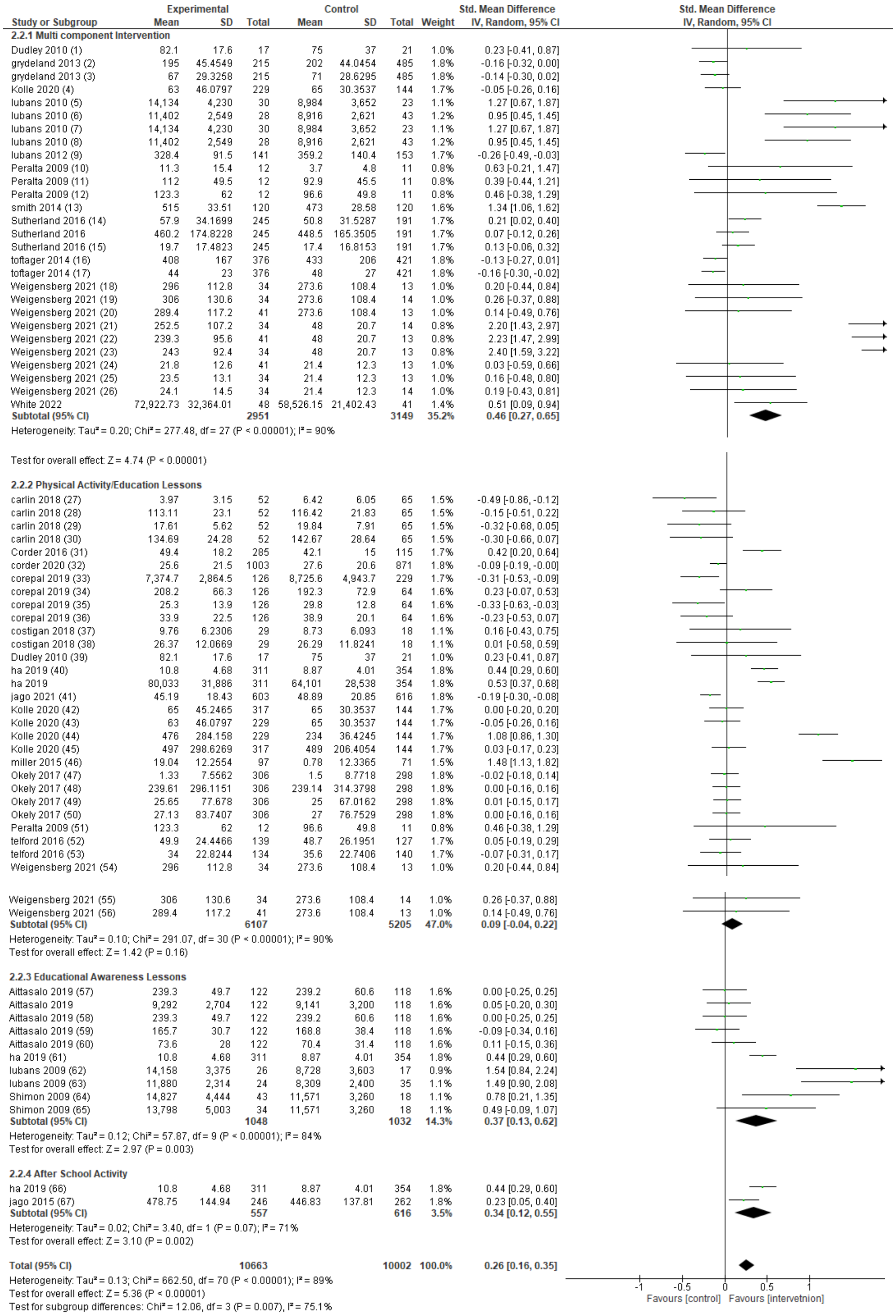

Figure 13: Forest plot - Physical activity Intervention type sub group analysis

Study or Subgroup	Experimental Mean	Experimental SD	Experimental Total	Control Mean	Control SD	Control Total	Weight	Std. Mean Difference IV, Random, 95% CI
**2.12.1 Cardiorespiratory fitness**								
Ardoy 2011 (1)	12	0.8	23	12.4	1	9	3.6%	-0.45 [-1.23, 0.33]
Ardoy 2011 (2)	12.4	1.2	23	12.4	1	9	3.6%	0.00 [-0.77, 0.77]
Costigan 2015 (3)	61.9	21.7692	16	60.6	14.9576	10	3.5%	0.06 [-0.73, 0.85]
Costigan 2015 (4)	64.5	21.3944	17	60.6	13.9202	9	3.4%	0.20 [-0.61, 1.01]
Eather 2015 (5)	71	23.9	49	46.3	19.3	34	5.8%	1.11 [0.64, 1.58]
Gallotta 2009 (6)	9.3	1.3	77	9.3	1.3	75	7.1%	0.00 [-0.32, 0.32]
Guijjaro-Romero 2022 (7)	288.2	20.1	34	232.1	28	10	3.0%	2.50 [1.61, 3.39]
Guijjaro-Romero 2022 (8)	355.6	19	39	232.1	28	9	1.5%	5.83 [4.41, 7.24]
Jago 2011 (9)	27.3	17.1	1871	27.3	17.5	1839	8.8%	0.00 [-0.06, 0.06]
Juric 2023 (10)	906.6	457.5	105	862.5	416.1	102	7.5%	0.10 [-0.17, 0.37]
Kim 2011 (11)	22.5	2.2	21	23.2	1.6	10	3.7%	-0.34 [-1.09, 0.42]
Peralta 2009 (12)	49.3	13.7	16	48.4	12.5	17	4.2%	0.07 [-0.62, 0.75]
Seger 2022 (13)	6.84	2.28	122	6.93	1.91	26	6.2%	-0.04 [-0.46, 0.38]
Singh 2007 (14)	7.65	1.93	322	7.44	1.83	239	8.3%	0.11 [-0.06, 0.28]
Singh 2007 (15)	9.33	2.03	278	9	2.23	214	8.2%	0.16 [-0.02, 0.33]
Suchert 2015 (16)	56.44	2.25	702	56.92	2.85	460	8.6%	-0.19 [-0.31, -0.07]
Tarp 2016 (17)	1,018	109	162	1,040	19	297	8.1%	-0.33 [-0.52, -0.14]
Torbeyns 2017 (18)	7	2.9	21	6.8	2.9	23	4.8%	0.07 [-0.52, 0.66]
**Subtotal (95% CI)**			**3898**			**3392**	**100.0%**	**0.20 [0.01, 0.39]**

Heterogeneity: Tau² = 0.11; Chi² = 145.36, df = 17 (P < 0.00001); I² = 88%
Test for overall effect: Z = 2.03 (P = 0.04)

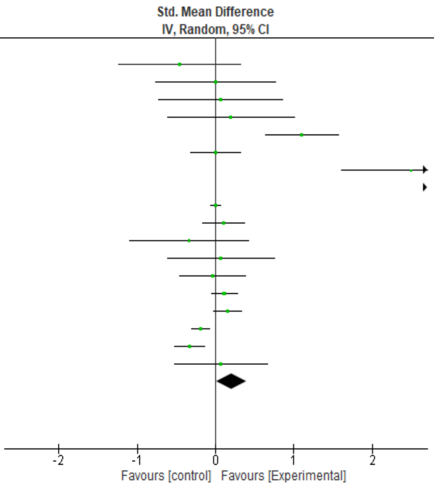

Figure 14: Forest Plot - Physical Fitness Subgroup Analysis - Cardiorespiratory fitness

Study or Subgroup	Experimental Mean	Experimental SD	Experimental Total	Control Mean	Control SD	Control Total	Weight	Std. Mean Difference IV, Random, 95% CI
**2.12.2 Aerobic Fitness**								
solberg 2021 (19)	925	108	332	940	92	241	50.1%	-0.15 [-0.31, 0.02]
solberg 2021 (20)	909	90	332	940	92	242	49.9%	-0.34 [-0.51, -0.17]
**Subtotal (95% CI)**			**664**			**483**	**100.0%**	**-0.24 [-0.43, -0.05]**

Heterogeneity: Tau² = 0.01; Chi² = 2.59, df = 1 (P = 0.11); I² = 61%
Test for overall effect: Z = 2.52 (P = 0.01)

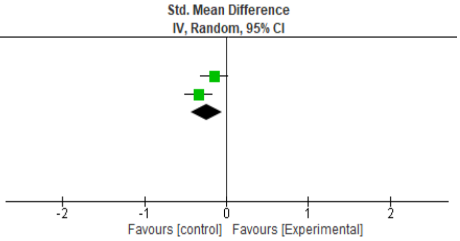

Figure 15: Forest Plot - Physical Fitness Subgroup Analysis - Aerobic Fitness

Study or Subgroup	Experimental Mean	Experimental SD	Experimental Total	Control Mean	Control SD	Control Total	Weight	Std. Mean Difference IV, Random, 95% CI
**2.13.8 0-6 Months**								
Ardoy 2011 (1)	42.6	4.6	25	40.3	5.7	9	2.6%	0.46 [-0.31, 1.23]
Ardoy 2011 (2)	12	0.8	23	12.4	1	9	2.6%	-0.45 [-1.23, 0.33]
Ardoy 2011 (3)	12.4	1.2	23	12.4	1	9	2.6%	0.00 [-0.77, 0.77]
Ardoy 2011 (4)	47.1	5.7	23	40.3	5.7	9	2.5%	1.16 [0.33, 1.99]
Borrestad 2012 (5)	49.7	5.2	24	50.6	5	22	3.2%	-0.17 [-0.75, 0.41]
Costigan 2015 (6)	61.9	21.7692	16	60.6	14.9576	10	2.6%	0.06 [-0.73, 0.85]
Costigan 2015 (7)	64.5	21.3944	17	60.6	13.9202	9	2.5%	0.20 [-0.61, 1.01]
Eather 2015 (8)	71	23.9	49	46.3	19.3	34	3.6%	1.11 [0.64, 1.58]
Gallotta 2009 (9)	9.3	1.3	77	9.3	1.3	75	4.1%	0.00 [-0.32, 0.32]
Guijjaro-Romero 2022 (10)	355.6	19	39	232.1	28	9	1.3%	5.83 [4.41, 7.24]
Guijjaro-Romero 2022 (11)	288.2	20.1	34	232.1	28	10	2.3%	2.50 [1.61, 3.39]
Guijjaro-Romero 2022 (12)	46	0.8	39	41.6	1.2	10	1.6%	4.86 [3.64, 6.08]
Guijjaro-Romero 2022 (13)	42.9	0.9	34	41.6	1.2	9	2.6%	1.32 [0.53, 2.11]
Juric 2023 (14)	906.6	457.5	105	862.5	416.1	102	4.3%	0.10 [-0.17, 0.37]
Kim 2011 (15)	32.8	7.5	21	32.6	4.5	10	2.7%	0.03 [-0.72, 0.78]
Kim 2011 (16)	22.5	2.2	21	23.2	1.6	10	2.7%	-0.34 [-1.09, 0.42]
Kim 2011 (17)	32.8	7.5	21	32.6	4.5	10	2.7%	0.03 [-0.72, 0.78]
Peralta 2009 (18)	49.3	13.7	16	48.4	12.5	17	2.9%	0.07 [-0.62, 0.75]
Suchert 2015 (19)	56.44	2.25	702	56.92	2.85	460	4.6%	-0.19 [-0.31, -0.07]
Tarp 2016 (20)	1,018	109	162	1,040	19	297	4.5%	-0.33 [-0.52, -0.14]
Torbeyns 2017 (21)	7	2.9	21	6.8	2.9	23	3.2%	0.07 [-0.52, 0.66]
**Subtotal (95% CI)**			**1492**			**1153**	**61.5%**	**0.58 [0.25, 0.91]**

Heterogeneity: Tau² = 0.46; Chi² = 221.39, df = 20 (P < 0.00001); I² = 91%
Test for overall effect: Z = 3.43 (P = 0.0006)

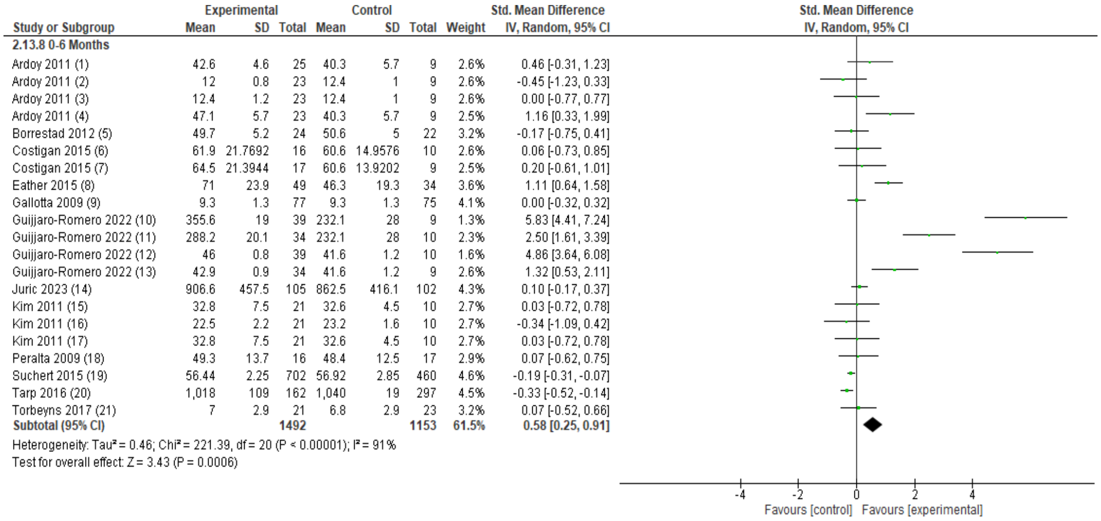

Figure 16: Forest Plot - Physical Fitness Subgroup Analysis - 0-6 months follow up

Study or Subgroup	Experimental Mean	Experimental SD	Experimental Total	Control Mean	Control SD	Control Total	Weight	Std. Mean Difference IV, Random, 95% CI
**2.13.9 7-12 months**								
Isensee 2018 (22)	48.59	0.54	709	48.14	0.7	414	4.6%	0.74 [0.62, 0.87]
Singh 2007 (23)	7.65	1.93	322	7.44	1.83	239	4.5%	0.11 [-0.06, 0.28]
Singh 2007 (24)	9.33	2.03	278	9	2.23	214	4.5%	0.16 [-0.02, 0.33]
solberg 2021 (25)	909	90	332	940	92	242	4.5%	-0.34 [-0.51, -0.17]
solberg 2021 (26)	925	108	332	940	92	241	4.5%	-0.15 [-0.31, 0.02]
**Subtotal (95% CI)**			**1973**			**1350**	**22.6%**	**0.11 [-0.30, 0.51]**
Heterogeneity: Tau² = 0.21; Chi² = 131.26, df = 4 (P < 0.00001); I² = 97%								
Test for overall effect: Z = 0.51 (P = 0.61)								

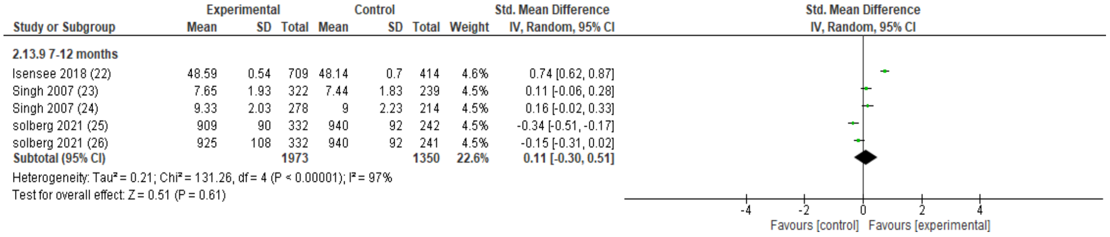

Figure 17: Forest Plot - Physical Fitness Subgroup Analysis - 7-12 months follow up

Study or Subgroup	Experimental Mean	Experimental SD	Experimental Total	Control Mean	Control SD	Control Total	Weight	Std. Mean Difference IV, Random, 95% CI
**2.13.10 2 Year Followup**								
Seger 2022 (27)	6.84	2.28	122	6.93	1.91	26	3.8%	-0.04 [-0.46, 0.38]
**Subtotal (95% CI)**			**122**			**26**	**3.8%**	**-0.04 [-0.46, 0.38]**
Heterogeneity: Not applicable								
Test for overall effect: Z = 0.19 (P = 0.85)								
**2.13.11 3 Year Followup**								
Jago 2011 (28)	27.3	17.1	1871	27.3	17.5	1839	4.7%	0.00 [-0.06, 0.06]
**Subtotal (95% CI)**			**1871**			**1839**	**4.7%**	**0.00 [-0.06, 0.06]**
Heterogeneity: Not applicable								
Test for overall effect: Z = 0.00 (P = 1.00)								
**2.13.13 4 Year Followup**								
UlrikeMariaMueller 2012 (29)	49.5	8.8	167	49.1	10	65	4.2%	0.04 [-0.24, 0.33]
UlrikeMariaMueller 2012 (30)	66	7.8	18	49.1	10	66	3.2%	1.75 [1.16, 2.33]
**Subtotal (95% CI)**			**185**			**131**	**7.4%**	**0.88 [-0.79, 2.54]**
Heterogeneity: Tau² = 1.40; Chi² = 26.10, df = 1 (P < 0.00001); I² = 96%								
Test for overall effect: Z = 1.03 (P = 0.30)								
**Total (95% CI)**			**5643**			**4499**	**100.0%**	**0.37 [0.18, 0.56]**
Heterogeneity: Tau² = 0.20; Chi² = 405.29, df = 29 (P < 0.00001); I² = 93%								
Test for overall effect: Z = 3.86 (P = 0.0001)								
Test for subgroup differences: Chi² = 12.56, df = 4 (P = 0.01), I² = 68.2%								

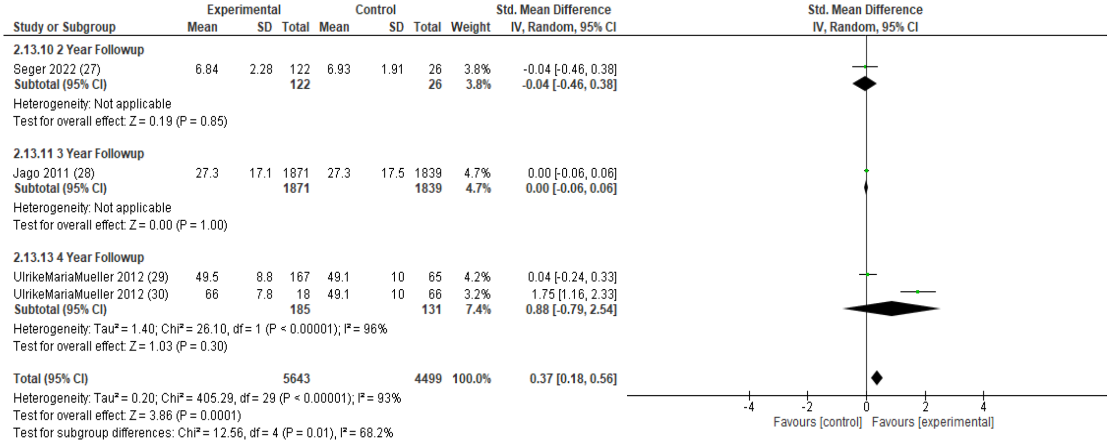

Figure 18: Forest Plot - Physical Fitness Subgroup Analysis - > 2 years follow up

Study or Subgroup	Experimental Mean	Experimental SD	Experimental Total	Control Mean	Control SD	Control Total	Weight	Std. Mean Difference IV, Random, 95% CI
**2.12.2 Multi component Intervention**								
Jago 2011 (1)	27.3	17.1	1871	27.3	17.5	1839	5.2%	0.00 [-0.06, 0.06]
Juric 2023 (2)	906.6	457.5	105	862.5	416.1	102	4.6%	0.10 [-0.17, 0.37]
Peralta 2009 (3)	49.3	13.7	16	48.4	12.5	17	3.0%	0.07 [-0.62, 0.75]
Singh 2007 (4)	7.65	1.93	322	7.44	1.83	239	5.0%	0.11 [-0.06, 0.28]
Singh 2007 (5)	9.33	2.03	278	9	2.23	214	4.9%	0.16 [-0.02, 0.33]
solberg 2021 (6)	925	108	332	940	92	241	5.0%	-0.15 [-0.31, 0.02]
solberg 2021 (7)	909	90	332	940	92	242	5.0%	-0.34 [-0.51, -0.17]
Suchert 2015 (8)	56.44	2.25	702	56.92	2.85	460	5.1%	-0.19 [-0.31, -0.07]
**Subtotal (95% CI)**			**3958**			**3354**	**37.8%**	**-0.05 [-0.17, 0.07]**
Heterogeneity: Tau² = 0.02; Chi² = 30.73, df = 7 (P < 0.0001); I² = 77%								
Test for overall effect: Z = 0.84 (P = 0.40)								
**2.12.3 Physical Activity/Education Lessons**								
Ardoy 2011 (9)	42.6	4.6	25	40.3	5.7	9	2.7%	0.46 [-0.31, 1.23]
Ardoy 2011 (10)	47.1	5.7	23	40.3	5.7	9	2.5%	1.16 [0.33, 1.99]
Ardoy 2011 (11)	12.4	1.2	23	12.4	1	9	2.7%	0.00 [-0.77, 0.77]
Ardoy 2011 (12)	12	0.8	23	12.4	1	9	2.6%	-0.45 [-1.23, 0.33]
Borrestad 2012 (13)	49.7	5.2	24	50.6	5	22	3.4%	-0.17 [-0.75, 0.41]
Costigan 2015 (14)	61.9	21.7692	16	60.6	14.9576	10	2.6%	0.06 [-0.73, 0.85]
Costigan 2015 (15)	64.5	21.3944	17	60.6	13.9202	9	2.5%	0.20 [-0.61, 1.01]
Eather 2015 (16)	71	23.9	49	46.3	19.3	34	3.8%	1.11 [0.64, 1.58]
Gallotta 2009 (17)	9.3	1.3	77	9.3	1.3	75	4.5%	0.00 [-0.32, 0.32]
Guijjaro-Romero 2022 (18)	355.6	19	39	232.1	28	9	1.2%	5.83 [4.41, 7.24]
Guijjaro-Romero 2022 (19)	288.2	20.1	34	232.1	28	10	2.3%	2.50 [1.61, 3.39]
Kim 2011 (20)	22.5	2.2	21	23.2	1.6	10	2.7%	-0.34 [-1.09, 0.42]
Seger 2022 (21)	6.84	2.28	122	6.93	1.91	26	4.0%	-0.04 [-0.46, 0.38]
Tarp 2016 (22)	1,018	109	162	1,040	19	297	4.9%	-0.33 [-0.52, -0.14]
Torbeyns 2017 (23)	7	2.9	21	6.8	2.9	23	3.3%	0.07 [-0.52, 0.66]
UlrikeMariaMueller 2012 (24)	49.5	8.8	167	49.1	10	65	4.6%	0.04 [-0.24, 0.33]
UlrikeMariaMueller 2012 (25)	66	7.8	18	49.1	10	66	3.4%	1.75 [1.16, 2.33]
**Subtotal (95% CI)**			**861**			**692**	**53.8%**	**0.56 [0.16, 0.96]**
Heterogeneity: Tau² = 0.59; Chi² = 169.84, df = 16 (P < 0.00001); I² = 91%								
Test for overall effect: Z = 2.73 (P = 0.006)								
**2.12.4 Educational Awareness Lessons**								
Isensee 2018 (26)	48.59	0.54	709	48.14	0.7	414	5.1%	0.74 [0.62, 0.87]
**Subtotal (95% CI)**			**709**			**414**	**5.1%**	**0.74 [0.62, 0.87]**
Heterogeneity: Not applicable								
Test for overall effect: Z = 11.67 (P < 0.00001)								
**2.12.5 After School Activity**								
Borrestad 2012 (27)	49.7	5.2	24	50.6	5	22	3.4%	-0.17 [-0.75, 0.41]
**Subtotal (95% CI)**			**24**			**22**	**3.4%**	**-0.17 [-0.75, 0.41]**
Heterogeneity: Not applicable								
Test for overall effect: Z = 0.59 (P = 0.56)								
**Total (95% CI)**			**5552**			**4482**	**100.0%**	**0.26 [0.07, 0.44]**
Heterogeneity: Tau² = 0.16; Chi² = 336.76, df = 26 (P < 0.00001); I² = 92%								
Test for overall effect: Z = 2.77 (P = 0.006)								
Test for subgroup differences: Chi² = 86.31, df = 3 (P < 0.00001), I² = 96.5%								

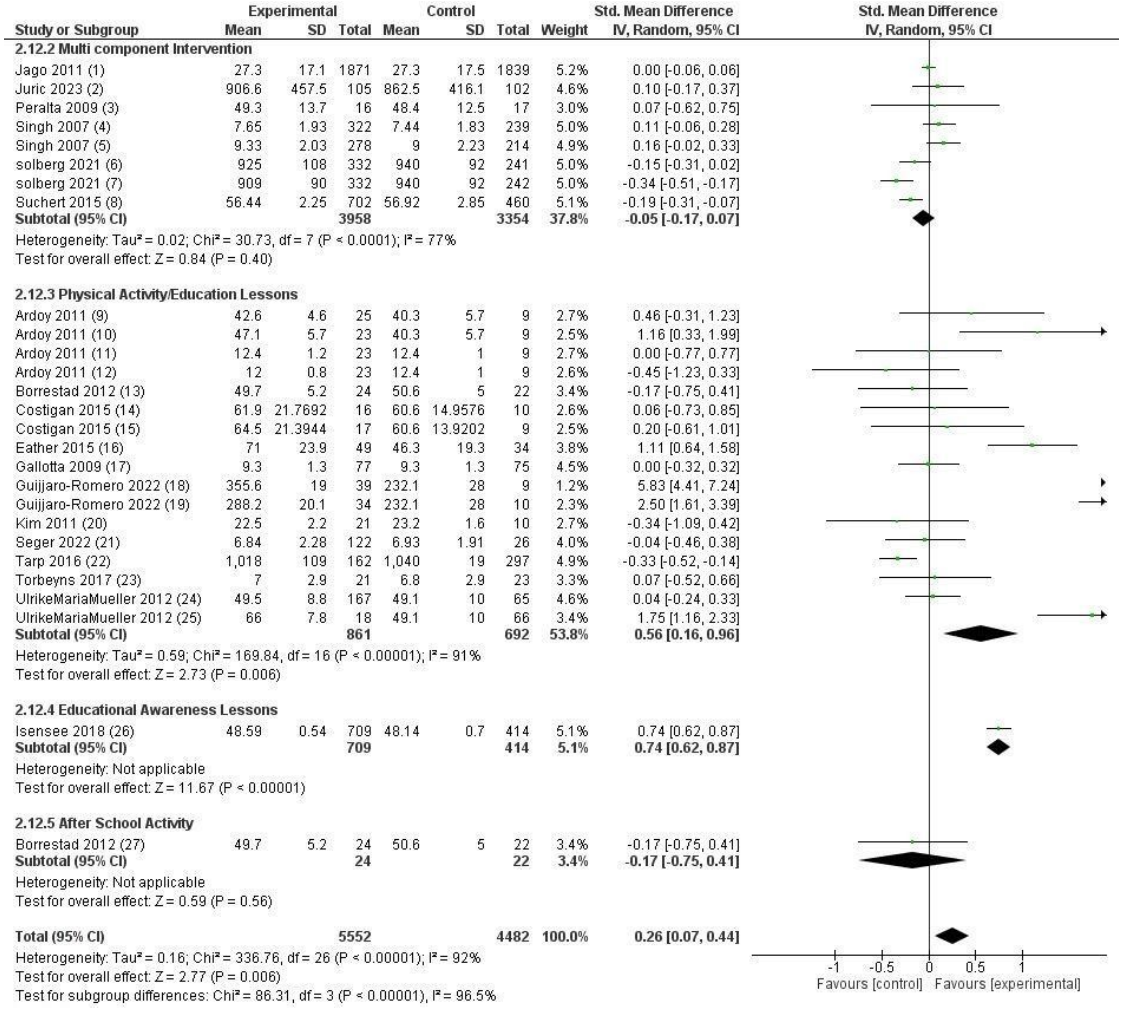

Figure 19: Forest plot - physical fitness intervention type subgroup analysis

Study or Subgroup	Experimental Mean	Experimental SD	Experimental Total	Control Mean	Control SD	Control Total	Weight	Std. Mean Difference IV, Random, 95% CI
Bakhoya 2016 (1)	42.3	2.91	109	42.5	3.01	55	3.8%	-0.07 [-0.39, 0.26]
Bakhoya 2016 (2)	42.6	4.1	109	42.1	3.01	54	3.8%	0.13 [-0.19, 0.46]
corepal 2019	460.6	89	126	464.4	100.1	64	4.4%	-0.04 [-0.34, 0.26]
hankonen 2017	0.66	0.1	12	0.63	0.14	3	0.3%	0.26 [-1.01, 1.53]
jago 2015 (3)	515	80.22	246	533.01	80.54	262	11.2%	-0.22 [-0.40, -0.05]
jago 2015 (4)	463.66	105.92	124	475.14	95.22	145	6.6%	-0.11 [-0.35, 0.13]
jago 2021 (5)	595.88	100.28	603	589.95	96.7	616	20.8%	0.06 [-0.05, 0.17]
jago 2021 (6)	528.45	122.56	347	535.79	115.78	386	14.8%	-0.06 [-0.21, 0.08]
Okely 2017	497.43	380.9226	306	508.2	381.5715	298	12.9%	-0.03 [-0.19, 0.13]
sebire 2018 (7)	510.78	85.29	162	511.65	103.21	81	5.5%	-0.01 [-0.28, 0.26]
sebire 2018 (8)	471.95	97.2	74	473.06	93.74	35	2.6%	-0.01 [-0.41, 0.39]
telford 2016 (9)	401.3	60.27	134	403.3	67.6	140	6.8%	-0.03 [-0.27, 0.21]
telford 2016 (10)	382.8	58.4	139	402.8	67.2	127	6.5%	-0.32 [-0.56, -0.08]
**Total (95% CI)**			**2491**			**2266**	**100.0%**	**-0.06 [-0.12, 0.01]**
Heterogeneity: Tau² = 0.00; Chi² = 14.16, df = 12 (P = 0.29); I² = 15%								
Test for overall effect: Z = 1.64 (P = 0.10)								

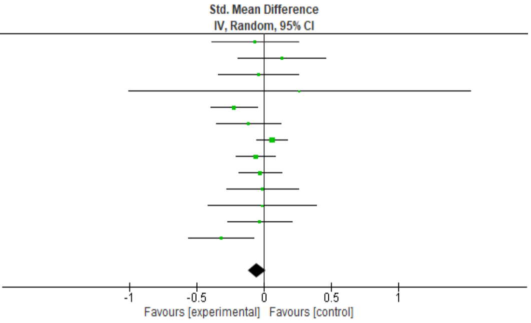

Figure 20: Forest Plot - Sedentary time

Study or Subgroup	Experimental Mean	Experimental SD	Experimental Total	Control Mean	Control SD	Control Total	Weight	Std. Mean Difference IV, Random, 95% CI
**2.4.1 0-6 months**								
hankonen 2017	0.66	0.1	12	0.63	0.14	3	0.3%	0.26 [-1.01, 1.53]
sebire 2018 (1)	471.95	97.2	74	473.06	93.74	35	2.6%	-0.01 [-0.41, 0.39]
sebire 2018 (2)	510.78	85.29	162	511.65	103.21	81	5.5%	-0.01 [-0.28, 0.26]
**Subtotal (95% CI)**			**248**			**119**	**8.3%**	**-0.00 [-0.22, 0.22]**
Heterogeneity: Tau² = 0.00; Chi² = 0.17, df = 2 (P = 0.92); I² = 0%								
Test for overall effect: Z = 0.02 (P = 0.99)								

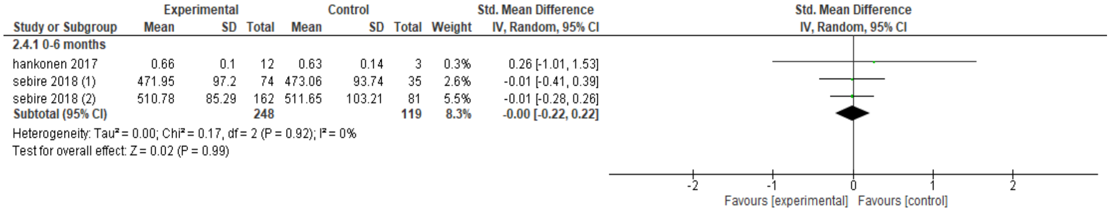

Figure 21: Forest Plot Sedentary Time Subgroup Analysis 0-6 months follow up

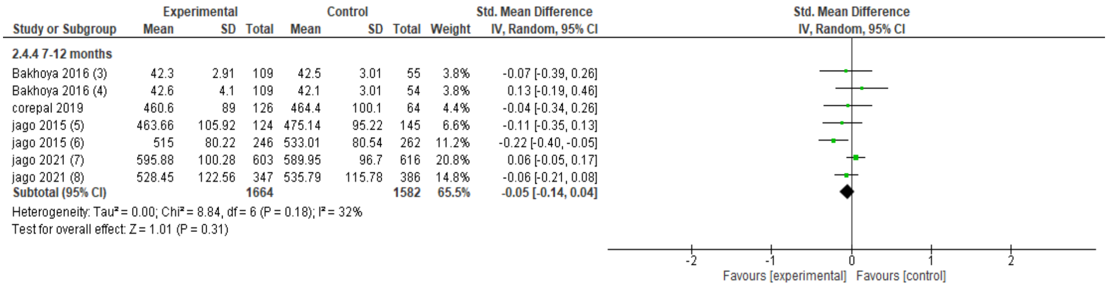

Figure 22: Forest Plot Sedentary Time Subgroup Analysis 7-12 months follow up

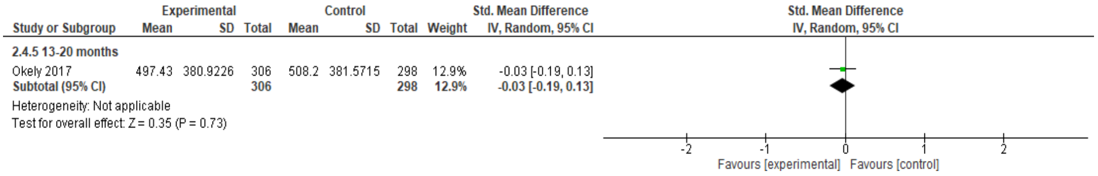

Figure 23: Forest Plot Sedentary Time Subgroup Analysis 13-20 months follow up

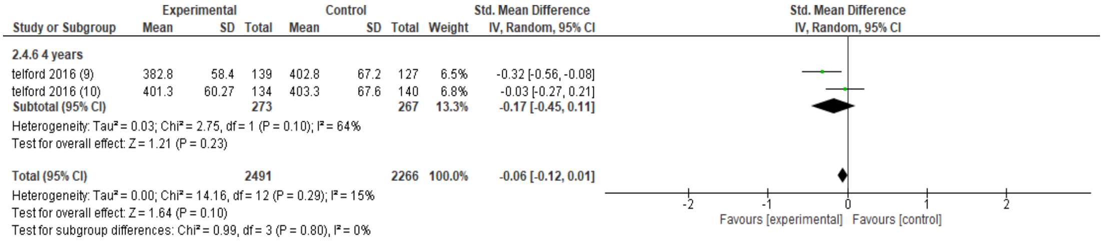

Figure 24: Forest Plot Sedentary Time Subgroup Analysis 4 years follow up

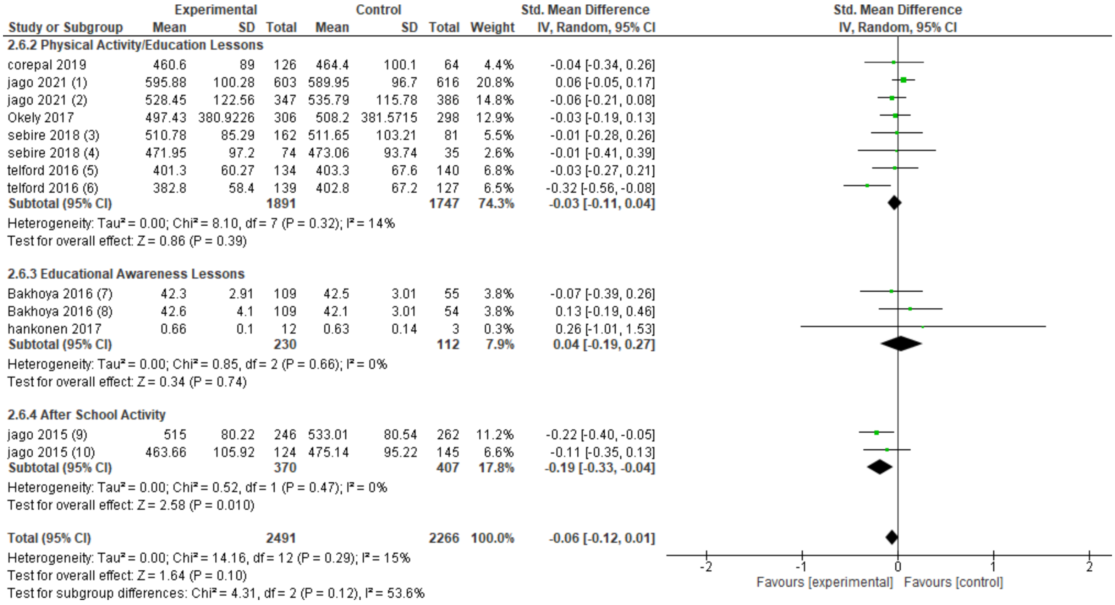

Figure 25: Forest plot – sedentary time subgroup analysis

Study or Subgroup	Experimental Mean	SD	Total	Control Mean	SD	Total	Weight	Std. Mean Difference IV, Random, 95% CI
Borrestad 2012 (1)	18.8	0.8	24	18.6	0.8	22	2.9%	0.25 [-0.34, 0.83]
Costigan 2015 (2)	22.07	2.8999	21	22.63	1.9648	11	2.6%	-0.21 [-0.94, 0.52]
Costigan 2015 (3)	21.8	2.9462	19	22.63	1.8452	10	2.5%	-0.31 [-1.08, 0.46]
DL dewar 2013 (4)	28.36	4.77	113	24.11	5.07	121	3.3%	0.86 [0.59, 1.13]
Eather 2015	21.5	3	49	21.7	1.3	34	3.1%	-0.08 [-0.52, 0.36]
Haerens 2006 (5)	20.52	3.68	590	19.67	2.89	119	3.4%	0.24 [0.04, 0.44]
Haerens 2006 (6)	20.86	3.51	611	19.67	2.89	120	3.4%	0.35 [0.15, 0.54]
Haerens 2006 (7)	21.34	3.83	381	20.78	3.66	176	3.4%	0.15 [-0.03, 0.33]
Haerens 2006 (8)	21.66	3.68	118	20.78	3.66	176	3.3%	0.24 [0.01, 0.47]
Hollis 2016 (9)	21.64	4.06	547	21.9	4.33	409	3.4%	-0.06 [-0.19, 0.07]
Juric 2023 (10)	20.09	9.52	105	20.58	3.77	102	3.3%	-0.07 [-0.34, 0.21]
Kennedy 2018	22.35	5.5153	279	23.13	4.9348	188	3.4%	-0.15 [-0.33, 0.04]
Kurnaz 2024 (11)	39.69	7.71	25	42.76	11.35	25	2.9%	-0.31 [-0.87, 0.25]
luban 2012	23.3	4.71	141	23.37	4.68	153	3.3%	-0.01 [-0.24, 0.21]
Luban 2012 (12)	21.28	4.04	50	22.43	4.87	50	3.2%	-0.26 [-0.65, 0.14]
Parrish 2018 (13)	20.3	4.7	42	20.7	3.9	41	3.1%	-0.09 [-0.52, 0.34]
Peralta 2009 (14)	23.1	4.2	16	21	4.1	17	2.7%	0.49 [-0.20, 1.19]
Robbins 2006 (15)	22.55	4.26	4	20.64	4.5	9	1.8%	0.40 [-0.79, 1.59]
Rostami moez 2017 (16)	19.14	4.06	179	20.53	3.93	165	3.4%	-0.35 [-0.56, -0.13]
Shamah Levy 2012 (17)	45.3	108.1585	249	43.5	113.7667	249	3.4%	0.02 [-0.16, 0.19]
Shamah Levy 2012 (18)	54.3	124.182	249	60.8	123.6308	250	3.4%	-0.05 [-0.23, 0.12]
Singh 2007 (19)	18.61	2.76	278	19.43	2.92	214	3.4%	-0.29 [-0.47, -0.11]
Singh 2007 (20)	19.46	3.05	322	20	3.47	239	3.4%	-0.17 [-0.33, 0.00]
Smith 2014 (21)	21.3	0.64	181	21.2	0.57	180	3.4%	0.16 [-0.04, 0.37]
solberg 2021 (22)	20.5	3.2	245	20.1	3.1	241	3.4%	0.13 [-0.05, 0.30]
solberg 2021 (23)	20.8	3	246	3.1	3.1	242	3.1%	5.79 [5.39, 6.20]
Tarp 2016 (24)	20.1	2.9	191	19.7	3.1	417	3.4%	0.13 [-0.04, 0.30]
Torbeyns 2017 (25)	19.9	3.2	21	20.5	3.5	23	2.8%	-0.18 [-0.77, 0.42]
Weeks 2008 (26)	20.6	3.3	22	21.9	5.6	15	2.7%	-0.29 [-0.95, 0.37]
Weeks 2008 (27)	20.3	3.5	21	20	2	23	2.8%	0.10 [-0.49, 0.70]
Weeks 2012 (28)	20.5	3.3	52	20.4	3.7	47	3.2%	0.03 [-0.37, 0.42]
Young 2006 (29)	25.3	7.1	109	25.6	6.5	97	3.3%	-0.04 [-0.32, 0.23]
**Total (95% CI)**			**5500**			**4185**	**100.0%**	**0.20 [-0.04, 0.44]**

Heterogeneity: $Tau^2 = 0.43$; $Chi^2 = 874.31$, $df = 31$ ($P < 0.00001$); $I^2 = 96\%$
Test for overall effect: $Z = 1.65$ ($P = 0.10$)

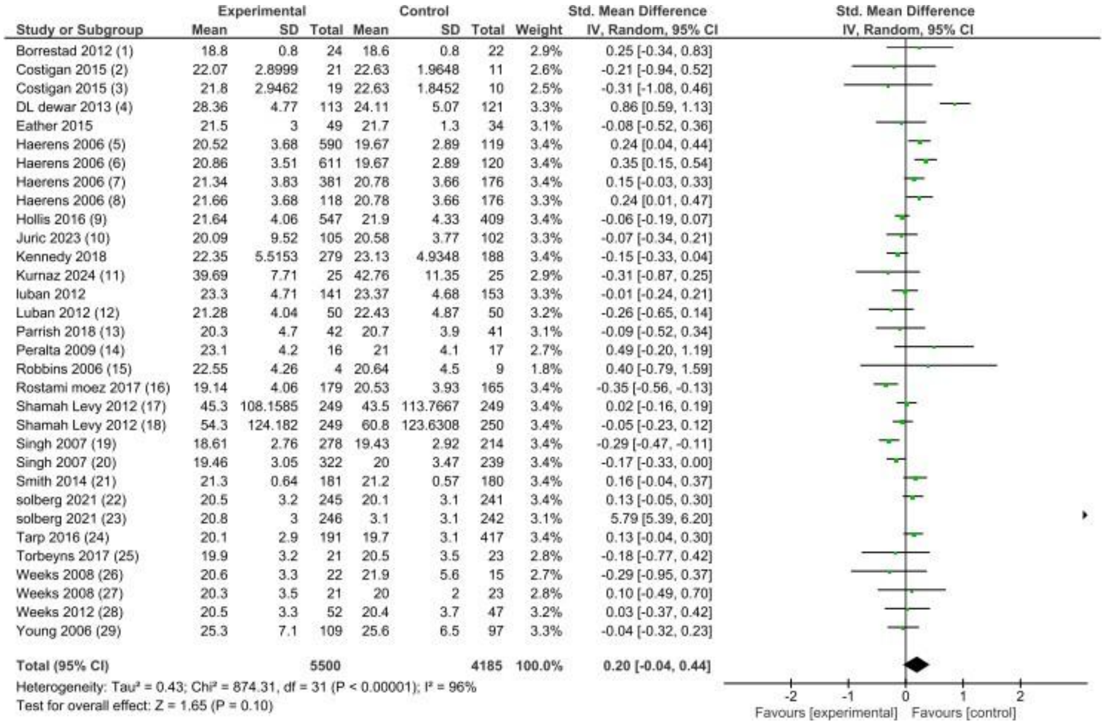

Figure 26: Forest plot BMI

Intention-to-treat	Unique ID	Study ID	Experimental	Comparator	Outcome	Weight	D1	D2	D3	D4	D5	Overall
	14	Borrestad 2012	Cycling to school interver	standard curriculum	BMI	1	+	+	+	+	+	+
	21	Costigan 2015	HIIT sessions	none	BMI	1	+	+	+	+	+	+
	26	DL Dewar 2013	NEAT girls intervention	NONE	BMI	1	!	!	+	+	!	!
	29	Eather 2015	CrossFit teens fitness pro	Standard PA	BMI	1	+	!	+	+	+	+
	39	Haerens 2006	PA based intervention	none	BMI	1	!	+	+	+	+	+
	42	Hollis 2016	PA4E1 intervention	none	BMI	1	+	+	+	+	+	+
	51	Juric 2023	HIIT sessions	regualar PA	BMI	1	+	+	+	+	+	+
	56	kennedy 2018	Resistence training for te	standard PA	BMI	1	+	+	+	+	+	+
	60	kurnaz 2024	Play based after school pr	none	BMI	1	+	+	+	+	+	+
	73	lubans 2012	multicomponent school-b	regular school curriculum	BMI	1	+	+	!	+	+	+
	83	parrish 2018	standing desks	none	BMI	1	+	+	+	+	+	+
	85	Peralta 2009	FILA program	Standard PA	BMI	1	+	+	+	+	+	+
	92	Robbins 2006	Girls on the move interve	standard PA	BMI	1	+	+	+	+	+	+
	94	Rosstami-Moez 201	theory based educational	standard curriculum	BMI	1	!	+	+	+	!	!
	98	Shamah Levy 2012	Nutrition on the Go inter	Standard PA	BMI	1	!	+	+	+	!	!
	101	Singh 2007	Doit program	Standard PA	BMI	1	!	+	+	+	+	!
	104	Smith 2014	ATLAS intervention	regualr school curriculum	BMI	1	+	+	+	+	+	+
	107	Solberg 2021	PAL & DWBH Interventio	No intervention	BMI	1	+	!	!	+	!	!
	112	tarp 2016	lcomotion learning	no intervention	BMI	1	+	+	+	+	+	+
	117	Torbeyns 2017	bike desks	none	BMI	1	!	+	+	+	!	!
	124	weeks 2008	school based exercise int	standard PA	BMI	1	!	!	+	+	+	!
	125	Weeks 2012	10 min jumping exercisex	usual warm ups	BMI	1	!	+	+	+	+	!
	131	Young 2006	Life skill based PA	standard PA	BMI	1	!	!	+	+	!	!

Figure 27: ROB BMI

Study or Subgroup	Experimental Mean	Experimental SD	Experimental Total	Control Mean	Control SD	Control Total	Weight	Std. Mean Difference IV, Random, 95% CI
**2.10.1 0-6 Months**								
Borrestad 2012 (1)	18.8	0.8	24	18.6	0.8	22	3.0%	0.25 [-0.34, 0.83]
Costigan 2015 (2)	21.8	2.9462	19	22.63	1.8452	10	2.6%	-0.31 [-1.08, 0.46]
Costigan 2015 (3)	22.07	2.8999	21	22.63	1.9648	11	2.7%	-0.21 [-0.94, 0.52]
Eather 2015	21.5	3	49	21.7	1.3	34	3.2%	-0.08 [-0.52, 0.36]
Juric 2023 (4)	20.09	9.52	105	20.58	3.77	102	3.4%	-0.07 [-0.34, 0.21]
Kurnaz 2024 (5)	39.69	7.71	25	42.76	11.35	25	3.0%	-0.31 [-0.87, 0.25]
Luban 2012 (6)	21.28	4.04	50	22.43	4.87	50	3.3%	-0.26 [-0.65, 0.14]
Parrish 2018 (7)	20.3	4.7	42	20.7	3.9	41	3.2%	-0.09 [-0.52, 0.34]
Peralta 2009 (8)	23.1	4.2	16	21	4.1	17	2.8%	0.49 [-0.20, 1.19]
Robbins 2006 (9)	22.55	4.26	4	20.64	4.5	9	1.9%	0.40 [-0.79, 1.59]
Shamah Levy 2012 (10)	54.3	124.182	249	60.8	123.6308	250	3.5%	-0.05 [-0.23, 0.12]
Shamah Levy 2012 (11)	45.3	108.1585	249	43.5	113.7667	249	3.5%	0.02 [-0.16, 0.19]
Tarp 2016 (12)	20.1	2.9	191	19.7	3.1	417	3.5%	0.13 [-0.04, 0.30]
Torbeyns 2017 (13)	19.9	3.2	21	20.5	3.5	23	2.9%	-0.18 [-0.77, 0.42]
**Subtotal (95% CI)**			**1065**			**1260**	**42.5%**	**-0.00 [-0.09, 0.08]**

Heterogeneity: Tau² = 0.00; Chi² = 10.29, df = 13 (P = 0.67); I² = 0%
Test for overall effect: Z = 0.09 (P = 0.93)

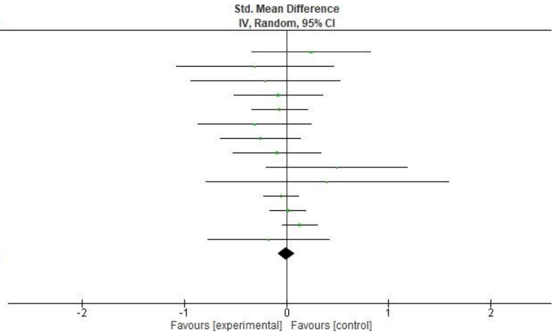

Figure 28: Forest plot BMI sub group analysis- 0-6 months follow up

Study or Subgroup	Experimental Mean	Experimental SD	Experimental Total	Control Mean	Control SD	Control Total	Weight	Std. Mean Difference IV, Random, 95% CI
**2.10.3 7-12 Months**								
Kennedy 2018	22.35	5.5153	279	23.13	4.9348	188	3.5%	-0.15 [-0.33, 0.04]
luban 2012	23.3	4.71	141	23.37	4.68	153	3.5%	-0.01 [-0.24, 0.21]
Singh 2007 (14)	18.61	2.76	278	19.43	2.92	214	3.5%	-0.29 [-0.47, -0.11]
Singh 2007 (15)	19.46	3.05	322	20	3.47	239	3.5%	-0.17 [-0.33, 0.00]
Smith 2014 (16)	21.3	0.64	181	21.2	0.57	180	3.5%	0.16 [-0.04, 0.37]
solberg 2021 (17)	20.8	3	246	3.1	3.1	242	3.2%	5.79 [5.39, 6.20]
solberg 2021 (18)	20.5	3.2	245	20.1	3.1	241	3.5%	0.13 [-0.05, 0.30]
Weeks 2008 (19)	20.3	3.5	21	20	2	23	2.9%	0.10 [-0.49, 0.70]
Weeks 2008 (20)	20.6	3.3	22	21.9	5.6	15	2.8%	-0.29 [-0.95, 0.37]
Weeks 2012 (21)	20.5	3.3	52	20.4	3.7	47	3.3%	0.03 [-0.37, 0.42]
Young 2006 (22)	25.3	7.1	109	25.6	6.5	97	3.4%	-0.04 [-0.32, 0.23]
**Subtotal (95% CI)**			**1896**			**1639**	**36.6%**	**0.47 [-0.17, 1.12]**

Heterogeneity: Tau² = 1.17; Chi² = 794.78, df = 10 (P < 0.00001); I² = 99%
Test for overall effect: Z = 1.44 (P = 0.15)

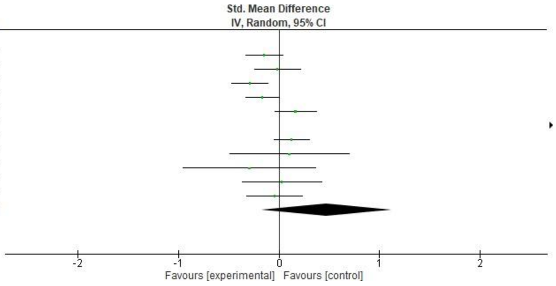

Figure 29: Forest plot BMI sub group analysis- 7-12 months follow up

Study or Subgroup	Experimental Mean	Experimental SD	Experimental Total	Control Mean	Control SD	Control Total	Weight	Std. Mean Difference IV, Random, 95% CI
**2.10.4 21-24 months**								
DL dewar 2013 (23)	28.36	4.77	113	24.11	5.07	121	3.4%	0.86 [0.59, 1.13]
Haerens 2006 (24)	20.52	3.68	590	19.67	2.89	119	3.5%	0.24 [0.04, 0.44]
Haerens 2006 (25)	20.86	3.51	611	19.67	2.89	120	3.5%	0.35 [0.15, 0.54]
Haerens 2006 (26)	21.34	3.83	381	20.78	3.66	176	3.5%	0.15 [-0.03, 0.33]
Haerens 2006 (27)	21.66	3.68	118	20.78	3.66	176	3.5%	0.24 [0.01, 0.47]
Hollis 2016 (28)	21.64	4.06	547	21.9	4.33	409	3.5%	-0.06 [-0.19, 0.07]
**Subtotal (95% CI)**			**2360**			**1121**	**20.9%**	**0.28 [0.05, 0.51]**

Heterogeneity: Tau² = 0.07; Chi² = 41.90, df = 5 (P < 0.00001); I² = 88%
Test for overall effect: Z = 2.43 (P = 0.01)

**Total (95% CI)**			**5321**			**4020**	**100.0%**	**0.22 [-0.03, 0.46]**

Heterogeneity: Tau² = 0.44; Chi² = 857.77, df = 30 (P < 0.00001); I² = 97%
Test for overall effect: Z = 1.76 (P = 0.08)
Test for subgroup differences: Chi² = 7.15, df = 2 (P = 0.03), I² = 72.0%

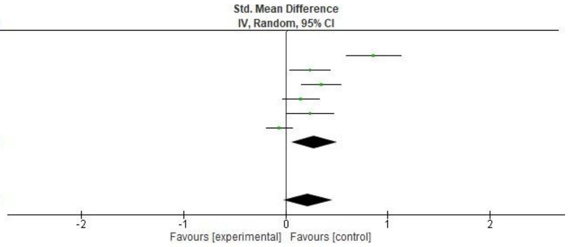

Figure 30: Forest plot BMI sub group analysis- 21- 24 months follow up

Study or Subgroup	Experimental Mean	Experimental SD	Experimental Total	Control Mean	Control SD	Control Total	Weight	Std. Mean Difference IV, Random, 95% CI
**2.11.9 High Income Countries**								
Borrestad 2012 (1)	18.8	0.8	24	18.6	0.8	22	2.8%	0.25 [-0.34, 0.83]
Costigan 2015 (2)	22.07	2.8999	21	22.63	1.9648	11	2.5%	-0.21 [-0.94, 0.52]
Costigan 2015 (3)	21.8	2.9462	19	22.63	1.8452	10	2.4%	-0.31 [-1.08, 0.46]
DL dewar 2013 (4)	28.36	4.77	113	24.11	5.07	121	3.2%	0.86 [0.59, 1.13]
Eather 2015	21.5	3	49	21.7	1.3	34	3.0%	-0.08 [-0.52, 0.36]
Haerens 2006 (5)	20.52	3.68	590	19.67	2.89	119	3.3%	0.24 [0.04, 0.44]
Haerens 2006 (6)	20.86	3.51	611	19.67	2.89	120	3.3%	0.35 [0.15, 0.54]
Haerens 2006 (7)	21.34	3.83	381	20.78	3.66	176	3.3%	0.15 [-0.03, 0.33]
Haerens 2006 (8)	21.66	3.68	118	20.78	3.66	176	3.2%	0.24 [0.01, 0.47]
Hollis 2016 (9)	21.64	4.06	547	21.9	4.33	409	3.3%	-0.06 [-0.19, 0.07]
Juric 2023 (10)	20.09	9.52	105	20.58	3.77	102	3.2%	-0.07 [-0.34, 0.21]
Kennedy 2018	22.35	5.5153	279	23.13	4.9348	188	3.3%	-0.15 [-0.33, 0.04]
luban 2012	23.3	4.71	141	23.37	4.68	153	3.2%	-0.01 [-0.24, 0.21]
Luban 2012 (11)	21.28	4.04	50	22.43	4.87	50	3.1%	-0.26 [-0.65, 0.14]
Luban 2016 (12)	22.3	0.7	121	22.3	0.6	143	3.2%	0.00 [-0.24, 0.24]
Parrish 2018 (13)	20.3	4.7	42	20.7	3.9	41	3.0%	-0.09 [-0.52, 0.34]
Peralta 2009 (14)	23.1	4.2	16	21	4.1	17	2.6%	0.49 [-0.20, 1.19]
Robbins 2006 (15)	22.55	4.26	4	20.64	4.5	9	1.8%	0.40 [-0.79, 1.59]
Singh 2007 (16)	18.61	2.76	278	19.43	2.92	214	3.3%	-0.29 [-0.47, -0.11]
Singh 2007 (17)	19.46	3.05	322	20	3.47	239	3.3%	-0.17 [-0.33, 0.00]
Smith 2014 (18)	21.3	0.64	181	21.2	0.57	180	3.3%	0.16 [-0.04, 0.37]
solberg 2021 (19)	20.5	3.2	245	20.1	3.1	241	3.3%	0.13 [-0.05, 0.30]
solberg 2021 (20)	20.8	3	246	3.1	3.1	242	3.0%	5.79 [5.39, 6.20]
Tarp 2016 (21)	20.1	2.9	191	19.7	3.1	417	3.3%	0.13 [-0.04, 0.30]
Torbeyns 2017 (22)	19.9	3.2	21	20.5	3.5	23	2.7%	-0.18 [-0.77, 0.42]
Weeks 2008 (23)	20.6	3.3	22	21.9	5.6	15	2.6%	-0.29 [-0.95, 0.37]
Weeks 2008 (24)	20.3	3.5	21	20	2	23	2.7%	0.10 [-0.49, 0.70]
Weeks 2012 (25)	20.5	3.3	52	20.4	3.7	47	3.0%	0.03 [-0.37, 0.42]
Young 2006 (26)	25.3	7.1	109	25.6	6.5	97	3.2%	-0.04 [-0.32, 0.23]
**Subtotal (95% CI)**			**4919**			**3639**	**87.4%**	**0.25 [-0.02, 0.51]**

Heterogeneity: Tau² = 0.49; Chi² = 851.86, df = 28 (P < 0.00001); I² = 97%
Test for overall effect: Z = 1.82 (P = 0.07)

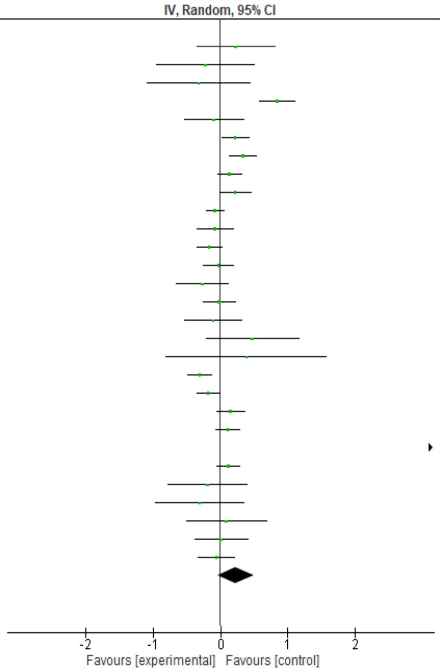

Figure 31: Forest Plot - BMI Sub group analysis high income countries

Study or Subgroup	Experimental Mean	Experimental SD	Experimental Total	Control Mean	Control SD	Control Total	Weight	Std. Mean Difference IV, Random, 95% CI
**2.11.10 Upper Middle Income Countries**								
Kurnaz 2024 (27)	39.69	7.71	25	42.76	11.35	25	2.8%	-0.31 [-0.87, 0.25]
Rostami moez 2017 (28)	19.14	4.06	179	20.53	3.93	165	3.3%	-0.35 [-0.56, -0.13]
Shamah Lewy 2012 (29)	45.3	108.1585	249	43.5	113.7667	249	3.3%	0.02 [-0.16, 0.19]
Shamah Lewy 2012 (30)	54.3	124.182	249	60.8	123.6308	250	3.3%	-0.05 [-0.23, 0.12]
**Subtotal (95% CI)**			**702**			**689**	**12.6%**	**-0.14 [-0.32, 0.05]**
**Total (95% CI)**			**5621**			**4328**	**100.0%**	**0.19 [-0.04, 0.42]**

Heterogeneity: Tau² = 0.02; Chi² = 7.64, df = 3 (P = 0.05); I² = 61%
Test for overall effect: Z = 1.44 (P = 0.15)

Heterogeneity: Tau² = 0.41; Chi² = 874.78, df = 32 (P < 0.00001); I² = 96%
Test for overall effect: Z = 1.65 (P = 0.10)
Test for subgroup differences: Chi² = 5.37, df = 1 (P = 0.02), I² = 81.4%

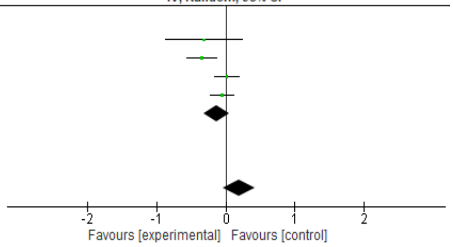

Figure 32: Forest plot - BMI Subgroup analysis Upper Middle-Income Countries

Study or Subgroup	Experimental Mean	Experimental SD	Experimental Total	Control Mean	Control SD	Control Total	Weight	Std. Mean Difference IV, Random, 95% CI
**2.5.1 Multi component Intervention**								
DL dewar 2013 (1)	28.36	4.77	113	24.11	5.07	121	3.3%	0.86 [0.59, 1.13]
Hollis 2016 (2)	21.64	4.06	547	21.9	4.33	409	3.4%	-0.06 [-0.19, 0.07]
Kennedy 2018	22.35	5.5153	279	23.13	4.9348	188	3.4%	-0.15 [-0.33, 0.04]
luban 2012	23.3	4.71	141	23.37	4.68	153	3.4%	-0.01 [-0.24, 0.21]
Luban 2012 (3)	21.28	4.04	50	22.43	4.87	50	3.2%	-0.26 [-0.65, 0.14]
Peralta 2009 (4)	23.1	4.2	16	21	4.1	17	2.7%	0.49 [-0.20, 1.19]
Singh 2007 (5)	18.61	2.76	278	19.43	2.92	214	3.4%	-0.29 [-0.47, -0.11]
Singh 2007 (6)	19.46	3.05	322	20	3.47	239	3.4%	-0.17 [-0.33, 0.00]
solberg 2021 (7)	20.5	3.2	245	20.1	3.1	241	3.4%	0.13 [-0.05, 0.30]
solberg 2021 (8)	20.8	3	246	3.1	3.1	242	3.2%	5.79 [5.39, 6.20]
**Subtotal (95% CI)**			**2237**			**1874**	**32.7%**	**0.62 [-0.03, 1.27]**
Heterogeneity: Tau² = 1.07; Chi² = 833.13, df = 9 (P < 0.00001); I² = 99%								
Test for overall effect: Z = 1.88 (P = 0.06)								
**2.5.2 Physical Activity/Education Lessons**								
Costigan 2015 (9)	22.07	2.8999	21	22.63	1.9648	11	2.6%	-0.21 [-0.94, 0.52]
Costigan 2015 (10)	21.8	2.9462	19	22.63	1.8452	10	2.6%	-0.31 [-1.08, 0.46]
Eather 2015	21.5	3	49	21.7	1.3	34	3.1%	-0.08 [-0.52, 0.36]
Juric 2023 (11)	20.09	9.52	105	20.58	3.77	102	3.3%	-0.07 [-0.34, 0.21]
Parrish 2018 (12)	20.3	4.7	42	20.7	3.9	41	3.1%	-0.09 [-0.52, 0.34]
Tarp 2016 (13)	20.1	2.9	191	19.7	3.1	417	3.4%	0.13 [-0.04, 0.30]
Torbeyns 2017 (14)	19.9	3.2	21	20.5	3.5	23	2.9%	-0.18 [-0.77, 0.42]
Torbeyns 2017 (15)	19.9	3.2	21	20.5	3.5	23	2.9%	-0.18 [-0.77, 0.42]
Weeks 2008 (16)	20.6	3.3	22	21.9	5.6	15	2.8%	-0.29 [-0.95, 0.37]
Weeks 2008 (17)	20.3	3.5	21	20	2	23	2.9%	0.10 [-0.49, 0.70]
Weeks 2012 (18)	20.5	3.3	52	20.4	3.7	47	3.2%	0.03 [-0.37, 0.42]
**Subtotal (95% CI)**			**564**			**746**	**32.7%**	**0.01 [-0.11, 0.12]**
Heterogeneity: Tau² = 0.00; Chi² = 5.26, df = 10 (P = 0.87); I² = 0%								
Test for overall effect: Z = 0.12 (P = 0.90)								
**2.5.3 Educational Awareness Lessons**								
Haerens 2006 (19)	20.52	3.68	590	19.67	2.89	119	3.4%	0.24 [0.04, 0.44]
Haerens 2006 (20)	20.86	3.51	611	19.67	2.89	120	3.4%	0.35 [0.15, 0.54]
Haerens 2006 (21)	21.34	3.83	381	20.78	3.66	176	3.4%	0.15 [-0.03, 0.33]
Haerens 2006 (22)	21.66	3.68	118	20.78	3.66	176	3.3%	0.24 [0.01, 0.47]
Robbins 2006 (23)	22.55	4.26	4	20.64	4.5	9	1.9%	0.40 [-0.79, 1.59]
Rostami moez 2017 (24)	19.14	4.06	179	20.53	3.93	165	3.4%	-0.35 [-0.56, -0.13]
Shamah Lew 2012 (25)	45.3	108.1585	249	43.5	113.7667	249	3.4%	0.02 [-0.16, 0.19]
Shamah Lew 2012 (26)	54.3	124.182	249	60.8	123.6308	250	3.4%	-0.05 [-0.23, 0.12]
Young 2006 (27)	25.3	7.1	109	25.6	6.5	97	3.3%	-0.04 [-0.32, 0.23]
**Subtotal (95% CI)**			**2490**			**1361**	**28.9%**	**0.07 [-0.07, 0.22]**
Heterogeneity: Tau² = 0.03; Chi² = 31.10, df = 8 (P = 0.0001); I² = 74%								
Test for overall effect: Z = 1.00 (P = 0.32)								
**2.5.4 After School Activity**								
Borrestad 2012 (28)	18.8	0.8	24	18.6	0.8	22	2.9%	0.25 [-0.34, 0.83]
Kurnaz 2024 (29)	39.69	7.71	25	42.76	11.35	25	2.9%	-0.31 [-0.87, 0.25]
**Subtotal (95% CI)**			**49**			**47**	**5.8%**	**-0.04 [-0.58, 0.51]**
Heterogeneity: Tau² = 0.07; Chi² = 1.84, df = 1 (P = 0.18); I² = 46%								
Test for overall effect: Z = 0.14 (P = 0.89)								
**Total (95% CI)**			**5340**			**4028**	**100.0%**	**0.19 [-0.05, 0.43]**
Heterogeneity: Tau² = 0.44; Chi² = 874.45, df = 31 (P < 0.00001); I² = 96%								
Test for overall effect: Z = 1.54 (P = 0.12)								
Test for subgroup differences: Chi² = 3.70, df = 3 (P = 0.30), I² = 19.0%								

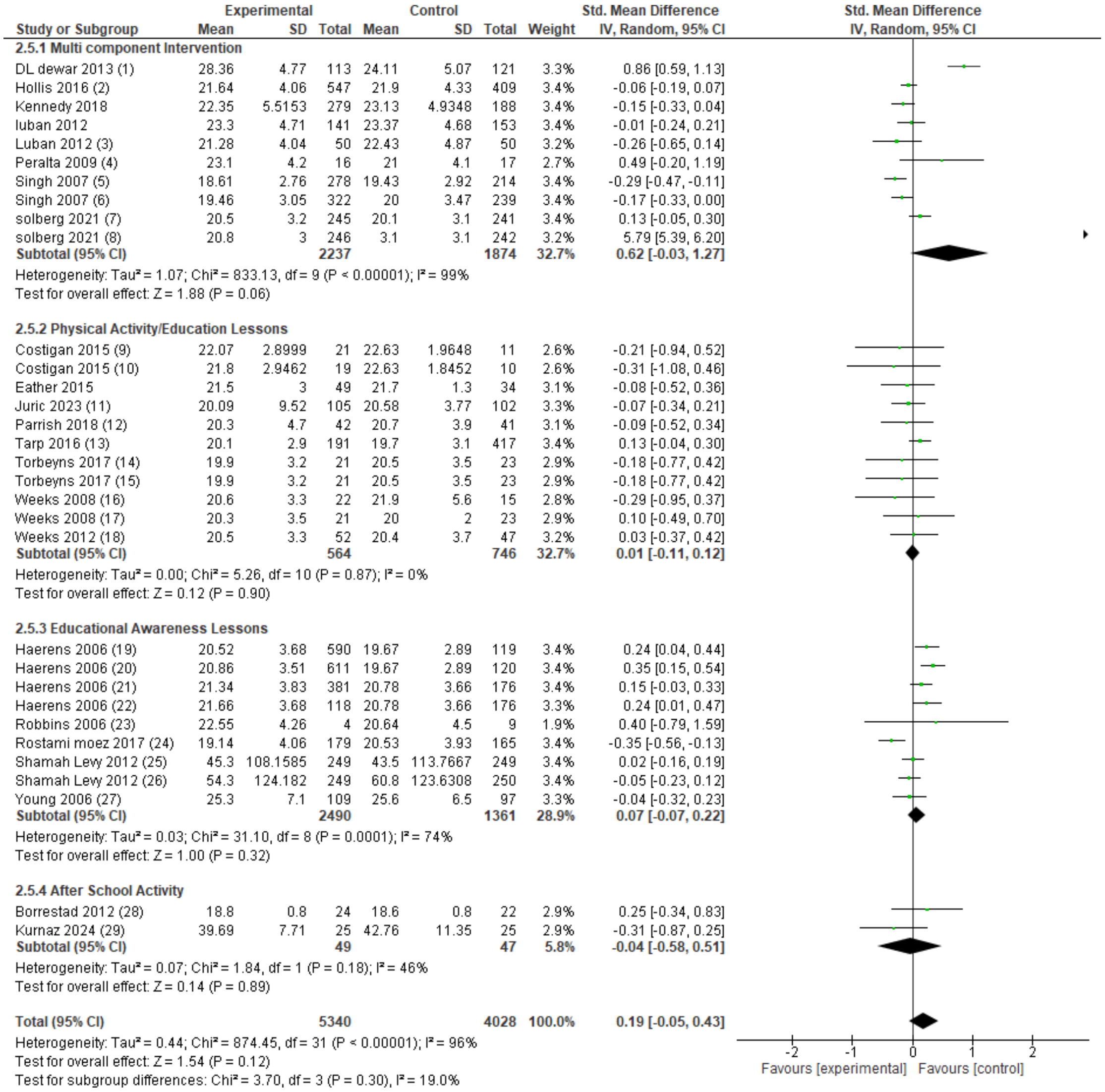

Figure 33: Forest plot - Intervention subgroup analysis

Dichotomous

Study or Subgroup	Experimental Events	Experimental Total	Control Events	Control Total	Weight	Risk Ratio M-H, Random, 95% CI
**2.7.1 Moderate to Vigorous Physical Activity**						
Dewar 2014	1	31	2	57	0.5%	0.92 [0.09, 9.74]
DL Dewar 2013	4	113	5	121	1.5%	0.86 [0.24, 3.11]
Guijarro-Romero 2020 (1)	21	42	5	19	3.8%	1.90 [0.84, 4.27]
Guijarro-Romero 2020 (2)	12	25	5	19	3.4%	1.82 [0.78, 4.29]
ha 2019	54	311	59	354	22.2%	1.04 [0.74, 1.46]
lonsdale 2013 (3)	25	60	10	27	7.6%	1.13 [0.63, 2.00]
lonsdale 2013 (4)	31	71	9	26	7.2%	1.26 [0.70, 2.28]
lonsdale 2013 (5)	28	77	10	27	7.6%	0.98 [0.55, 1.74]
Lubans 2018	118	529	96	542	43.1%	1.26 [0.99, 1.60]
**Subtotal (95% CI)**		**1259**		**1192**	**96.8%**	**1.20 [1.02, 1.41]**
Total events	294		201			
Heterogeneity: Tau² = 0.00; Chi² = 3.84, df = 8 (P = 0.87); I² = 0%						
Test for overall effect: Z = 2.20 (P = 0.03)						

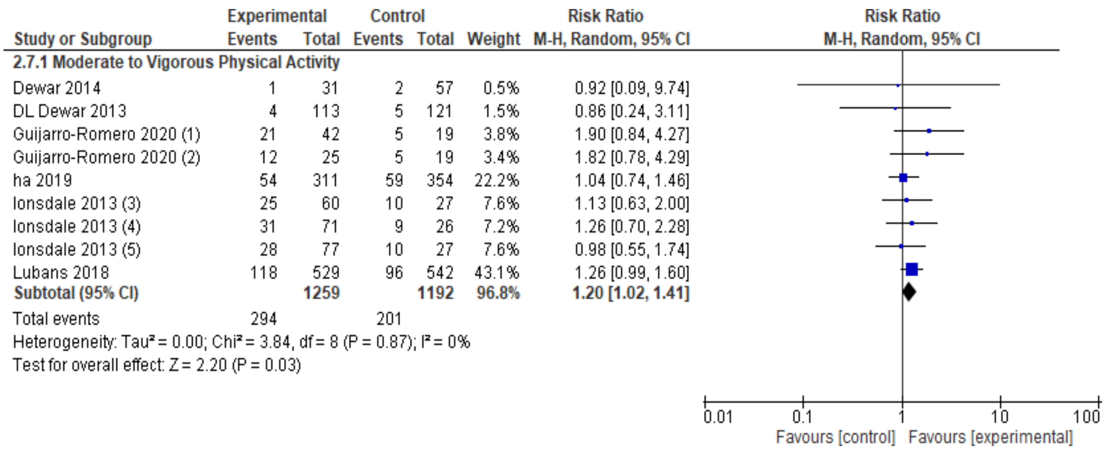

Figure 34: Forest Plot: Moderate to Vigorous Physical Activity

Study or Subgroup	Experimental Events	Experimental Total	Control Events	Control Total	Weight	Risk Ratio M-H, Random, 95% CI
**2.7.2 Vigorous Physical Activity**						
zhang 2020	1	24	2	27	0.5%	0.56 [0.05, 5.82]
**Subtotal (95% CI)**		**24**		**27**	**0.5%**	**0.56 [0.05, 5.82]**
Total events	1		2			
Heterogeneity: Not applicable						
Test for overall effect: Z = 0.48 (P = 0.63)						

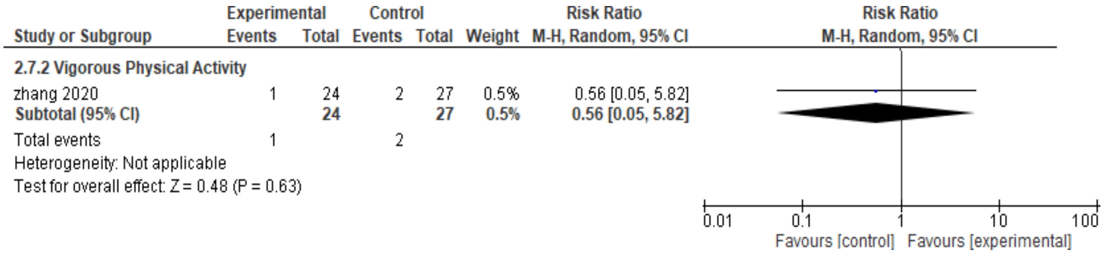

Figure 35: Forest Plot: Vigorous Physical Activity

Study or Subgroup	Experimental Events	Experimental Total	Control Events	Control Total	Weight	Risk Ratio M-H, Random, 95% CI
**2.7.3 Moderate Physical Activity**						
zhang 2020	1	24	2	27	0.5%	0.56 [0.05, 5.82]
**Subtotal (95% CI)**		**24**		**27**	**0.5%**	**0.56 [0.05, 5.82]**
Total events	1		2			
Heterogeneity: Not applicable						
Test for overall effect: Z = 0.48 (P = 0.63)						

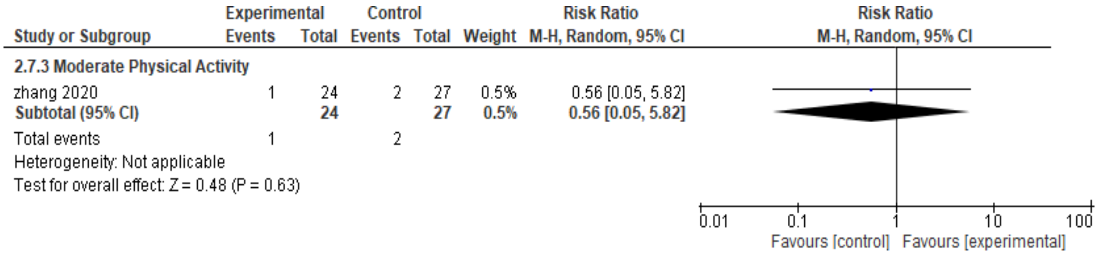

Figure 36: Forest Plot: Moderate Physical Activity

Study or Subgroup	Experimental Events	Experimental Total	Control Events	Control Total	Weight	Risk Ratio M-H, Random, 95% CI
**2.7.4 Light Physical Activity**						
zhang 2020	6	24	5	27	2.3%	1.35 [0.47, 3.86]
**Subtotal (95% CI)**		**24**		**27**	**2.3%**	**1.35 [0.47, 3.86]**
Total events	6		5			
Heterogeneity: Not applicable						
Test for overall effect: Z = 0.56 (P = 0.58)						
**Total (95% CI)**		**1331**		**1273**	**100.0%**	**1.19 [1.02, 1.40]**
Total events	302		210			
Heterogeneity: Tau² = 0.00; Chi² = 4.69, df = 11 (P = 0.95); I² = 0%						
Test for overall effect: Z = 2.18 (P = 0.03)						
Test for subgroup differences: Chi² = 0.85, df = 3 (P = 0.84), I² = 0%						

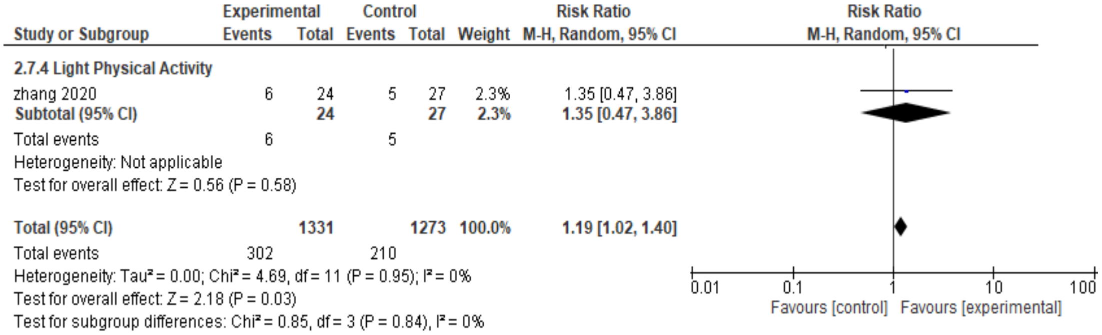

Figure 37: Forest Plot: Light Physical Activity

Study or Subgroup	Experimental Events	Experimental Total	Control Events	Control Total	Weight	Risk Ratio M-H, Random, 95% CI
**2.6.5 High Income Countries**						
Dewar 2014	1	31	2	57	0.5%	0.92 [0.09, 9.74]
DL Dewar 2013	4	113	5	121	1.5%	0.86 [0.24, 3.11]
Guijarro-Romero 2020 (1)	12	25	5	19	3.4%	1.82 [0.78, 4.29]
Guijarro-Romero 2020 (2)	21	42	5	19	3.8%	1.90 [0.84, 4.27]
ha 2019	54	311	59	354	22.2%	1.04 [0.74, 1.46]
lonsdale 2013 (3)	25	60	10	27	7.6%	1.13 [0.63, 2.00]
lonsdale 2013 (4)	31	71	9	26	7.2%	1.26 [0.70, 2.28]
lonsdale 2013 (5)	28	77	10	27	7.6%	0.98 [0.55, 1.74]
Lubans 2018	118	529	96	542	43.1%	1.26 [0.99, 1.60]
**Subtotal (95% CI)**		**1259**		**1192**	**96.8%**	**1.20 [1.02, 1.41]**
Total events	294		201			

Heterogeneity: Tau² = 0.00; Chi² = 3.84, df = 8 (P = 0.87); I² = 0%
Test for overall effect: Z = 2.20 (P = 0.03)

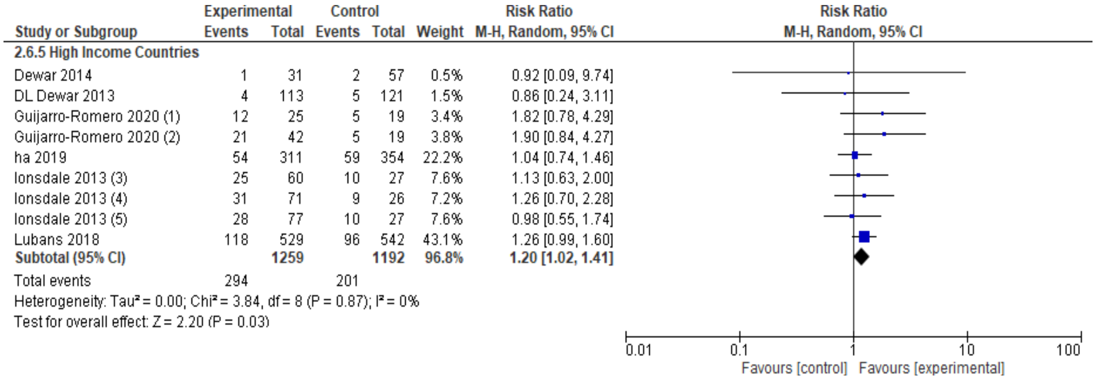

Figure 38: Forest Plot - Physical Activity subgroup analysis - High Income Countries

Study or Subgroup	Experimental Events	Experimental Total	Control Events	Control Total	Weight	Risk Ratio M-H, Random, 95% CI
**2.6.6 Upper Middle Income Countries**						
zhang 2020 (6)	1	24	2	27	0.5%	0.56 [0.05, 5.82]
zhang 2020 (7)	1	24	2	27	0.5%	0.56 [0.05, 5.82]
zhang 2020 (8)	6	24	5	27	2.3%	1.35 [0.47, 3.86]
**Subtotal (95% CI)**		**72**		**81**	**3.2%**	**1.05 [0.43, 2.55]**
Total events	8		9			
Heterogeneity: Tau² = 0.00; Chi² = 0.78, df = 2 (P = 0.68); I² = 0%						
Test for overall effect: Z = 0.11 (P = 0.92)						
**Total (95% CI)**		**1331**		**1273**	**100.0%**	**1.19 [1.02, 1.40]**
Total events	302		210			

Heterogeneity: Tau² = 0.00; Chi² = 4.69, df = 11 (P = 0.95); I² = 0%
Test for overall effect: Z = 2.18 (P = 0.03)
Test for subgroup differences: Chi² = 0.08, df = 1 (P = 0.77), I² = 0%

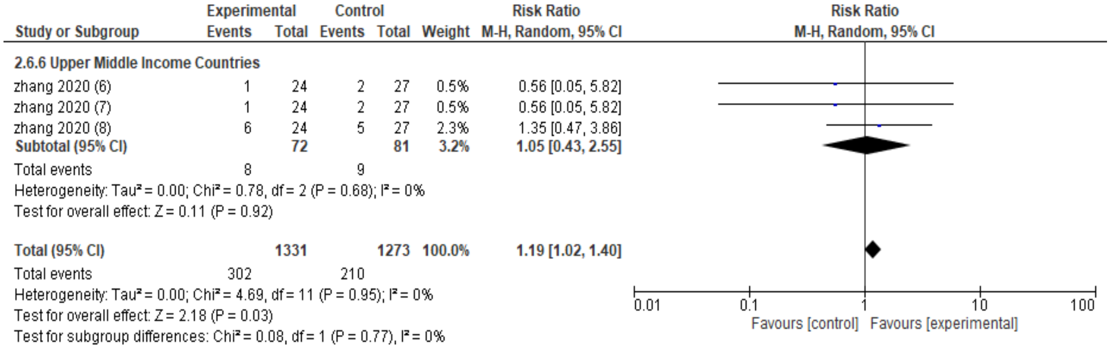

Figure 39: Forest Plot - Physical Activity subgroup analysis - Upper Middle-Income Countries

Study or Subgroup	Experimental Events	Experimental Total	Control Events	Control Total	Weight	Risk Ratio M-H, Random, 95% CI
**2.5.7 0-6 months**						
Guijarro-Romero 2020 (1)	12	25	5	19	3.4%	1.82 [0.78, 4.29]
Guijarro-Romero 2020 (2)	21	42	5	19	3.8%	1.90 [0.84, 4.27]
ha 2019	54	311	59	354	22.2%	1.04 [0.74, 1.46]
lonsdale 2013 (3)	25	60	10	27	7.6%	1.13 [0.63, 2.00]
lonsdale 2013 (4)	31	71	9	26	7.2%	1.26 [0.70, 2.28]
lonsdale 2013 (5)	28	77	10	27	7.6%	0.98 [0.55, 1.74]
zhang 2020 (6)	1	24	2	27	0.5%	0.56 [0.05, 5.82]
zhang 2020 (7)	1	24	2	27	0.5%	0.56 [0.05, 5.82]
zhang 2020 (8)	6	24	5	27	2.3%	1.35 [0.47, 3.86]
**Subtotal (95% CI)**		**658**		**553**	**55.0%**	**1.16 [0.93, 1.43]**
Total events	179		107			

Heterogeneity: Tau² = 0.00; Chi² = 4.12, df = 8 (P = 0.85); I² = 0%
Test for overall effect: Z = 1.33 (P = 0.18)

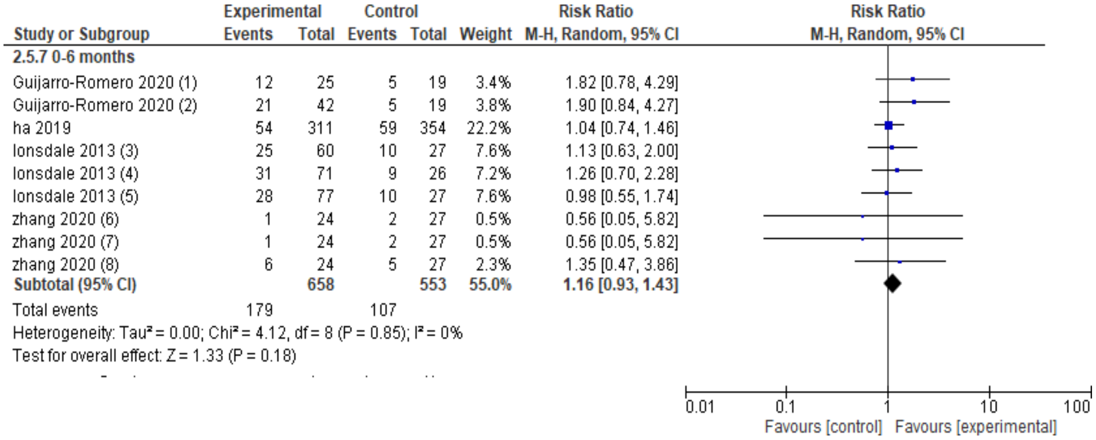

Figure 40: Forest Plot - Physical Activity subgroup analysis - 0-6 months follow up

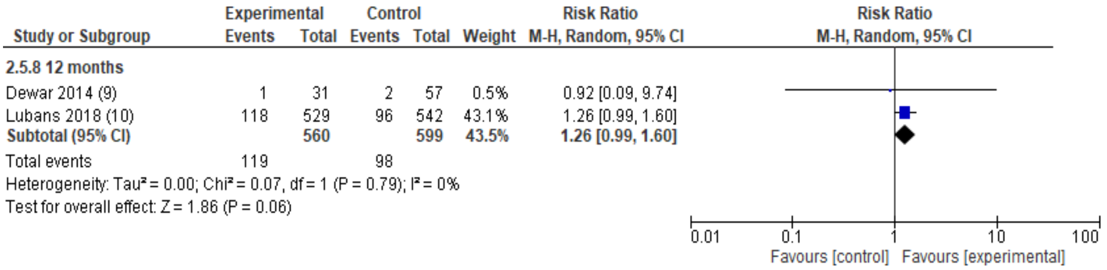

Figure 41: Forest Plot - Physical Activity subgroup analysis - 12 months follow up

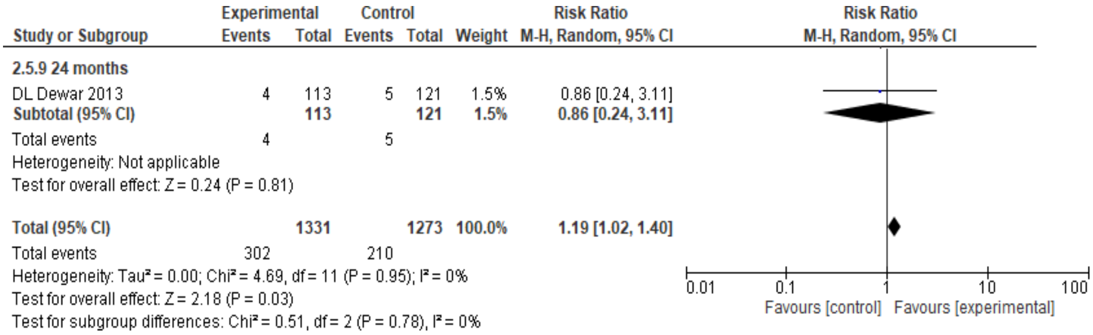

Figure 42: Forest Plot - Physical Activity subgroup analysis - 24 months follow up

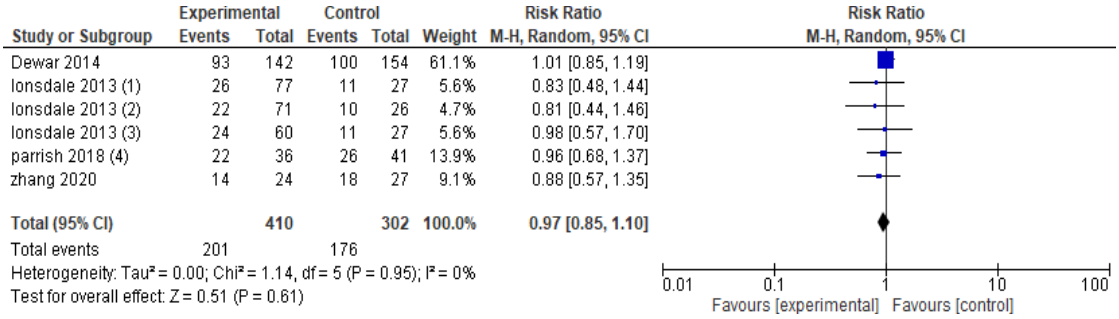

Figure 43: Forest Plot - Dichotomous Outcome - Sedentary time

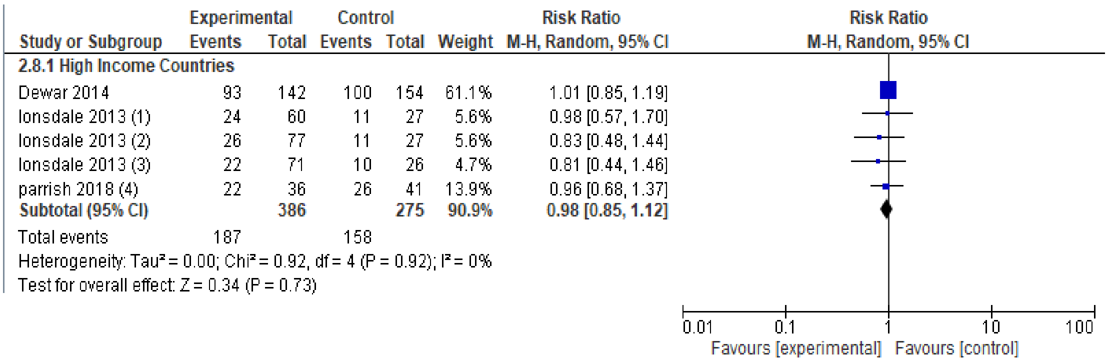

Figure 44: Forest Plot - Sedentary time subgroup analysis - High Income Countries

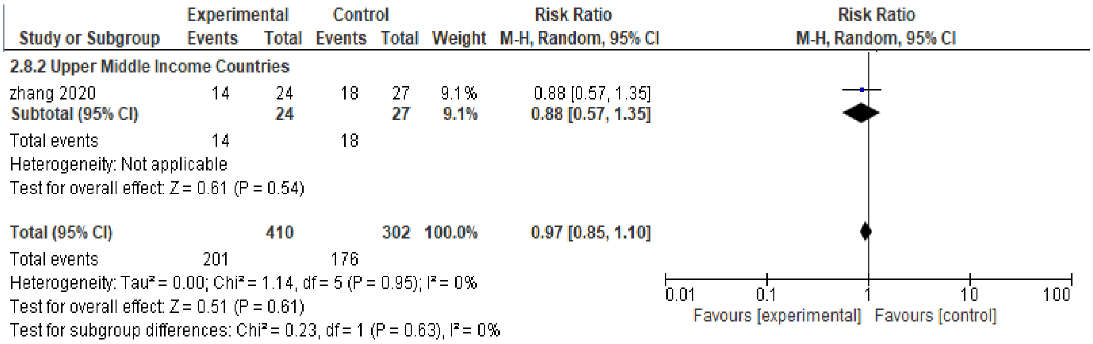

Figure 45: Forest Plot - Sedentary time subgroup analysis - Upper Middle Income Countries

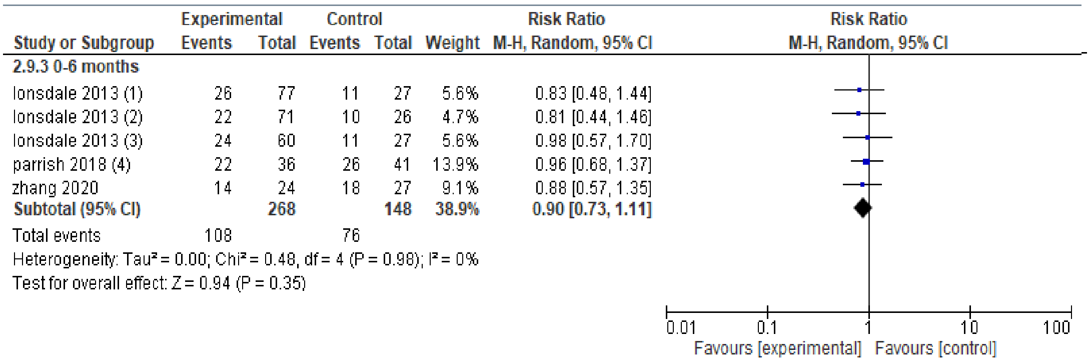

Figure 46: Forest Plot - Sedentary time subgroup analysis - 0-6 months follow up

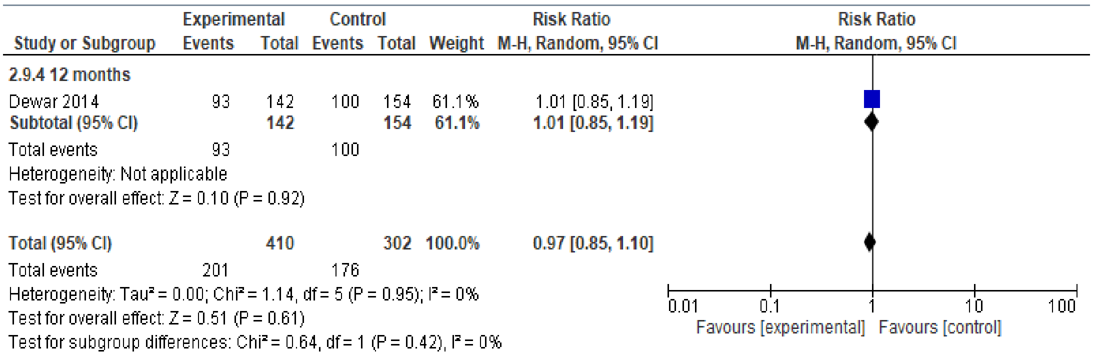

Figure 47: Forest Plot - Sedentary time subgroup analysis - 12 months follow up

Funnel Plots

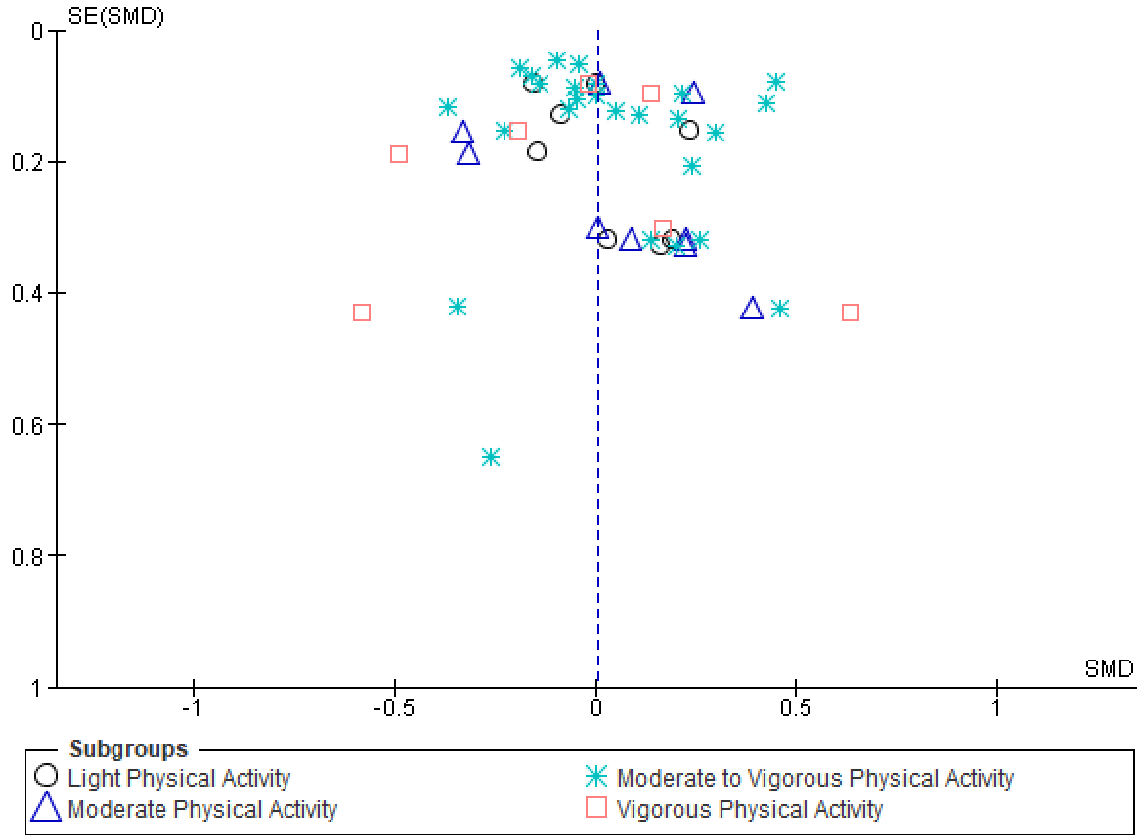

Figure 1Funnel plot of comparison:Physical Activity (Intensity).

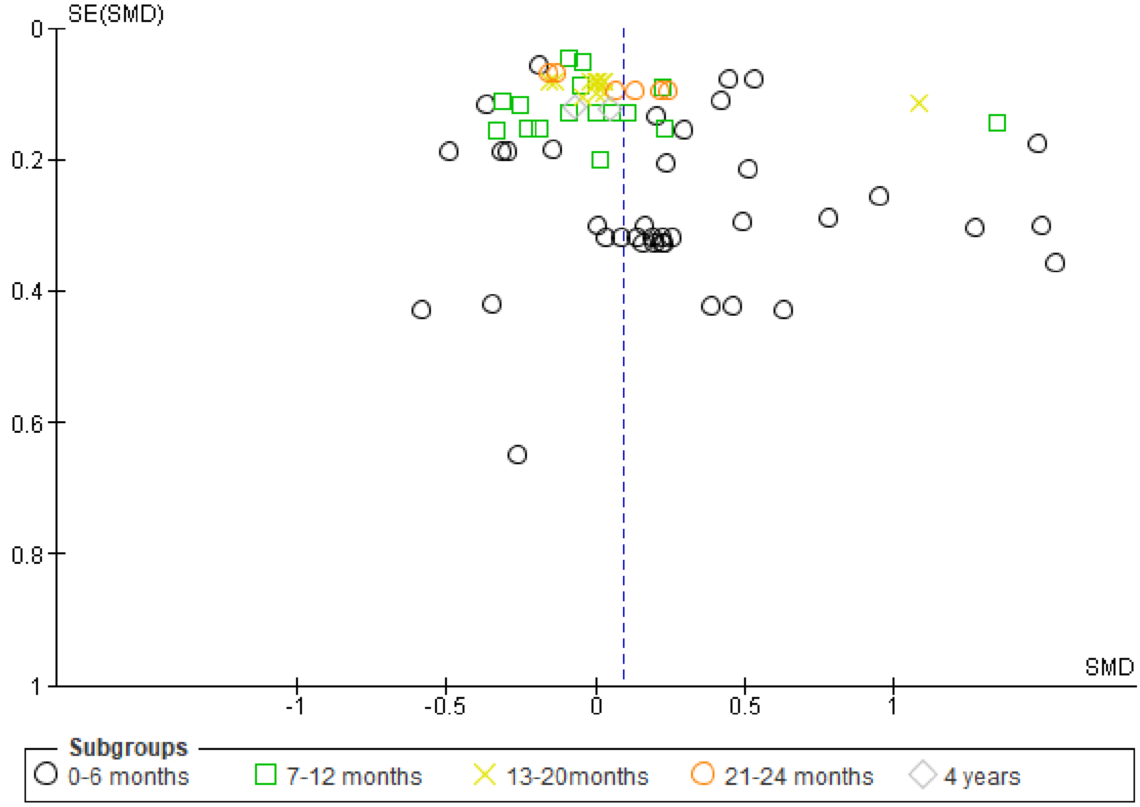

Figure 2Funnel plot of comparison: Physical Activity-Duration of Followup.

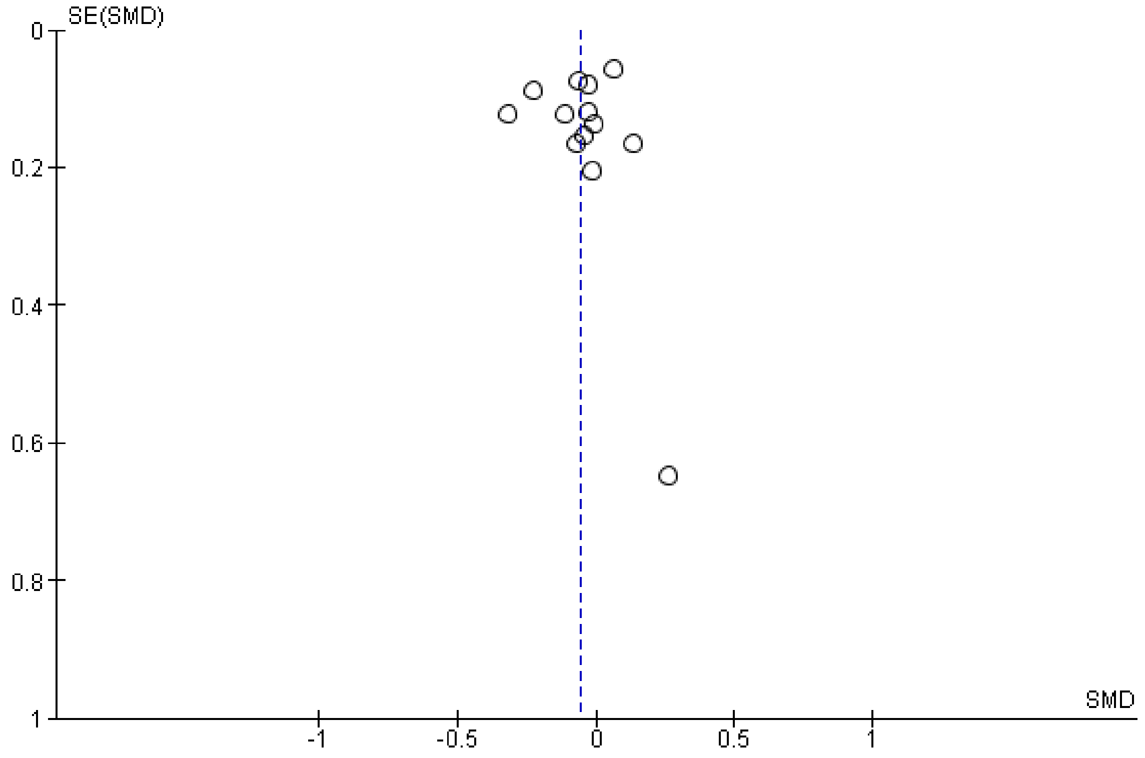

Figure 3 Sedentary Behavior

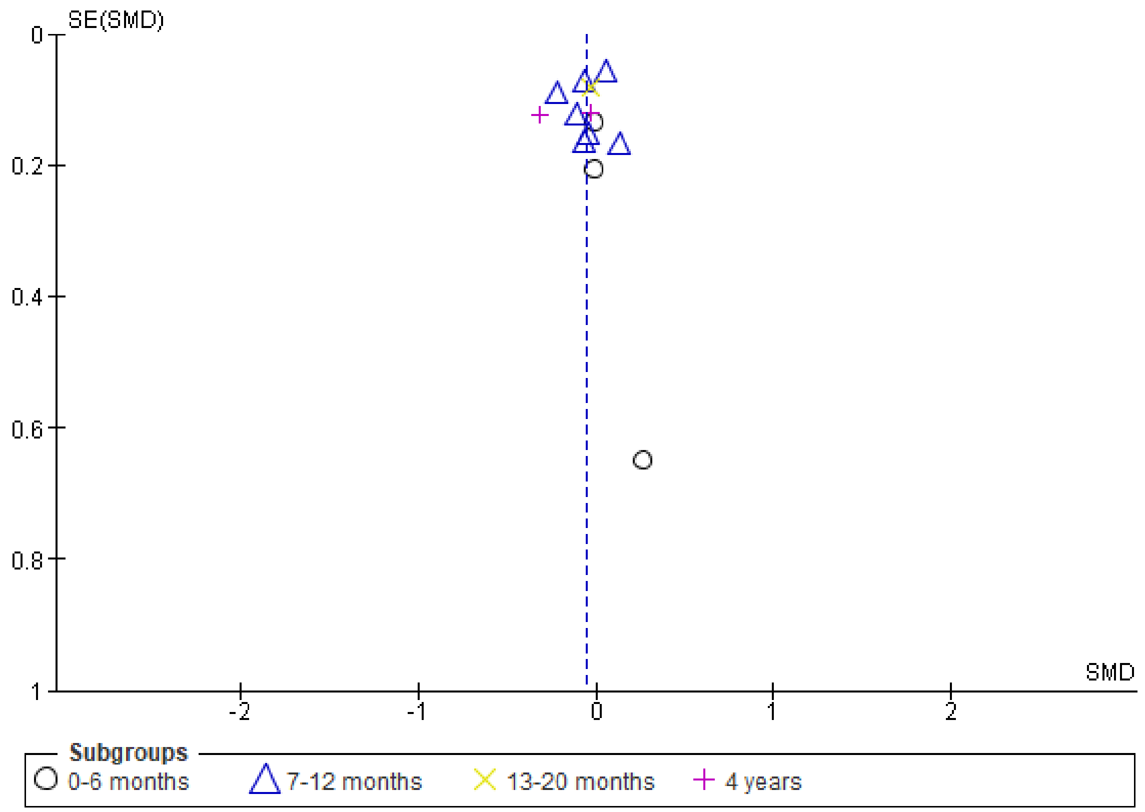

Figure 4 Sedentary Behavior-Duration of Followup.

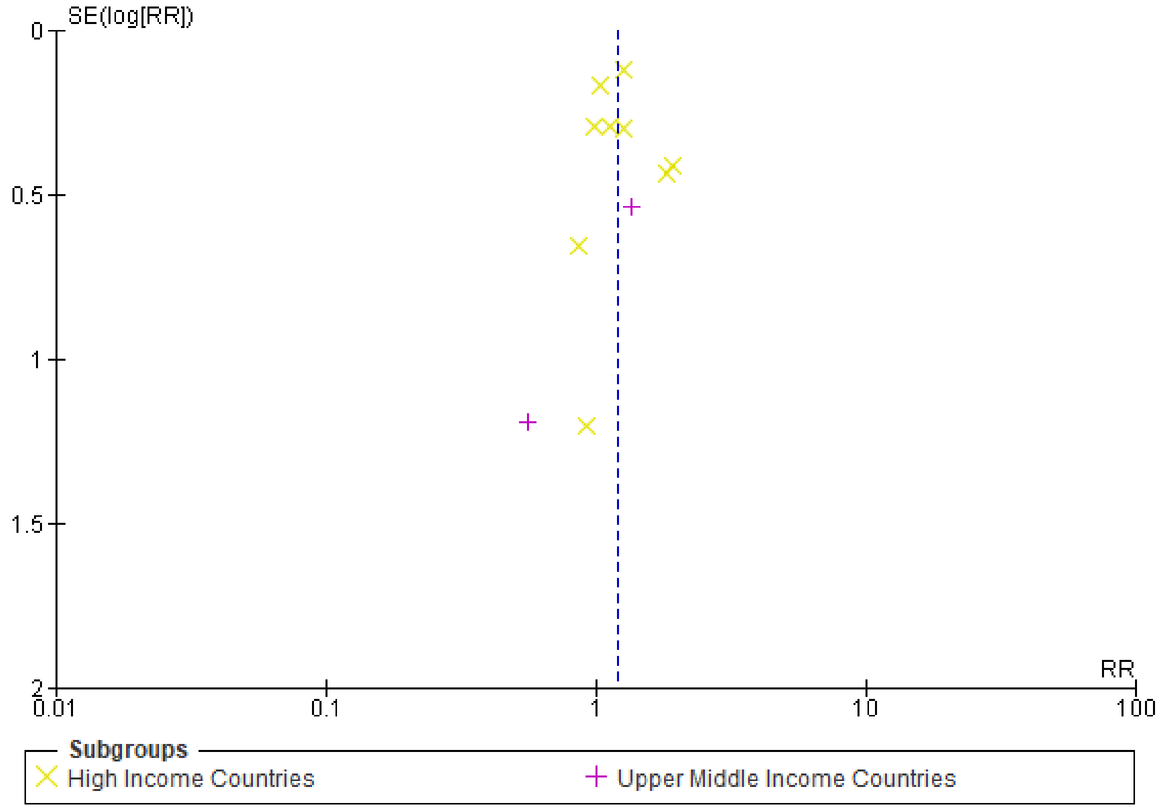

Figure 5 Physical Activity-Country Income Group

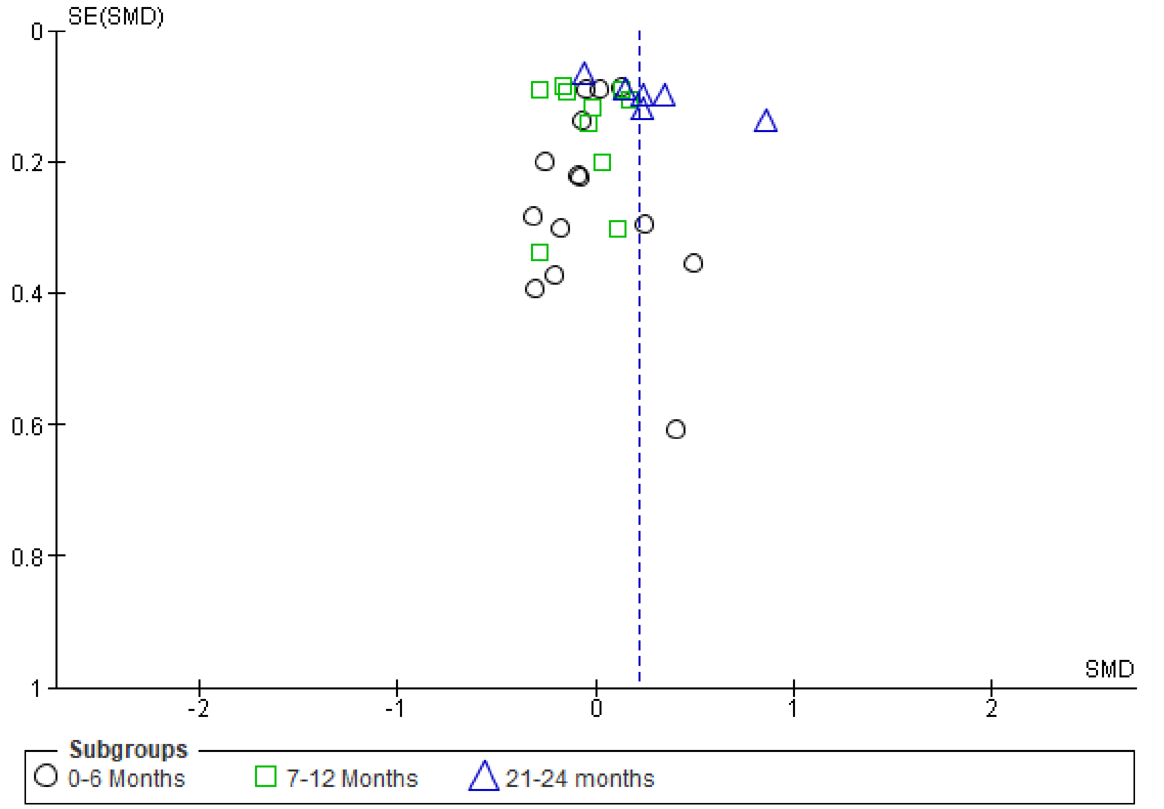

Figure 6 BMI-Duration of Followup

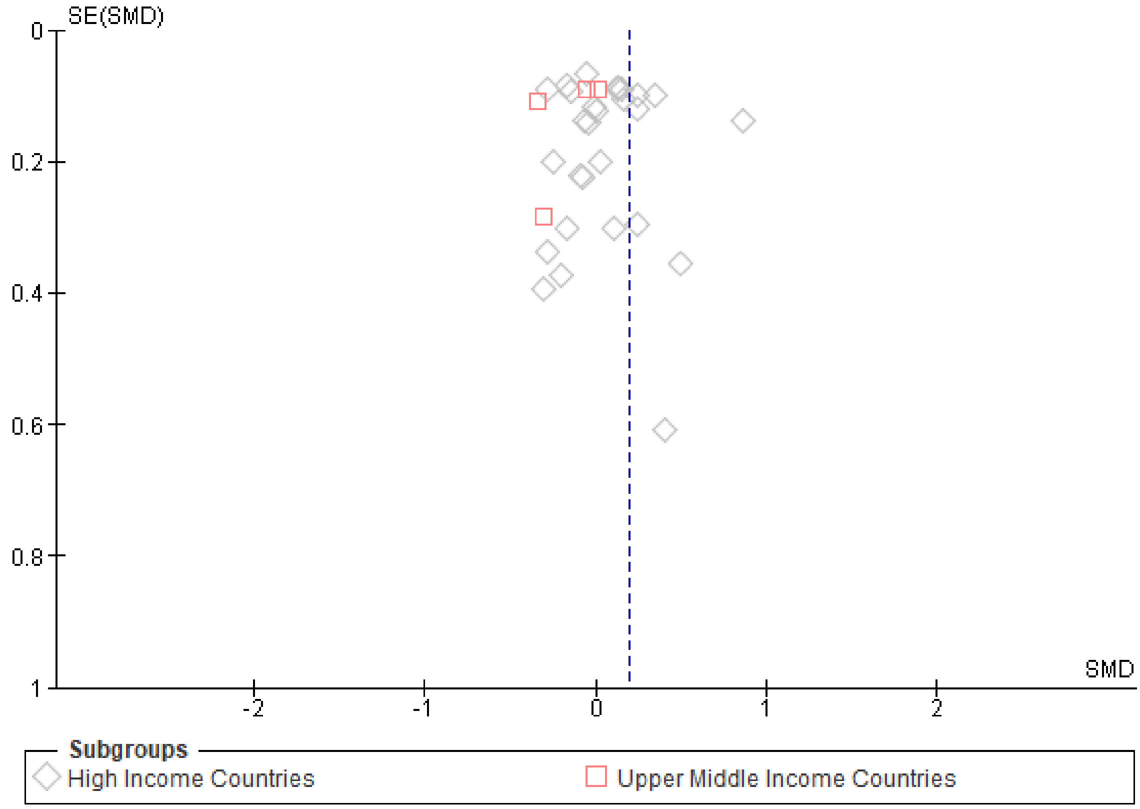

Figure 7 BMI-Country Income Classification

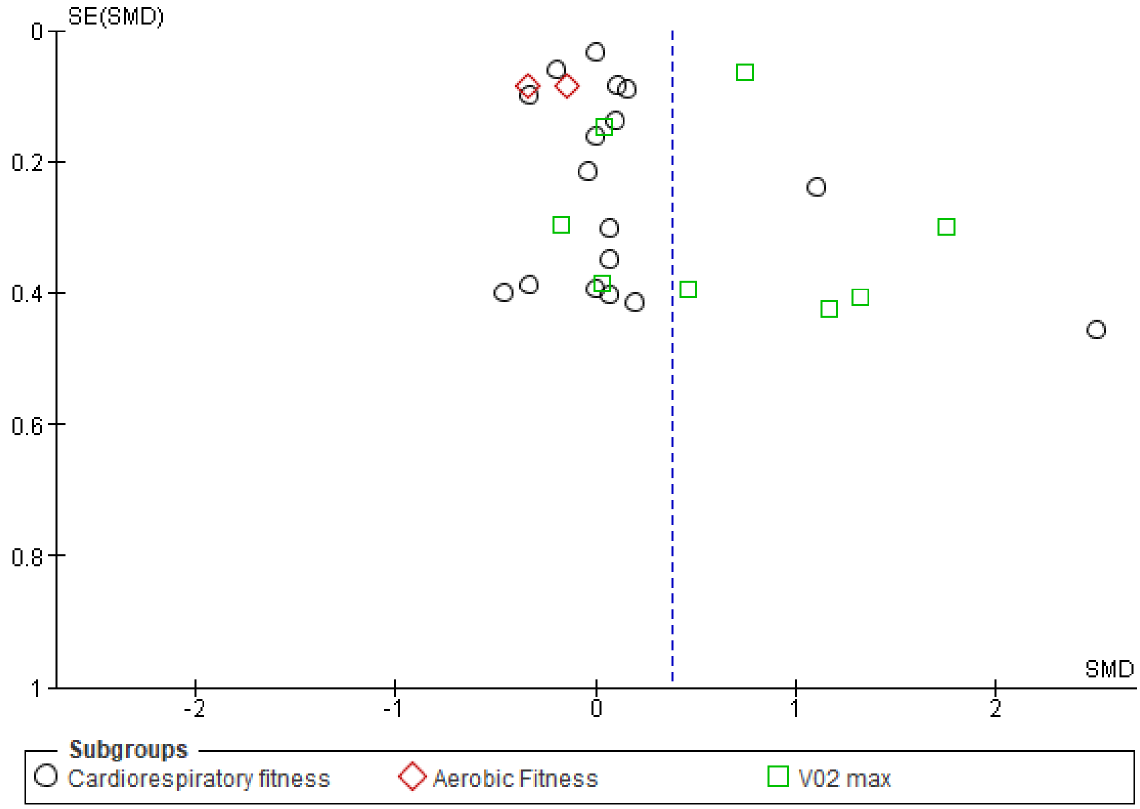

Figure 8 Physical Fitness

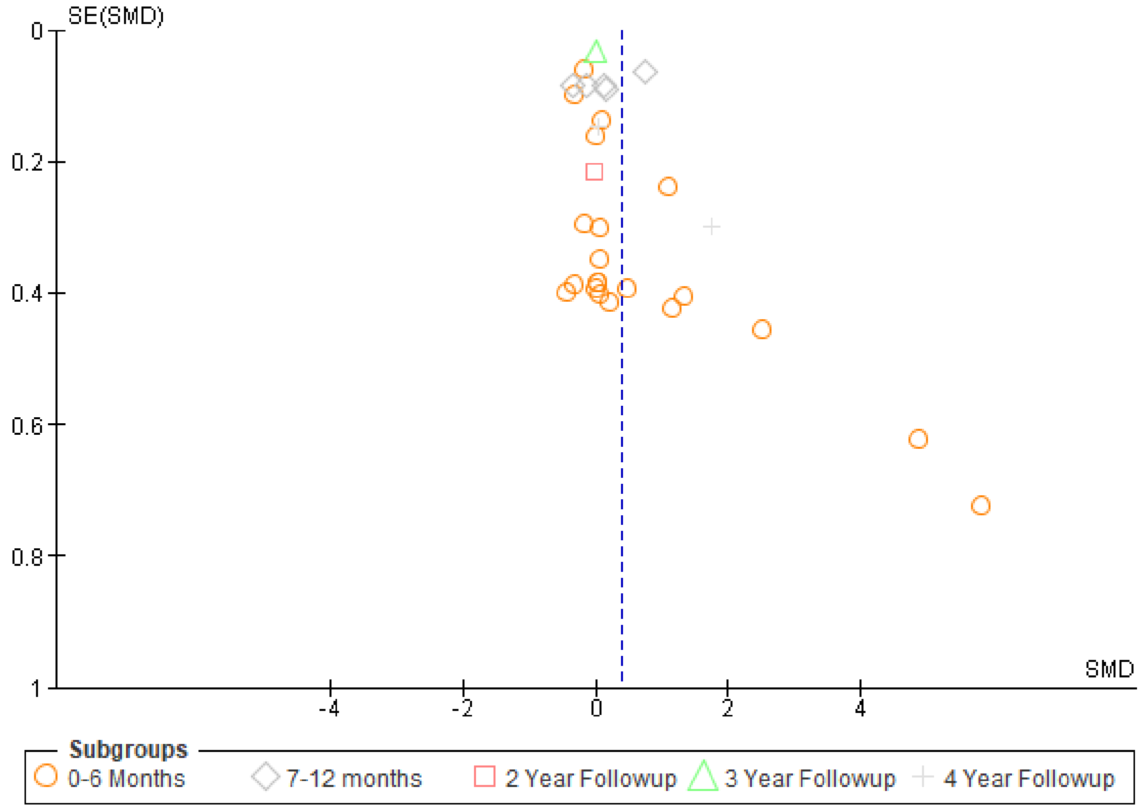

Figure 9 Physical Fitness-Duration of Followup

Sensitivity Analysis

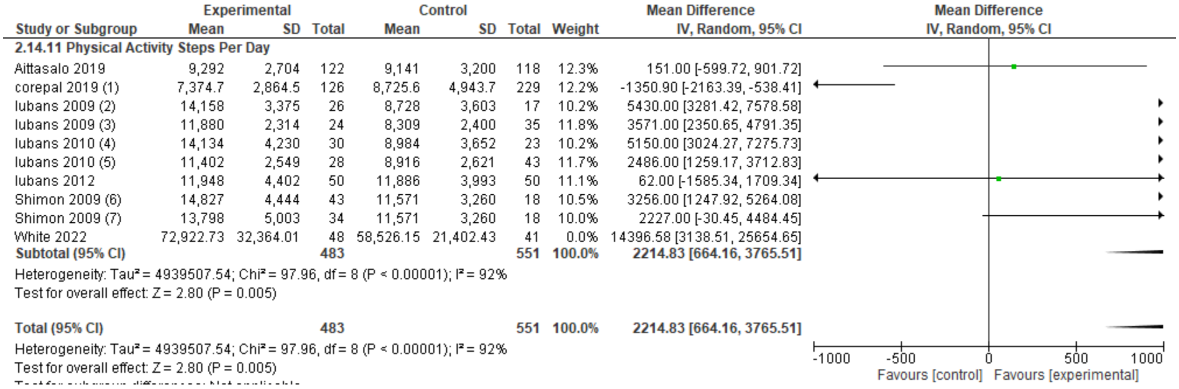

Study or Subgroup	Experimental Mean	Experimental SD	Experimental Total	Control Mean	Control SD	Control Total	Weight	Mean Difference IV, Random, 95% CI
**2.14.11 Physical Activity Steps Per Day**								
Aittasalo 2019	9,292	2,704	122	9,141	3,200	118	12.3%	151.00 [-599.72, 901.72]
corepal 2019 (1)	7,374.7	2,864.5	126	8,725.6	4,943.7	229	12.2%	-1350.90 [-2163.39, -538.41]
lubans 2009 (2)	14,158	3,375	26	8,728	3,603	17	10.2%	5430.00 [3281.42, 7578.58]
lubans 2009 (3)	11,880	2,314	24	8,309	2,400	35	11.8%	3571.00 [2350.65, 4791.35]
lubans 2010 (4)	14,134	4,230	30	8,984	3,652	23	10.2%	5150.00 [3024.27, 7275.73]
lubans 2010 (5)	11,402	2,549	28	8,916	2,621	43	11.7%	2486.00 [1259.17, 3712.83]
lubans 2012	11,948	4,402	50	11,886	3,993	50	11.1%	62.00 [-1585.34, 1709.34]
Shimon 2009 (6)	14,827	4,444	43	11,571	3,260	18	10.5%	3256.00 [1247.92, 5264.08]
Shimon 2009 (7)	13,798	5,003	34	11,571	3,260	18	10.0%	2227.00 [-30.45, 4484.45]
White 2022	72,922.73	32,364.01	48	58,526.15	21,402.43	41	0.0%	14396.58 [3138.51, 25654.65]
**Subtotal (95% CI)**			**483**			**551**	**100.0%**	**2214.83 [664.16, 3765.51]**
Heterogeneity: Tau² = 4939507.54; Chi² = 97.96, df = 8 (P < 0.00001); I² = 92%								
Test for overall effect: Z = 2.80 (P = 0.005)								
**Total (95% CI)**			**483**			**551**	**100.0%**	**2214.83 [664.16, 3765.51]**
Heterogeneity: Tau² = 4939507.54; Chi² = 97.96, df = 8 (P < 0.00001); I² = 92%								
Test for overall effect: Z = 2.80 (P = 0.005)								
[illegible]								

Step per Day

Study or Subgroup	Experimental Mean	Experimental SD	Experimental Total	Control Mean	Control SD	Control Total	Weight	Mean Difference IV, Random, 95% CI
**2.15.14 Physical Activity: Counts per minute**								
Aittasalo 2019 (1)	239.3	49.7	122	239.2	60.6	118	7.8%	0.10 [-13.95, 14.15]
Bergh 2012 (2)	569.8	251.6	215	563.8	255.3	485	5.4%	6.00 [-34.59, 46.59]
carlin 2018 (3)	134.69	24.28	52	142.67	28.64	65	8.1%	-7.98 [-17.57, 1.61]
Dudley 2010 (4)	82.1	17.6	17	75	37	21	7.6%	7.10 [-10.80, 25.00]
grydeland 2013	570	252	215	564	255	485	5.4%	6.00 [-34.62, 46.62]
ha 2019	80,033	31,886	311	64,101	28,538	354	0.0%	15932.00 [11306.40, 20557.60]
jago 2015 (5)	450.98	263.89	124	405.04	228.96	145	3.9%	45.94 [-13.61, 105.49]
jago 2015 (6)	478.75	144.94	246	446.83	137.81	262	7.0%	31.92 [7.29, 56.55]
Kolle 2020 (7)	497	298.6269	317	489	206.4054	144	4.9%	8.00 [-39.09, 55.09]
Kolle 2020 (8)	476	284.158	229	234	36.4245	144	5.8%	242.00 [204.72, 279.28]
lubans 2012 (9)	328.4	91.5	141	359.2	140.4	153	6.8%	-30.80 [-57.69, -3.91]
miller 2015 (10)	19.04	12.2554	97	0.78	12.3365	71	8.3%	18.26 [14.49, 22.03]
smith 2014 (11)	515	33.51	120	473	28.58	120	8.2%	42.00 [34.12, 49.88]
smith 2014 (12)	410	54.85	83	387	47.13	83	7.7%	23.00 [7.44, 38.56]
Sutherland 2016	460.2	174.8228	245	448.5	165.3505	191	6.3%	11.70 [-20.38, 43.78]
toftager 2014 (13)	408	167	376	433	206	421	6.9%	-25.00 [-50.93, 0.93]
**Subtotal (95% CI)**			**2599**			**2908**	**100.0%**	**22.56 [6.50, 38.62]**
Heterogeneity: Tau² = 804.41; Chi² = 236.76, df = 14 (P < 0.00001); I² = 94%								
Test for overall effect: Z = 2.75 (P = 0.006)								
**Total (95% CI)**			**2599**			**2908**	**100.0%**	**22.56 [6.50, 38.62]**
Heterogeneity: Tau² = 804.41; Chi² = 236.76, df = 14 (P < 0.00001); I² = 94%								
Test for overall effect: Z = 2.75 (P = 0.006)								
[illegible]								

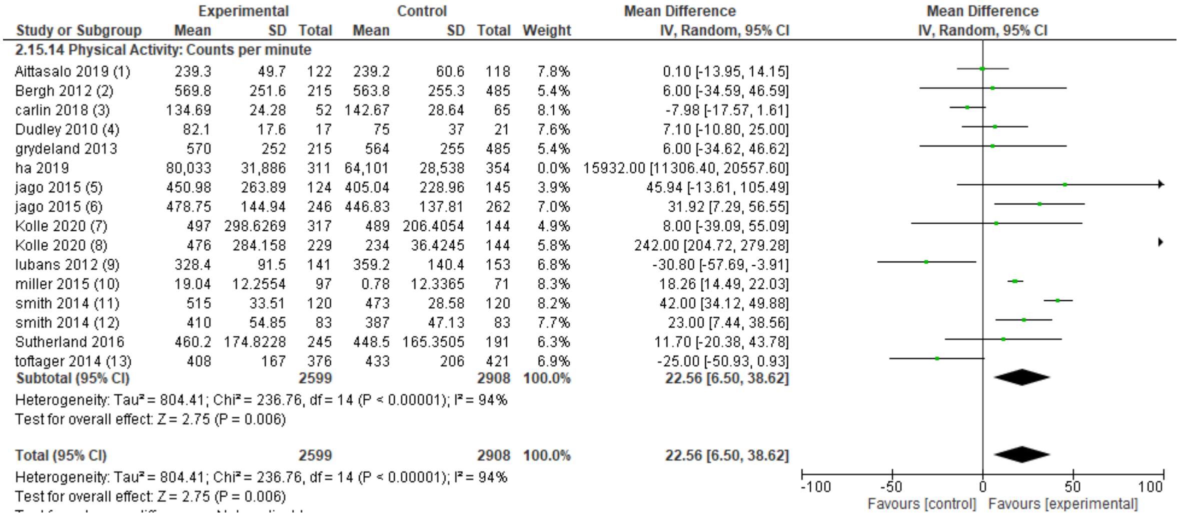

Counts per minute

"Strategies to Promote Adolescent Physical Activity in School Setting: A systematic review and meta-analysis."

Research Protocol

Contents

1. Introduction 3
1.1 Background 3
1.2. Rationale for the Study 4
1.3. Research Question Framed Using PICO 5
2.1. Exclusion and Inclusion Criteria for considering studies for this review. 6
2.1.1. Types of studies: 6
2.1.2. Types of participants: 6
2.1.3. Types of intervention/exposure: 6
2.1.4. Types of outcomes: 6
2.2. Eligibility Criteria (PICO framework) 7
2.3. Search methods for identification of studies. 8
2.3.1. Electronic search and search strategy: 8
2.3.2. Searching other sources: 8
2.4. Data collection and analysis: 8
2.4.1. Selection of Studies: 8
2.5. Data Extraction and Management 9
2.5.1. Quality Assessment of Included Studies: 10
2.5.3. Measures of Treatment Effect: 10
2.5.4. GRADE and Summary of Finding Tables 10
2.5.5. Sub-Group Analysis 11
2.6. Ethical Considerations 11
3. Annexure 12
4.References 14

1. Introduction

1.1 Background

The World Health Organization (WHO) defines physical activity as any bodily movement that requires energy expenditure and is produced by skeletal muscles. This encompasses all types of movement, whether during leisure time, for transportation, or as part of one's work. Both moderate- and vigorous-intensity physical activities have positive effects on health. (1)

Common ways to engage in physical activity include walking, cycling, participating in sports, recreational activities, and play. These activities can be enjoyed by people of all skill levels and are beneficial for overall well-being. (1)

Regular physical activity has been shown to be effective in preventing and managing noncommunicable diseases such as heart disease, stroke, diabetes, and various cancers. (2) It also aids in preventing hypertension, maintaining a healthy body weight, and can enhance mental health, quality of life, and overall well-being. (1)(3)

Children and adolescents between the ages of 5 and 17 are recommended to engage in at least 60 minutes per day of moderate-to-vigorous intensity physical activity, primarily aerobic, spread out over the week. (1)(4) Examples including brisk walking, jogging, or running, cycling, swimming, jumping rope, dancing, hiking, aerobic exercise classes (e.g., Zumba, step aerobics), playing sports such as soccer, basketball, tennis, or volleyball, rollerblading, or skateboarding. It's advised that they include vigorous-intensity aerobic activities, as well as exercises that promote muscle and bone strength, on at least 3 days per week. (1)(4) Additionally, reducing sedentary behaviors, especially recreational screen time, is important. (5)

Physical inactivity stands as a primary contributor to the prevalence of noncommunicable diseases (NCDs) and mortality globally. (6) It escalates the likelihood of developing cancer, cardiovascular conditions, stroke, and diabetes by approximately 20–30%. Statistics indicate that four to five million deaths annually could be prevented with more active lifestyles. (7)

Remarkably, one in four adults and an alarming four out of five adolescents fail to meet the recommended levels of physical activity. (7) Notably, there exists a significant disparity between genders, with women and girls generally exhibiting lower activity levels compared to their male counterparts, thereby exacerbating health disparities. (8)

In 2016, a global study on global trends in insufficient physical activity among adolescents, collected data from 298 school-based surveys conducted across 146 countries (Including HICs, LMICS), territories, and areas, involving 1.6 million students aged 11–17 years, revealed that 81.0% (with a 95% uncertainty interval of 77.8–87.7) of students in this age group were not engaging in sufficient physical activity. This lack of physical activity was more prevalent among girls, with 84.7% (with an uncertainty interval of 83.0–88.2) being insufficiently active, compared to boys at 77.6% (with an uncertainty interval of 76.1–80.4). While there was a significant decrease in the prevalence of insufficient physical activity among boys between 2001 and 2016, from 80.1% in 2001, the trend remained stagnant for girls during the same period, with no significant change from 85.1% in 2001.

There was no consistent pattern observed based on country income groups. In 2016, the prevalence of insufficient physical activity was 84.9% in low-income countries (LICs), 79.3% in lower-middle-income countries(LMICs), 83.9% in upper-middle-income countries(UMICs), and 79.4% in high-income countries(HICs). Notably, high-income Asia Pacific had the highest prevalence of insufficient physical activity for both boys (89.0%) and girls (95.6%), while high-income Western countries had the lowest prevalence for boys (72.1%), and South Asia for girls (77.5%). Moreover, in 2016, 27 countries recorded a prevalence of insufficient physical activity of 90% or more among girls, whereas this was observed in only two countries for boys. (9)

This decline in physical activity is more pronounced in schools and colleges settings where academic pressures and lack of infrastructure often overshadow the importance of physical activity (10). Factors contributing to this decline include increased academic workload, social changes, and a lack of appropriately designed physical education programs in schools and colleges (11). Another study focusing on LMICs suggest factors including demographic aspects (such as gender and age), policy-related factors (like participation in physical education classes), socio-environmental factors (such as parental support, friends, and bullying), and health behaviour factors (including fruit and vegetable consumption). (12) Moreover, the transition from primary to secondary education often coincides with reduced opportunities and motivation for physical activity (13).

During adolescence, individuals undergo significant physical, emotional, and cognitive changes. Engaging in regular physical activity during this stage is associated with improved cardiorespiratory and muscular fitness, bone health, cardiovascular and metabolic disease risk reduction, and positive mental health outcomes (14). Furthermore, physical activity habits developed during adolescence can track into adulthood, influencing long-term health (15)

Schools and colleges are recognized as strategic settings for promoting physical activity, given the significant amount of time adolescents spend in them (16)(17). The school and college setting presents a unique opportunity for interventions due to its structured environment and the potential to reach a large adolescent population. (18) Effective school and college-based interventions can provide equitable opportunities for adolescents to engage in physical activity, regardless of their socio-economic background (19).

1.2. Rationale for the Study

The need to investigate and implement effective strategies and interventions to promote physical activity in adolescents, specifically within school and college environments, forms the basis of this research.

Implementing school and college-based strategies that target all students guarantees that every student is exposed to the intervention, thereby expanding the reach of these interventions. Enhancing physical activity is a crucial public health and health promotion tactic aimed at enhancing child health (20).

Previous systematic reviews have examined strategies and interventions aimed at promoting physical activity among adolescents in school and college settings. For example, a systematic review done in 2012 concluded that school and college-based physical activity interventions can be effective in promoting health and physical activity in students, but further research is needed to understand the underlying mechanisms and the optimal design of such interventions. (18)

Another systematic review and meta-analysis examined school and college-based physical activity interventions for children and adolescents. The results indicate that while these interventions may improve physical fitness and have a small impact on BMI, they do not significantly increase moderate

to vigorous physical activity or reduce sedentary time. The authors recommend further research to understand the mechanisms behind these effects and optimize intervention design. (13) Similarly, a review by Kriemler et al. (2011) highlighted the importance of multi-component interventions involving changes to the school and college environment, curriculum, and family involvement in promoting physical activity among youth. The authors recommend further research to understand the mechanisms behind these effects and optimize intervention design. (21)

One more review suggested that school and college-based health education interventions have the potential to lower BMI towards a healthier range in adolescents. Interventions should target the biological, psychosocial, environmental, and behavioral influences on diet and physical activity. Future studies should consider the long-term effects, sustainability, and implementation of such interventions at a population level. (22)

While these systematic reviews have provided valuable insights, there is a need for an updated synthesis of the literature, particularly focusing on LMICs. Existing evidence predominantly comes from high-income countries (HICs), and there is limited research examining the effectiveness of strategies and interventions to promote adolescent physical activity in LMIC settings. As suggested by a study focusing on LMICs that only 3.1% of the studies mentioned in previous reviews on PA interventions for children and adolescents were from LMICs. (23) The authors concluded that most of the previous evidence on PA interventions for children and adolescents has limited extrapolation to the LMIC context. They also suggested priorities of implementation and practical implication that can be used in public policies for PA promotion in LMIC (23). Given the unique socio-cultural, economic, and infrastructural contexts of LMICs, strategies that have been effective in HICs may not necessarily be applicable or feasible in LMIC settings.

Additionally, conducting a comparison between LMICs and HICs will provide valuable insights into the contextual factors that influence the effectiveness of interventions and strategies across different settings. Such a comparison can inform the adaptation and implementation of interventions in diverse socio-economic contexts and contribute to the development of evidence-based policies and programs to promote adolescent physical activity globally.

This systematic review and meta-analysis will provide evidence-based recommendations for policymakers, educators, and public health practitioners to develop and implement effective strategies to promote adolescent physical activity in school and college settings.

1.3. Research Question Framed Using PICO

"In adolescents aged 10-19 years attending school and colleges in LMICs (P), how do school and college-based physical activity interventions (I) compared to no intervention(C) affect the physical activity levels, health knowledge, and fitness-related outcomes (O)?"

1.4. Objectives

- To identify the types of interventions and strategies used to promote physical activity among adolescents in school and college settings (10-19 years).

- To evaluate the impact of interventions implemented within school and college settings on enhancing physical activity levels and enhancing fitness among adolescents.

2. Methods

We will adhere to the 2020 Preferred Reporting Items for Systematic Reviews and Meta-Analyses (PRISMA) guidelines in conducting our systematic review. Our focus is to identify, assess, and analyse relevant studies conducted in school and college settings aimed at promoting physical activity or reducing sedentary behaviour among adolescents aged 10 to 19 years old.

2.1. Exclusion and Inclusion Criteria for considering studies for this review.

2.1.1. Types of studies:

The review will encompass randomized controlled trials (RCTs) conducted worldwide and published in English. However, studies lacking clarity in describing reference standards and those not primarily dedicated to increasing physical activity levels will be excluded. This means studies primarily focusing on nutrition or obesity reduction/prevention will not be considered.

2.1.2. Types of participants:

Studies involving adolescents typically aged 10-19 years, enrolled in middle or secondary schools and colleges.

2.1.3. Types of intervention/exposure:

Any intervention aimed at increasing physical activity among adolescents in school and college settings. This may include, but is not limited to, curriculum physical activity, structured physical education programs, extracurricular physical activities, integrated classroom-based activities, or school and college policy changes promoting physical activity. (24)(25)

- Physical education classes: Educating students on the advantages of physical activity and nutritious eating, the dangers of a sedentary lifestyle and poor dietary habits, and the significance of boosting their involvement in moderate to vigorous physical activity (MVPA) throughout the school and college day. Additionally, emphasizing the need for students to exert more energy during physical activity sessions.
- Curriculum interventions: Teacher preparation, educational resources, adjustments to the school and college day structure, and availability of exercise equipment were all part of the interventions. These interventions encompassed training sessions aimed at equipping teachers with more effective methods to encourage physical activity and integrate it into the curriculum. Additionally, training materials such as kits and packages containing resources for curriculum integration, as well as materials for students and parents, were provided. These packages comprised instructional guides on exercise, understanding bodily functions, and promoting healthy eating and nutrition.
- before and after school and college programs
- recess programming.
- active school and college travel
- classroom-based physical activity

2.1.4. Types of outcomes:

Primary Outcomes

- Proportion of students meeting recommendations for Moderate- intensity physical activity (MPA) (As defined by study authors)

- Proportion of students meeting recommendations for moderate to vigorous physical activity (MVPA) (As defined by study authors)
- Duration of Physical Activity
- Sedentary time

(For definition please refer to Table 1)

Secondary Outcomes

- Physical fitness
- Body mass index (BMI)
- Health-related quality of life

2.2. Eligibility Criteria (PICO framework)

**Population:** Adolescents (aged 10-19 years) who are students in school and college settings.
**Intervention:** Strategies and interventions implemented within school and college settings aimed at increasing physical activity among adolescents. This may include, but is not limited to, curriculum physical activity, structured physical education programs, extracurricular physical activities, integrated classroom-based activities, or school and college policy changes promoting physical activity.
**Comparator:**<br><br>- Studies with a control group. Comparators can include no intervention, standard school, and college curriculum without additional physical activity components. The difference between the two groups will only be physical activity intervention.
**Outcome:**<br><br>*Primary Outcomes*<br><br>• Proportion of students meeting recommendations for Moderate- intensity physical activity (MPA) (As defined by study authors)<br>• Proportion of students meeting recommendations for moderate to vigorous physical activity (MVPA) (As defined by study authors)<br>• Duration of Physical Activity<br>• Sedentary time<br><br>*Secondary Outcomes*<br><br>• Physical Fitness

- Body mass index (BMI)
- Health-related quality of life

2.3. Search methods for identification of studies.

2.3.1. Electronic search and search strategy:

This search will encompass various databases, including grey literature sources, electronic databases such as PubMed, CINAHL, Wiley Cochrane Library, ClinicalTrials.gov and Scopus. We will conduct a thorough literature search using the key terms outlined in Annex 1.

To develop our search criteria, we will formulate PICO questions and use them to create primary search terms. These primary search terms will consist of free-text terms (found in titles and abstracts), MeSH terms, and any other relevant subject indexing terms to ensure the retrieval of eligible studies.

2.3.2. Searching other sources:

We will review the reference lists of all included studies and pertinent systematic reviews to identify any studies that may not have been captured during the electronic search. Should there be any missing or unpublished data, we will reach out to the authors of relevant papers for additional information.

2.4. Data collection and analysis:

2.4.1. Selection of Studies:

For screening and management, reviewer along with supervisory team will assess the relevance of titles and abstracts from identified records. Full-text articles of potentially eligible studies will be obtained and evaluated for inclusion based on predetermined criteria. Any discrepancies between reviewers will be addressed through discussion or, if necessary, consultation with a external reviewer. Excluded literature titles and reasons for exclusion will be documented and analyzed during the full text screening stage. To ensure accurate reporting of the analysis, the study will adhere to the PRISMA 2020 Guidelines. The PRISMA flow chart (Figure 1) will provide an overview of the steps involved in the search and selection of relevant literature.

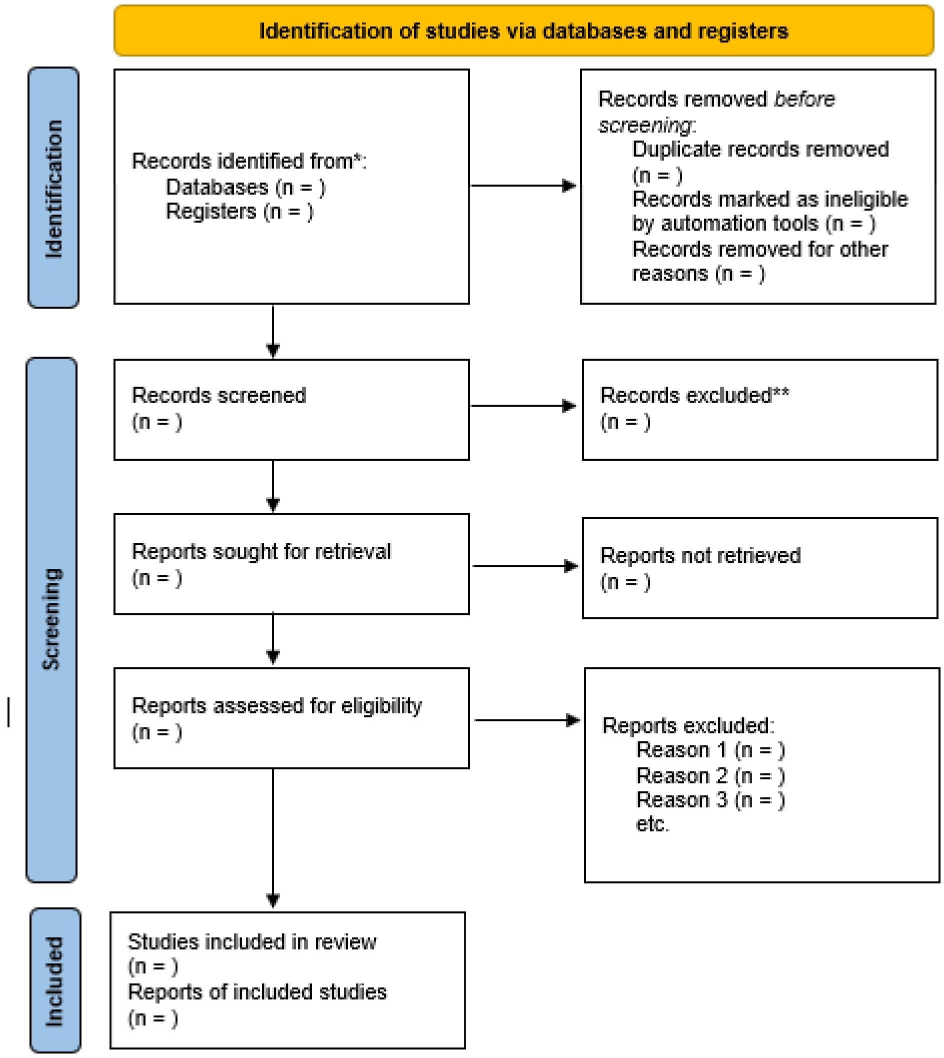

Figure 1:PRISMA Flowchart

2.5. Data Extraction and Management

Data extraction and management will involve collecting data from included full-text studies using a pre-formed data extraction sheet. Each team member's data extraction will be reviewed by another team member to ensure accuracy.

The risk of bias assessment will be conducted independently and in duplicate to mitigate subjective interpretation. Any discrepancies will be resolved through discussion, with involvement from a third review team member if necessary. Supervision by the study investigator will closely oversee literature selection, risk assessment, and data extraction. Clarifications or additional data required will be sought from the authors of the included studies.

Data will be extracted based on various study characteristics:

- Study methods: including journal, publication year, study design, duration, location, setting, and withdrawals.

- Participants: covering number, mean age, age range, gender, inclusion and exclusion criteria.

- Interventions: description, duration, and comparison group details.

- Outcomes: primary and secondary outcomes collected and reported time points.

- Additional information: including funding sources, study limitations, and conflicts of interest of authors.

2.5.1. Quality Assessment of Included Studies:

We will assess the risk of bias in all newly included studies across six domains:

- sequence generation
- allocation concealment
- blinding of participants and personnel
- blinding of outcome assessors
- incomplete outcome data
- selective reporting
- other bias

The evaluation will be conducted using the 'Risk of Bias 2' tool from the Cochrane Handbook for Systematic Reviews of Interventions to categorize the risk as low, high, or unclear. (26) Notably, incomplete outcome data will be assessed separately for device-based measures of physical activity and sedentary time, as well as for BMI and fitness, considering the common occurrence of missing data in studies employing accelerometers. Any discrepancies in assessments will be resolved through discussion. In cases where sufficient information is lacking, trial authors will be contacted to obtain missing data regarding the 'Risk of bias' items. (27)

2.5.3. Measures of Treatment Effect:

Data on post-intervention values adjusted for baseline differences, along with confidence intervals (95%) or standard deviations, will be extracted whenever available. If only changes from baseline are reported, these differences, along with confidence intervals or standard deviations, will also be extracted.

Whenever possible, multiple study groups will be combined into a single pairwise comparison using formulae for combining continuous data from multiple groups as provided in the Cochrane Handbook for Systematic Reviews of Interventions when separate data are presented for each group, or when between-group comparisons from multiple groups have independent control groups (28)

Meta-analysis will be conducted selectively, considering the relevance of combining interventions, participants, and associated clinical questions. This will ensure that only studies with sufficient similarity in these aspects are combined appropriately.

2.5.4. GRADE and Summary of Finding Tables

we will utilize the GRADE approach to assess the overall certainty of evidence for both primary and secondary outcome measures. This framework considers factors related to both internal and external validity to determine our confidence in the effect estimates presented. Any discrepancies in assessment will be resolved through discussion. For each outcome, we will categorize the certainty of evidence as very low, low, moderate, or high based on the GRADE domains as described in Chapter 14 of the Cochrane Handbook for Systematic Reviews of Interventions. (29)

After completing this evaluation, the subsequent step involves aggregating the outcomes from individual studies. Once the results are pooled, we will create GRADE evidence profiles and Summary of Findings (SoF) tables. These tables will illustrate the pooled estimates and the certainty of evidence for each outcome.

2.5.5. Sub-Group Analysis

Subgroup analysis will be conducted to explore potential variations in the effects of interventions aimed at increasing physical activity among adolescents in school and college settings, compared to no intervention, across different subgroups within the main study population, which comprises children aged 10 to 19 years. Our sub-group analysis will involve dividing participants into the following categories:

-World Bank analytical classification: low-income countries (LICs); lower middle income countries (LMICs); upper middle income countries (UMICs); and high income countries (or HICs).

- Types of interventions

- Duration of follow-up

Additionally, subgroup analysis will be utilized if statistical heterogeneity is observed among the studies included in our systematic review. This will help in addressing and resolving any discrepancies or inconsistencies present in the data.

2.6. Ethical Considerations

In conducting this systematic review, we will ensure respect for the confidentiality and informed consent of original study participants and maintain transparency and integrity by disclosing conflicts of interest and funding sources. We will ensure data integrity through accurate extraction and reporting, provide balanced interpretations, acknowledge study limitations, and respect cultural contexts. By adhering to these principles, our review will uphold ethical standards and contribute responsibly to scientific knowledge and public health policy.

3. Annexure

Annex 1: Potential search strategy

(“Adolescent” [MeSH] OR “Teens” OR “Youth”) AND (Physical Activity [MeSH] OR “Fitness” OR “Exercise” OR “Physical Education” OR “Physical training” OR “Sport” OR “Walk”) AND (“school” OR “Primary School” OR “Secondary School” OR “High School” OR “College”)

Adolescents	Physical Activity	Schools
(“Adolescent” [MeSH] OR “Teens” OR “Youth”)	(Physical Activity [MeSH] OR “Fitness” OR “Exercise” OR “Physical Education” OR “Physical training” OR “Sport” OR “Walk”)	(“school” OR “Primary School” OR “Secondary School” OR “High School” OR “College”)

Table 1

Glossary of terms (30)

TERM	DEFINITION
Metabolic equivalent of task (MET)	The metabolic equivalent of task, or simply metabolic equivalent, is a physiological measure expressing the intensity of physical activities. One MET is the energy equivalent expended by an individual while seated at rest, usually expressed as mLO^2/kg/min.
Moderate- intensity physical activity (MPA)	On an absolute scale, moderate-intensity refers to the physical activity that is performed between 3 and <6 times the intensity of rest (METs). On a scale relative to an individual's personal capacity, MPA is usually a 5 or 6 on a rating scale of perceived exertion scale of 0–10.
Moderate-to-vigorous intensity physical activity (MVPA)	On an absolute scale, MVPA refers to the physical activity that is performed at >3 METs (ie, >3 times the intensity of rest). On a scale relative to an individual's personal capacity, MPA is usually a 5 or above on a scale of 0–10.
Sedentary behaviour	Any waking behaviour characterised by an energy expenditure of 1.5 METs or lower while sitting, reclining or lying. Most desk-based office work, driving a car and watching television are examples of sedentary behaviours; these can also apply to those unable to stand, such as wheelchair users. The guidelines operationalise the definition of sedentary behaviour to include self-reported low movement sitting (leisure time, occupational and total), TV viewing or screen time and low levels of movement measured by devices that assess movement or posture.
Sedentary screen time	Time spent watching screen-based entertainment while sedentary, either sitting, reclining or lying. Does not include active screen-based games where physical activity or movement is required.

4.References

1. https://www.who.int/news-room/fact-sheets/detail/physical-activity
2. https://www.ncbi.nlm.nih.gov/pmc/articles/PMC2885312/
3. https://pubmed.ncbi.nlm.nih.gov/23467962/
4. Chaput, JP., Willumsen, J., Bull, F. *et al.* 2020 WHO guidelines on physical activity and sedentary behaviour for children and adolescents aged 5–17 years: summary of the evidence. *Int J Behav Nutr Phys Act* **17**, 141 (2020). https://doi.org/10.1186/s12966-020-01037-z
5. Tremblay MS, LeBlanc AG, Kho ME, Saunders TJ, Larouche R, Colley RC, Goldfield G, Connor Gorber S. Systematic review of sedentary behaviour and health indicators in school-aged children and youth. Int J Behav Nutr Phys Act. 2011 Sep 21;8:98. doi: 10.1186/1479-5868-8-98. PMID: 21936895; PMCID: PMC3186735.
6. https://doi.org/10.1016/S0140-6736(12)61031-9
7. https://www.who.int/health-topics/physical-activity#tab=tab_2
8. https://doi.org/10.1016/j.jshs.2022.01.007
9. https://doi.org/10.1016/S2352-4642(19)30323-2
10. Drummond, M., Drummond, C., & Dollman, J. (2017). Physical activity from childhood to adolescence: A systematic review of longitudinal studies. *Sports Medicine*, 47(12), 2513-2525.
11. Brodersen, N. H., Steptoe, A., Boniface, D. R., & Wardle, J. (2007). Trends in physical activity and sedentary behaviour in adolescence: ethnic and socioeconomic differences. *British Journal of Sports Medicine*, 41(3), 140-144.
12. https://doi.org/10.1016/j.ypmed.2019.105819
13. Neil-Sztramko SE, Caldwell H, Dobbins M. School-based physical activity programs for promoting physical activity and fitness in children and adolescents aged 6 to 18. Cochrane Database of Systematic Reviews 2021, Issue 9. Art. No.: CD007651. DOI: 10.1002/14651858.CD007651.pub3. Accessed 27 February 2024.
14. Janssen, I., & Leblanc, A. G. (2010). Systematic review of the health benefits of physical activity and fitness in school-aged children and youth. *International Journal of Behavioral Nutrition and Physical Activity*, 7, 40.
15. Telama, R. (2009). Tracking of physical activity from childhood to adulthood: A review. *Obesity Facts*, 2(3), 187-195.
16. Hardman, K., & Marshall, J. J. (2000). World-wide survey of school physical education. *Final Report*. UNESCO.
17. Naylor P, McKay HA Prevention in the first place: schools a setting for action on physical inactivity *British Journal of Sports Medicine* 2009;**43:**10-13.
18. Demetriou, Yolanda & Höner, Oliver. (2012). Physical activity interventions in the school setting: A systematic review. Psychology of Sport and Exercise. 13. 186–196. 10.1016/j.psychsport.2011.11.006.
19. Sallis, J. F., Prochaska, J. J., & Taylor, W. C. (2012). A review of correlates of physical activity of children and adolescents. *Medicine & Science in Sports & Exercise*, 34(5), 963-975.
20. https://iris.who.int/bitstream/handle/10665/363607/9789240059153-eng.pdf?sequence=1

21. Kriemler, S., Meyer, U., Martin, E., van Sluijs, E. M., Andersen, L. B., & Martin, B. W. (2011). Effect of school-based interventions on physical activity and fitness in children and adolescents: a review of reviews and systematic update. *British journal of sports medicine*, *45*(11), 923–930. https://doi.org/10.1136/bjsports-2011-090186
22. Jacob, C.M., Hardy-Johnson, P.L., Inskip, H.M. *et al.* A systematic review and meta-analysis of school-based interventions with health education to reduce body mass index in adolescents aged 10 to 19 years. *Int J Behav Nutr Phys Act* **18**, 1 (2021). https://doi.org/10.1186/s12966-020-01065-9
23. Valter Cordeiro Barbosa Filho, Giseli Minatto, Jorge Mota, Kelly Samara Silva, Wagner de Campos, Adair da Silva Lopes, Promoting physical activity for children and adolescents in low- and middle-income countries: An umbrella systematic review: A review on promoting physical activity in LMIC, Preventive Medicine, Volume 88, 2016, Pages 115-126, ISSN 0091-7435, https://doi.org/10.1016/j.ypmed.2016.03.025.
24. Watson, A., Timperio, A., Brown, H., Best, K., & Hesketh, K. D. (2017). Effect of classroom-based physical activity interventions on academic and physical activity outcomes: a systematic review and meta-analysis. *The international journal of behavioral nutrition and physical activity*, *14*(1), 114. https://doi.org/10.1186/s12966-017-0569-9
25. Singh, A., Bassi, S., Nazar, G. P., Saluja, K., Park, M., Kinra, S., & Arora, M. (2017). Impact of school policies on non-communicable disease risk factors - a systematic review. *BMC public health*, *17*(1), 292. https://doi.org/10.1186/s12889-017-4201-3
26. https://training.cochrane.org/sites/training.cochrane.org/files/public/uploads/resources/Handbook5_1/Chapter_8_Handbook_5_2_8.pdf
27. https://training.cochrane.org/handbook/current/chapter-07
28. https://training.cochrane.org/handbook/current/chapter-06
29. https://training.cochrane.org/handbook/current/chapter-14
30. Bull FC, Al-Ansari SS, Biddle S, Borodulin K, Buman MP, Cardon G, Carty C, Chaput JP, Chastin S, Chou R, Dempsey PC, DiPietro L, Ekelund U, Firth J, Friedenreich CM, Garcia L, Gichu M, Jago R, Katzmarzyk PT, Lambert E, Leitzmann M, Milton K, Ortega FB, Ranasinghe C, Stamatakis E, Tiedemann A, Troiano RP, van der Ploeg HP, Wari V, Willumsen JF. World Health Organization 2020 guidelines on physical activity and sedentary behaviour. Br J Sports Med. 2020 Dec;54(24):1451-1462. doi: 10.1136/bjsports-2020-102955. PMID: 33239350; PMCID: PMC7719906.
